# Challenging the Database: Day-of-Analysis Calibration and UF Modeling for Reliable RRF Use in Medical Device Chemical Characterization

**DOI:** 10.1021/acs.analchem.5c04247

**Published:** 2025-10-08

**Authors:** Michael Rush, J. David Ricker, Pramod Prasad Poudel, Nuwan Kothalawala, Cary Watterson, Dmitriy Pastarnak

**Affiliations:** Edwards Lifesciences, 12050 Lone Peak Parkway, Draper, Utah 84020, United States

## Abstract

Accurate quantitation during chemical characterization—also referred to as extractables and leachables (E&L)—within a toxicological risk assessment for medical device biocompatibility hinges on the appropriate application of relative response factors (RRFs). This study investigates the variability of RRFs across a chemically diverse set of compounds and evaluates the implications of quantitation model selection on analytical outcomes. Using gas chromatography–mass spectrometry (GC–MS) and liquid chromatography–mass spectrometry (LC–MS), we demonstrate that RRFs are highly context-dependent and influenced by factors such as ionization mode, compound class, concentration, and instrument conditions. We propose an approach to RRF determination, emphasizing day-of-analysis calibration and uncertainty factor modeling to improve reproducibility. Our findings support a statistically grounded application of analytical evaluation thresholds, enhancing the reliability of semiquantitative assessments in medical device biocompatibility.

## Graphical abstract

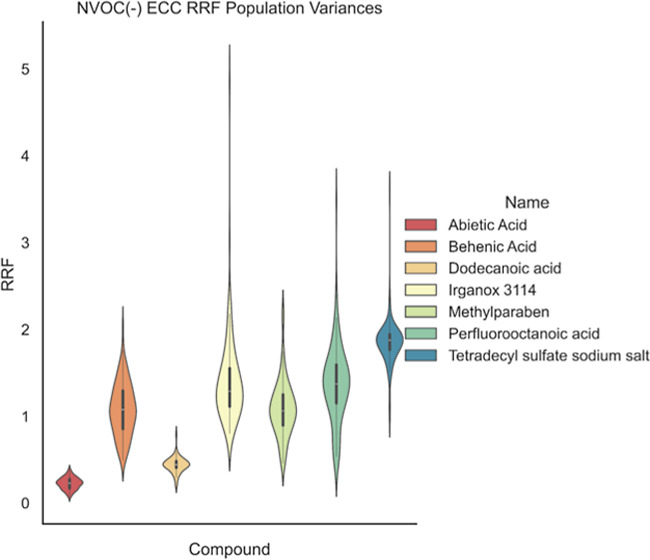

Medical devices are composed of diverse materials that may pose both acute and chronic risks to patients during their use and lifetime. To understand that potential risk, a set of international standards titled ISO10993 Biological evaluation of medical devices^1^ provides a framework for evaluating the biological safety of these materials based on the nature and duration of patient contact. Historically, many biological end points required in vivo testing; however, the global push to implement the “3Rs” (Replacement, Reduction, and Refinement) has accelerated the adoption of alternative approaches.^2^ As a result, chemical characterization has become increasingly central to biocompatibility assessments. ISO10993-18 outlines the principles and practices of chemical characterization for medical device biocompatibility. Although interpretations of ISO10993-18 vary across laboratories and regulatory agencies, it remains the foundational standard for chemical characterization in most regulatory submissions.

This standard applies broadly across medical devices used in diverse therapeutic contexts. These devices are manufactured from a wide array of materials and often incorporate processing aids, which complicate solvent selection for the exhaustive extraction procedures prescribed in ISO10993-18. Moreover, patients may be exposed to a wide chemical space, necessitating the use of multiple analytical techniques. These include ICP–MS for elemental analysis, headspace GC–MS for volatiles and residual solvents, direct-injection GC–MS for semivolatile organic compounds, and LC–MS for nonvolatile organic compounds. The nature and duration of patient contact establish the initial reporting threshold, which is derived from a dose-based toxicological threshold. This threshold is further refined by extraction conditions and clinical exposure scenarios to establish the analytical evaluation threshold (AET), the concentration above which all extractable compounds must be identified and quantified.

The release of the ISO10993-18:2020 ^3^ and ISO10993-18:2020 Amd1:2022 ^4^ introduced the concept of adjusting the AET with an uncertainty factor (UF) which accounts for the “uncertainty of the analytical method”.^3^ The FDA Chemical Analysis for Biocompatibility Assessment of Medical Devices,^5^ a draft guidance, provides additional context upon the analytical evaluation threshold and the usage of UF; we recognize this is draft guidance which is subject to potential change. The UF adjusts the concentration threshold at which all extractable compounds with concentrations above the threshold are required to be identified and quantified. As the UF adjusts the AET lower, the number of reportable extractable compounds may increase dramatically, particularly for permanent patient-contacting implantables with large surface areas and subsequently large extraction volumes.

There have been various approaches recommended to determine the UF. The two published approaches are establishing an external database of response factors or relative response factors (RRFs) and subsequently determining the UF per method^5,6^ or establishing the external data set of RRFs for an analytically appropriate method to determine the UF from the compilation of RRFs across all methods.^7^ Figure 1 describes two approaches for calculating RRFs at a specific concentration. Alternatively, the RRF can be determined from the first-order coefficient of a linear least-squares fit to the instrument response across the dynamic range, assuming an acceptable goodness-of-fit.^5^ The general workflow for building an external database of RRFs is as follows: an authentic standard is prepared at a detectable concentration in a solvent compatible with the analytical methodology. The standard is analyzed by various techniques—e.g., headspace-EI-GCMS for volatiles, direct inject EI-GCMS for semivolatiles, and direct inject ESI-LCMS for nonvolatiles. Additional detectors may be in-line or split effluent post chromatographic separation, such as Corona CAD or UV/Vis in-line with the LC–MS or split effluent for FID with GCMS. In general, ESI is the primary ionization source for LCMS and APCI is utilized less frequently.^8^ Additionally, HRAM-GCMS may be utilized when additional mass spectral information is required for identifications. The RRF is then calculated based on the laboratory’s strategy and documented for future use in AET calculations.

**Figure 1:**
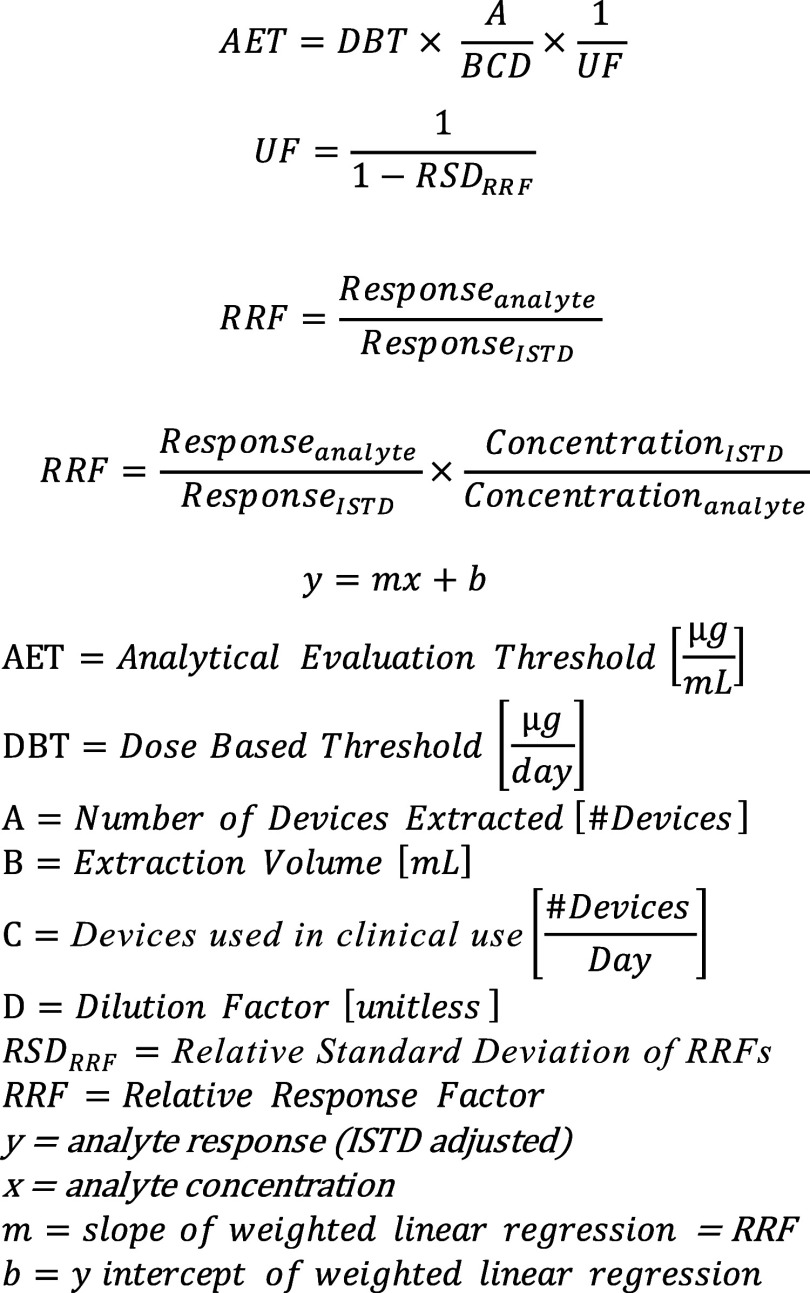
Calculations required to set the analytical evaluation threshold, which is determined first by extraction parameters and then modified by the uncertainty factor which is derived from the relative standard deviation of RRFs of authentic reference standards.

There is a lack of consensus about the approaches of an external database. Current chemical characterization for biocompatibility publications typically report RRFs of these standards at concentrations ranging from 10 to 50 μg/mL, often based on a single measurement.^9,10^ Some do not publish their reported concentrations,^7^ nor provide identities of the chemicals analyzed. Limited literature exists for RRFs below 5 μg/mL.^11,12^ As noted in those publications, analytical evaluation thresholds may be substantially lower for permanent implantable devices with large surface areas and, subsequently, large extraction volumes. Zdravkovic et al.^10^ derivatize their chemicals for RRF determination; however, they did not report the requisite evaluation of derivatization efficiency and how it might be utilized in regards to semiquantitation of nontarget extractables (NTEs) where the degree of derivatization is unknown. The FDA released the Chemical List for Analytical Performance (CLAP)^13^ Regulatory Science Tool which is a list of 106 commonly reported extractables and their RRFs across GC–MS and LC–MS.^14^ Jenke et al.^15^ and the FDA CLAP RST^14^ analyzed a subset of these chemicals utilizing a splitless GC–MS method at 20–50 and 5–20 μg/mL, respectively, which may cause column overload and detector saturation—potentially adding uncertainty to RRF values.^9^ ISO10993-18 and FDA draft guidance cite publications which recommend an external database of RRFs to contain at least 200–500 chemicals^5^ or representing a wide range of physical chemical properties.^16^

Many chemical characterization publications related to medical device biocompatibility^6–12,17–24^ do not report the quantitation model used to evaluate reference standard chemicals in the mass spectrometric analysis. The FDA CLAP does report that it uses TIC for GCMS data and EIC(s) for LCMS data. Additionally, the RRFs of the chemicals are typically reported as single values—often derived from a single measurement^9^—rather than being presented as a function of population statistics across the analytical systems. The nature of the quantitation model is critical to the process of quantitation, including semiquantitative approaches that apply surrogate calibrations to NTEs. As the quantitation model directly affects the RRF, it should be fit-for-purpose and account for the characteristics of the NTEs, which may vary as a function of the ionization and detection systems.

In this work, we present the results of long-term monitoring of relevant extractable chemicals and their RRFs which reasonably represent the expanded chemical space of both expected chemical extractables, as determined by a priori information, and frequently observed extractables in Edwards Lifesciences products. At Edwards Lifesciences, while we often customize our reference standards to meet analytical challenges for the unique expanded chemical spaces of our devices, these selected compounds provide an opportunity for the evaluation of variability in chemical characterization workflows. This work will demonstrate the role of quantitation model selection and how it can bias RRFs, the importance of incorporating population statistics into RRF modeling, the unequal variance of the monitored chemicals’ RRFs, and how to mitigate the variability of these effects. We then discuss the impacts of these biases in RRFs, UF, and subsequent AET determinations and the failures of relying on an external database model for these evaluations.

## Experimental Section

### Semivolatile Organic Compound (SVOC) Analysis by GC–MS

SVOC analysis was performed by using an Agilent 7890 or 8890 gas chromatograph coupled with an Agilent 5977B or C mass selective detector (MSD) equipped with a high-efficiency source for electron ionization. Separation was achieved on an Agilent DB-35MS UI column (20 m × 0.180 mm i.d., 0.18 μm film thickness) using helium as the carrier gas at a constant flow rate of 0.5 mL/min. The GC oven was initially held at 50 °C for 5 min, then ramped to 340 °C at 10 °C/min, and held for 6 min. Following each run, the oven was cooled and held at 50 °C for 3 min to prepare for the next injection. Samples (0.75 μL) were introduced in pulsed splitless mode at an inlet temperature of 225 °C. The inlet liner is Single-Taper Splitless with a glass wool Ultra Inert Inlet Liner from Agilent. The MSD transfer line was maintained at 335 °C. Data were acquired in profile mode with a scan range of *m*/*z* 35–500. The ion source and quadrupole temperatures were set to 230 and 150 °C, respectively. The electron energy was 70 eV, and the emission current was 100 μA. A solvent delay was applied at 6.5 min.

### Nonvolatile Organic Analysis/LCMS

Three Agilent 6545 and two Agilent 6546 LC-QTOF instruments were used for the analysis of LCMS(±) extractables. Chromatographic separation was performed using an Agilent Poroshell 120 EC-C18 column (2.1 × 100 mm, 1.9 μm) at a flow rate of 0.4 mL/min. Injection volumes were 1.25 μL for positive mode and 1.5 μL for negative mode. The aqueous mobile phase consisted of water with 5 mM ammonium formate and 0.01% (v/v) formic acid. The organic mobile phase was composed of 50% (v/v) methanol in acetonitrile with 5 mM ammonium formate and 0.01% (v/v) formic acid for positive mode or 0.25 mM ammonium formate and 0.01% (v/v) formic acid for negative mode. A linear gradient ramped from 5% to 100% organic by 12 min, held at 100% until 28 min, followed by re-equilibration at 5% organic for 2 min. The column temperature was maintained at 50 °C. Data was collected in either positive or negative polarity without any polarity switching, using profile mode and MS^1^-only acquisition to maximize time for the scan during the duty cycle. A second injection was used for MS^2^ data acquisition to confirm compound identities and provide fragmentation data for nontargeted extractables (NTEs). The scan range was *m*/*z* 100–1700. Mass accuracy was calibrated to <0.5 ppm using standard Agilent tuning masses, and resolution was maintained at ≥10,000 for all monitored analytes.

The ion source was a Dual AJS ESI with the following parameters: gas temperature 250 °C, gas flow 12 L/min, sheath temperature 350 °C, capillary voltage (*V*_cap_) 3500 V, and nozzle voltage 500 V for positive mode and 2000 V for negative mode.

### Chemicals and Standards

GC–MS-monitored standards and internal standards were custom-compounded by Restek at concentrations of 500 and 1000 μg/mL in ethyl acetate, respectively. LC–MS-monitored chemicals were primarily sourced from Sigma Millipore. Analytical standards for LC–MS were prepared as stock solutions at 50 and 25 μg/mL in isopropyl alcohol. Internal standards for LC–MS were prepared at a concentration of 25 μg/mL and diluted to match the dynamic range of each method. These isotopically labeled internal standards were spiked at the midpoint of the dynamic range and used consistently throughout the analysis to correct the baseline signal variation.

All solvents used were of LC–MS grade, and ultrapure water (18.2 MΩ·cm resistivity) was obtained from a Milli-Q IQ 7005 integrated water purification system. Tinuvin 326 (CAS# 3896-11-5) and Antioxidant 852 (CAS# 154862-43-8) were purchased from Sigma. Additional details are provided in the Supporting Information.

Data Acceptance: The instrumentation for both GCMS and LCMS was tuned and calibrated prior to use. System reproducibility (<20% injection RSD) and linearity (*R* ^2^ > 0.95 with weighted 1/*x* ^2^ for GCMS and 0.97 for LCMS with weighted 1/*x* for dynamic ranges) were established for each monitored chemical to ensure quality data collection. This criterion was excluded from the expanded dynamic ranges analysis.

### Software and Data Processing

GC–MS and LC–MS data were acquired using Agilent MassHunter Acquisition software (GC–MS: version 10.2.489.0; LC–MS: Version 10.1, Build 10.1.48). Mass spectral data were processed using Agilent MassHunter Quantitative Analysis (version 10.2, Build 10.2.733.8) and Qualitative Analysis (version 10.0 Service Release 1, Build 10.0.10305.10). GC–MS data were deconvoluted using the “Find by Chromatogram Deconvolution” function with a retention time (RT) window size factor of 100 and a signal-to-noise (S/N) threshold of 0. The *m*/*z* 28 ion was excluded from analysis. These parameters were adjusted as needed based on data quality.

LC–MS data were processed using Agilent MassHunter Profinder (version 10.0.2) and Explorer (version 1.0) via batch recursive feature extraction. Deconvolution parameters were set using the widest observed mass error (ppm) and RT window across all monitored compounds. Positive and negative ionization mode data were processed separately to account for the mode-specific adduct formation. Manual review was conducted to assess whether standard adduct (+H, +NH_4_, +Na, +K, +C_2_H_7_N_2_ in positive mode; −H, +Cl, +HCOO in negative mode), dimers, trimers, and multiple charge states were sufficient. If additional adducts or settings were required, data was reprocessed accordingly. Figures and plots were generated in Microsoft Excel and python 3.12 with pandas, seaborn, matplotlib, numpy, and scipy packages.

### Safety

There are no new safety hazards associated with this research.

## Results and Discussion

Quantitation modeling: the first and fundamental step to establishing RRFs for chemicals in a method should be establishing the appropriate quantitation model for mass spectrometry data. The most common quantitation models are total ion chromatogram (TIC), base peak chromatogram (BPC), extracted ion chromatogram (s) (EIC(s)), and deconvoluted extracted compound chromatogram (ECC). This term may be described differently by various vendors or programs; we will refer to ECC as the deconvoluted spectra of all ions associated with a chemical, including adducts, dimer or trimer ions, and all isotopes, to capture the entire ionization. We will describe these processes for the MS1 data; however, for methods that utilize MS2 signals to quantitate, the principles of the quantitation model are the same regardless of mass spectral resolution. TICs or BPCs quantitation modelling may work for chromatograms with minimal background and limited coeluting peaks. However, BPC modelling will only produce a response of the most abundant ion; therefore, it is not a capture of the complete response of an analyte, thus negatively biasing the RRF. For multimaterial and complex geometry permanent patient-contacting medical devices with large extraction volumes, TIC and BPC are not suitable quantitation models given the low AET, which is frequently below the background signal in the TIC and BPC. EIC(s) modelling provide the selectivity of signals; however, selecting the ions is often a laborious, manual process and prone to human error. It is also important to consider the nature of semiquantitation. Using surrogate chemicals to quantitate other chemicals, even if they are in the same chemical class, means the unique ionization patterns for the surrogate standard can bias the nontarget extractable reported concentration when those patterns diverge. Figures 2–4 show examples drawn from the FDA CLAP common extractables where selecting ions to monitor can be challenging. The figures are labeled with the same RM nomenclature used by the FDA CLAP.^13,14^ There are several EIC strategies: base peak, monoisotopic ions, adducts, etc. The manual selection, prone to human error, variability, and bias, of ions to monitor for quantitation is challenging when the number of reportable compounds becomes unwieldy, so another option is to utilize deconvolution algorithms to peak pick and generate the associated spectra. These deconvoluted spectra can capture a more total response of a compound compared to a selected ion(s). These algorithms are usually unique to software and vendors; however, they are often based on the mass tolerances, retention time windows, and peak shape to ensure the deconvolution captures the complete ionization in their ECC.^25–28,30^ There are many complex selections and practices in deconvolution and significant challenges to implement appropriately; the purpose of this work is to not explore those, rather to show the effect of the quantitation model on the RRF. The EIC model shows bias most significantly when semiquantitating. For example, the EI-GCMS analysis of BHT and methyl oleate have different RRFs as a function of the quantitation model. The single ion base peak EIC produces an RRF less than a quarter of methyl oleate; this same effect is minimal in BHT. This is a function of the ionization tendencies of individual chemicals and instrumental parameters; BHT does not fragment as much as methyl oleate in electron ionization, so the EIC quantitation model bias is smaller on chemicals with minimal fragmentation. The EIC model dramatically underreports the relative response of methyl oleate, as it has considerable fragmentation. Depending on the quantitation model selected, the RRF can be biased as a function of the gas phase chemistry during the various types of ionization—e.g., electron versus chemical ionization. This is also an issue with HRAM mass spectrometry data given the frequency of adduct formation and in-source fragmentation. Base peak EIC selection is biased against compounds that form multiple adducts. Figure 3 shows the effect of base peak EIC versus ECC for dilauryl thiodipropionate (DTDPP); this compound forms multiple adducts in positive mode. The EIC model biases the RRFs downward, while the ECC model has a larger RRF. Additionally, the relative ratio of each adduct can change as a function of instrumental parameters, instrument health, potential contaminants or interferents, and the sample matrix or sample itself.^29^ Supporting Information SI4 shows how the relative ratio of different adducts for the same compound, DTDPP, can change drastically in data collected at different days on the same instrument—in this example, the predominate adduct changes from [M + Na]^+^ to [M + NH_4_]^+^ to [M + ACN + NH_4_]^+^. This common adduct formation makes single ion EIC modelling of RRFs—and subsequent UF determination—a highly erroneous approach. Using multiple EICs for each monoisotopic ion for each adduct may help the issues; however, compounds with significant isotopic distributions will have reduced RRFs from the bias of the quantitation model. Figure 4 shows this effect on hexadecamethylcyclooctasiloxane (D8); note that this chemical does not have a CLAP RM designation. The EIC model biases the RRF downward with significant isotopic distribution beyond the monoisotopic ion; for the RRF of D8, the monoisotopic EIC results in less than half of the total response, compared to ECC. This effect is pronounced with the presence of elements with complicated and significant isotopic abundances, such as chlorine, bromine, or silicon. The biasing of the RRF can also occur with formula CvHwNxOyPz when the ^13^C isotopes become a significant portion of the ion series. See Supporting Information SI3 for Tinuvin 326 CAS# 3896-11-5 and Antioxidant 852 CAS# 154862-43-8 for examples in which a monoisotopic EIC would negatively bias RRFs.

**Figure 2:**
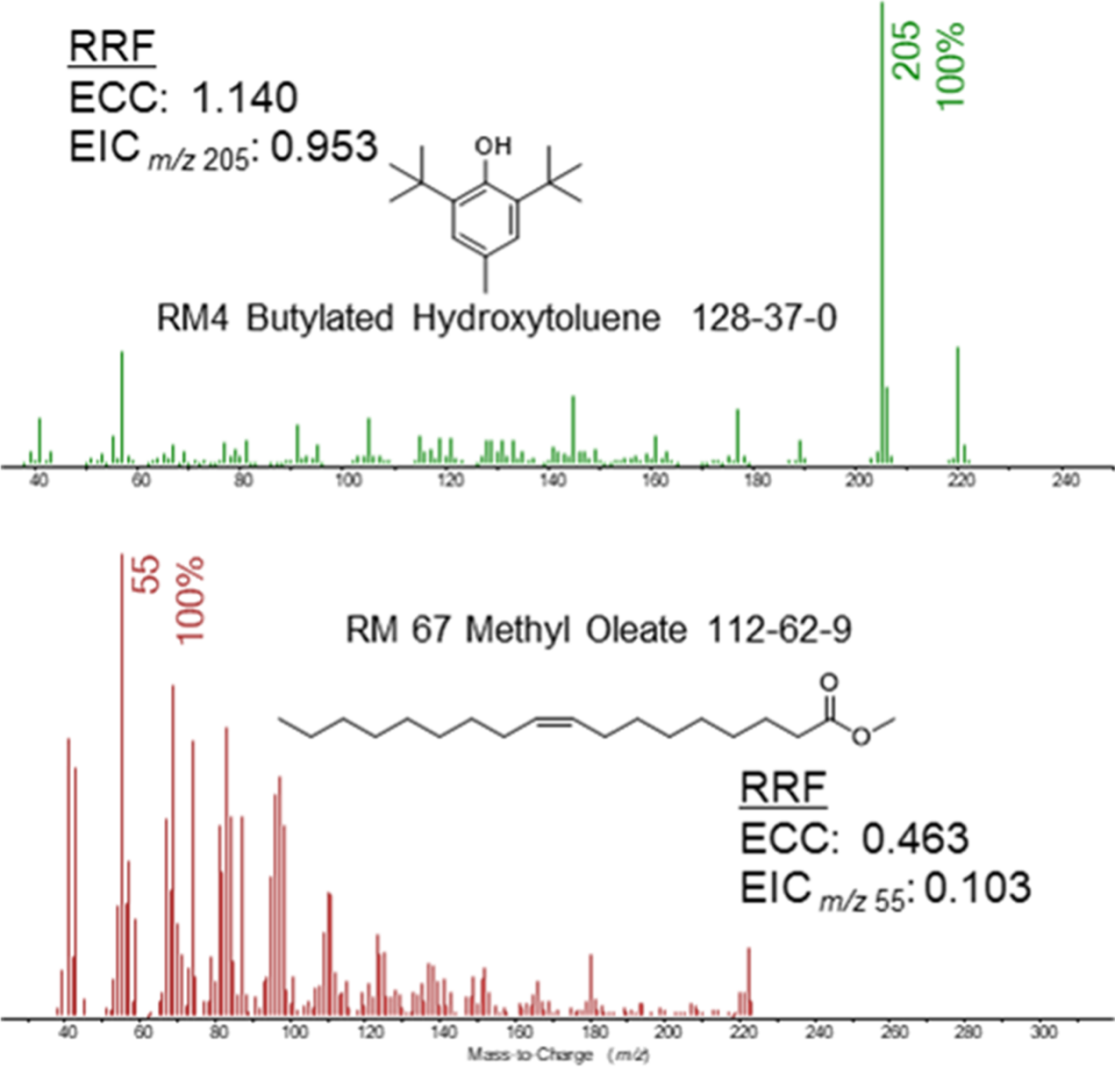
EI-GCMS spectra of two commonly observed extractables. Selecting ions for quantitation for RRF determination can be problematic if compounds fragment during ionization to varying degrees.

**Figure 3:**
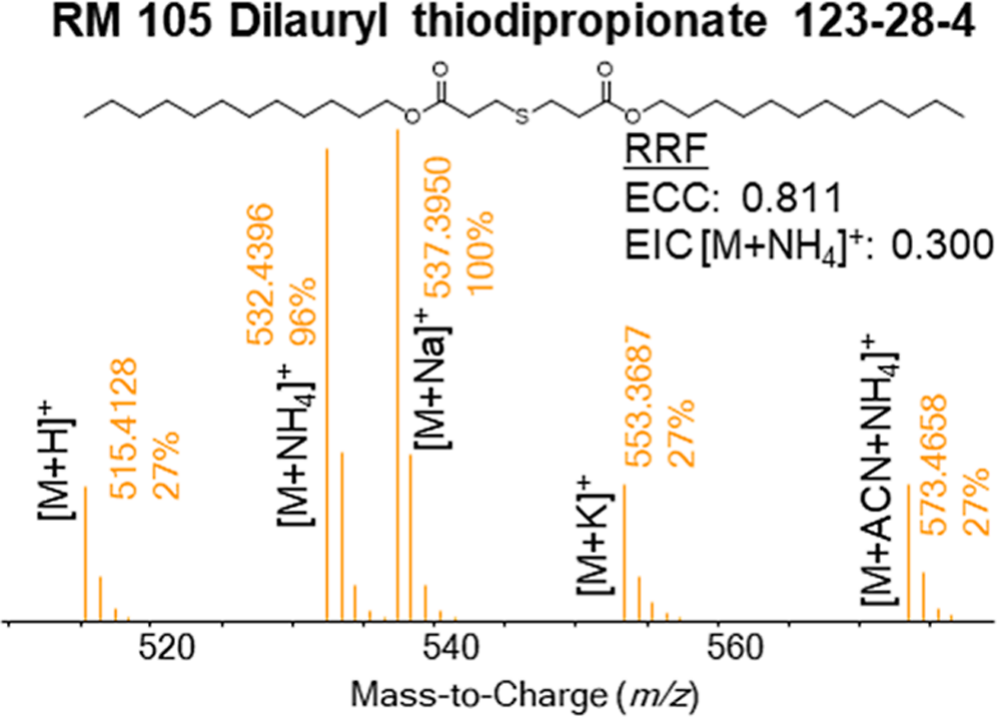
HRAM-ESI-LCMS (+) spectra of DTDPP. Selecting a single ion for EIC would ignore a significant number of adducts which would negatively bias the observed RRF.

**Figure 4:**
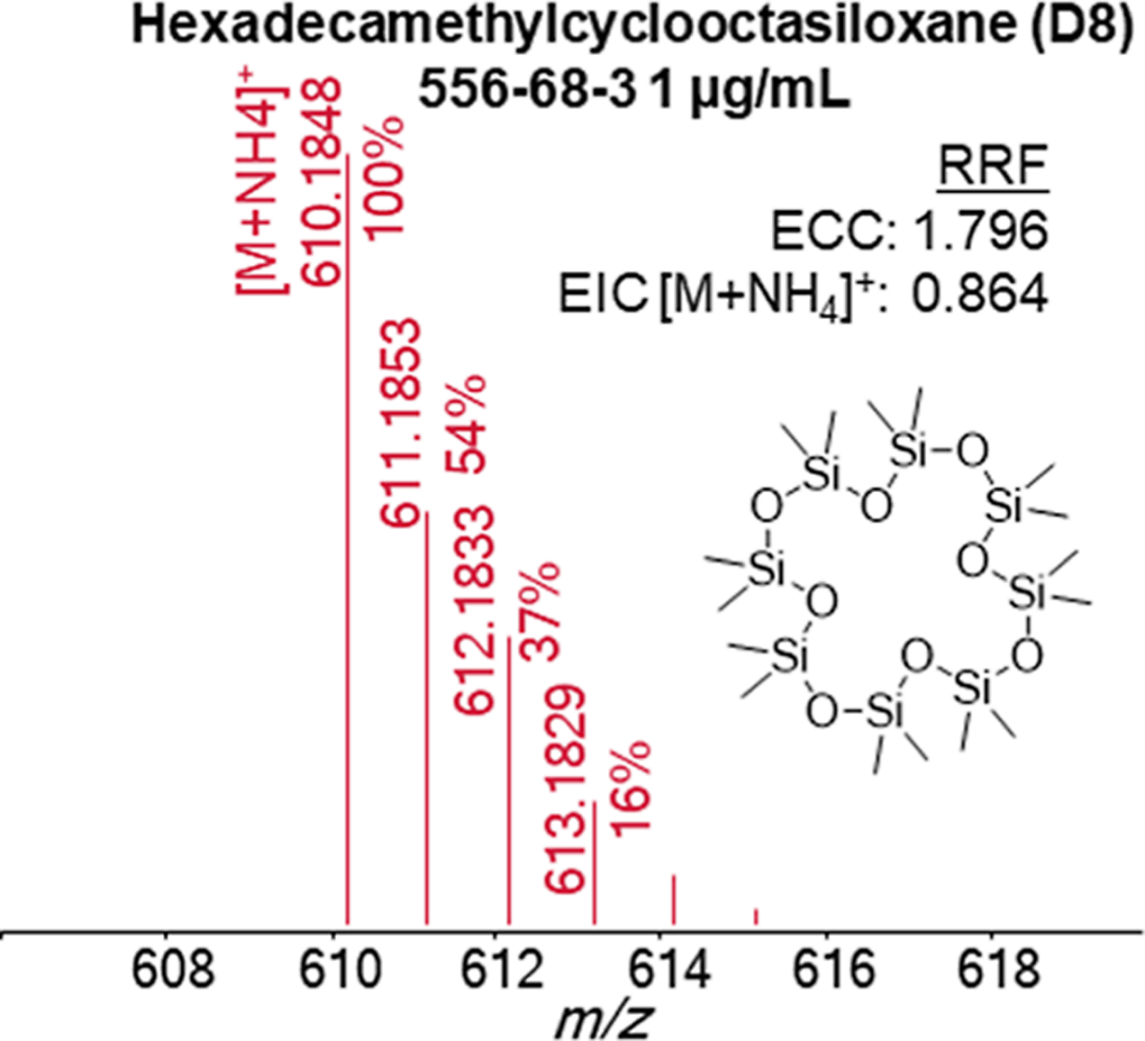
Isotopic distribution of D8 shows a monoisotopic EIC would reduce its RRF in half.

The effect of quantitation modeling on RRFs is amplified during semiquantitation. If the EIC(s) model is applied, the RRF biases are applied to the extractable with a different isotopic model. For ECC HRAM data with soft ionizations such as ESI or APCI, the structure elucidation specialists need to identify, at minimum, the adducts, mass error thresholds, potential in source fragmentation, and isotopic distributions and how they might differ compared to the surrogate standard. As shown in Figure 5, if 0.150 μg/mL eicosane is reported as an NTE and chrysene was the surrogate—both are corrected with dibutyl phthalate-3,4,5,6-D4 as an ISTD—the semiquantitated concentration of the ECC approach is significantly higher than the single ion EIC model. This reported concentration by the ECC semiquantitation model is closer to the actual concentration. This issue with semiquantitation can occur for all sorts of organic molecules when using an EIC approach. For example, if a standard with a monoisotopic EIC that has a single chlorine isotopic distribution—e.g., Tinuvin 326—is applied to an extractable with an isotopic distribution of two chlorines, the semiquantitated concentration would be biased negatively. Semiquantitation of extractables by the surrogate standard with differing isotopic distributions will bias results. It is critical for the quantitation model to be fit-for-purpose and minimize the error associated with semiquantitation for the quantitative toxicological risk evaluation. We are not advocating that chrysene is the best surrogate to use for eicosane as an NTE, but rather to show the implication of the quantitation model on reported quantities.

**Figure 5:**
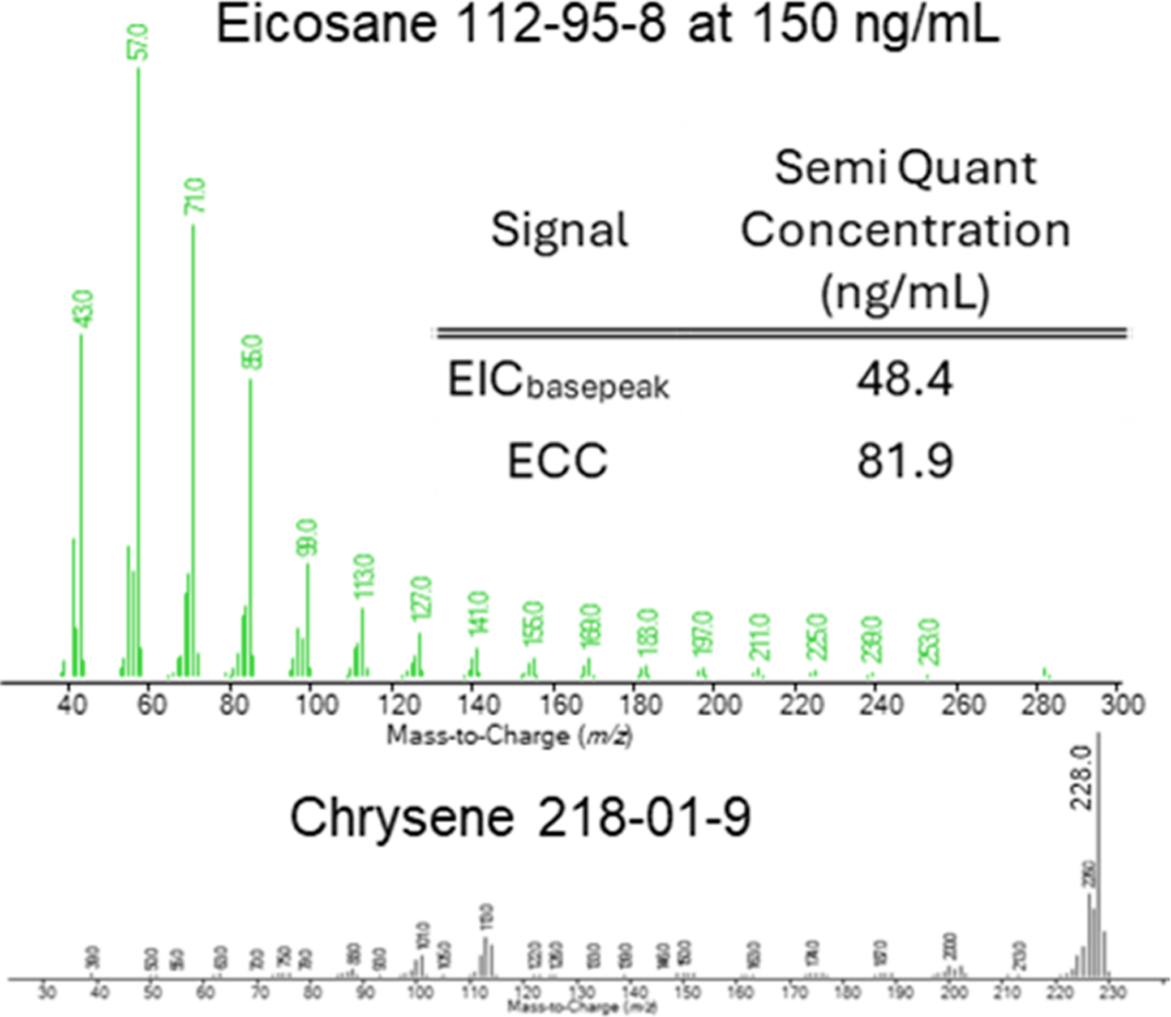
Quantitation model can dramatically bias the semiquantitated concentrations. This bias can lead to compounds being below AET and not reportable or the quantities are underreported. Unreported or underreported extractables lead to an inaccurate quantitative toxicological risk assessment.

### Dynamic Ranges and RRFs

Flohl et al.^12^ have considered the nature of RRFs and dynamic ranges of their analytical methodology. Given the exaggerated and exhaustive extractions, NTEs can be reported at a wide range of concentrations, so determining the RRF should be relevant to the analytical methodology. In essence, using an RRF for a compound in the 50 μg/mL range may not represent the RRF of that same compound at low ng/mL. Often RRFs are determined from a single concentration^9^ vide supra. To show the effect of the concentration on RRF, we analyzed the SVOC- and NVOC-monitored compounds across a wide concentration range: SVOC ranged from 0.025 to 10 and NVOC from 0.0005 to 50 μg/mL. The data and calibration plots are given in Supporting Information. Figures 6 and 7 are SVOC and NVOC examples showing the change in RRF as a function of the concentration range. Table 1 shows examples of RRFs from these calibration curves; two are derived from the slopes of the ranges of concentration, while the maximum point is determined at a single concentration. Two dynamic ranges are drawn with one in the nominal dynamic range (0.025–1 μg/mL for LCMS(±) and 0.025–0.250 μg/mL for GCMS) and one greater than 1 μg/mL for each chemical across LCMS­(+), LCMS(−), and GCMS. These are built from the ECC response which is internal standard corrected: LCMS­(+) triethyl phosphonoacetate ^13^C_2_, LCMS(−) sodium dodecyl-d25 sulfate, and GCMS acetophenone-d8. Supporting Information SI5 contains the log–log plots, plots showing the slopes/RRFs and R2 for high and low ranges, and a zoomed-in plot for the low range for each monitored compound.

**Figure 6:**
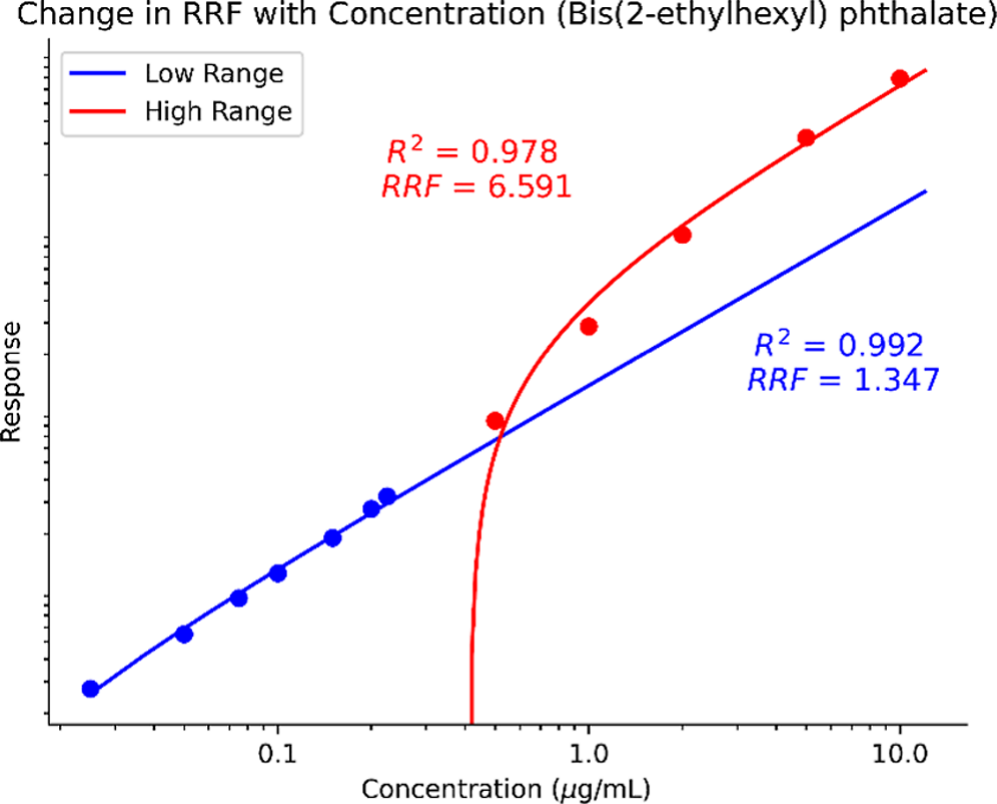
Log–log plot of bis­(2-ethylhexyl) phthalate across a wide range of concentrations using SVOC EI-GCMS. Two different dynamic ranges—a lower and higher concentration—show the RRFs for an individual compound are different as a function of concentration.

**Table 1:** Highlights of RRF Determined by Both Linear Regressions and Single Point Measurements of Monitored Compounds across a Wide Concentration Range

technique	chemical	RRF EW range	RRF at high range	RRF at max conc
LCMS(+)	triethyl citrate 77-93-0	1.348	0.235	0.16
NVOC	oleamide 301-02-0	0.748	0.263	0.16
LCMS(−)	TDS 4754-44-3	4.061	0.992	0.54
NVOC	dodecanoic acid 143-07-7	0.677	0.626	0.3
EI-GCMS SVOC	DEHP 117-81-7	1.347	6.591	1.73
	DBP 84-74-2	3.209	6.171	1.30
	indeno[1,2,3-cd]pyrene 193-39-5	1.281	5.797	1.34

These calculated RRFs show a complicated picture of the chemical performance across a wide range of concentrations. For LCMS­(+) and LCMS(−), the RRF at the maximum is significantly below the RRF of each range, which suggests that a single RRF measured at the maximum concentration would not be representative of compounds that are measured in normal chemical characterization calibrated concentration ranges. This is likely due to droplet saturation and competitive ionization effects reducing ion efficiencies.^31,32^ The SVOC compounds show a more complicated picture, especially with the assumption the EI-GCMS is a better behaving system.^3^ For diethylhexyl phthalate (DEHP), the RRF at the maximum is lower than the slope of the higher concentration range and then higher than the determined RRF for the normal Edwards Lifesciences dynamic range. For indeno­[1,2,3-*cd*]­pyrene CAS#193-39-5, the RRFs at the maximum range are roughly equivalent to the normal range, although different from the slope at the upper range. Likely, there is system overload at the elevated concentrations, which would then bias the UF if the RRFs and subsequent RSD are artificially compressed. Therefore, we recommend RRFs be determined not only within the dynamic range but as a slope of the qualified[Fn fn1] dynamic range. RRFs for all monitored compounds are listed in Supporting Information SI6.

The next question about determining RRFs is how they vary as part of day-to-day operations. Coeluting ions, contaminants, carryover, mobile phase issues, changes in tuning and source conditions, dirty ion optics reducing ion transmission, and so forth can all affect the observed response of a compound. These factors can be known or unknown, which can unequally affect individual compound responses, i.e., the effects on RRFs may be unequal across the chemical space. Isotopically labeled internal standards can mitigate some of these effects; however, it is unlikely to have an isotopically labeled compound for every extractable, so one or multiple ISTDs are employed, which should represent the extractables. These are frequently isotopically labeled reference/surrogate compounds, which are also subject to the same quantitation modeling and isotopic distribution biases. Figures 8–11 are violin plots showing the population distributions of RRFs; the LCMS(±)-monitored compound data was collected over 100 different instrumental calibrations, and the GCMS data was approximately 50 instrumental calibrations on different dates ranging from 2023 to 2025. The violin plots draw the kernel density estimate with an embedded box and whisker plot. The data used to generate these plots is in Supporting Information SI6.

**Figure 7:**
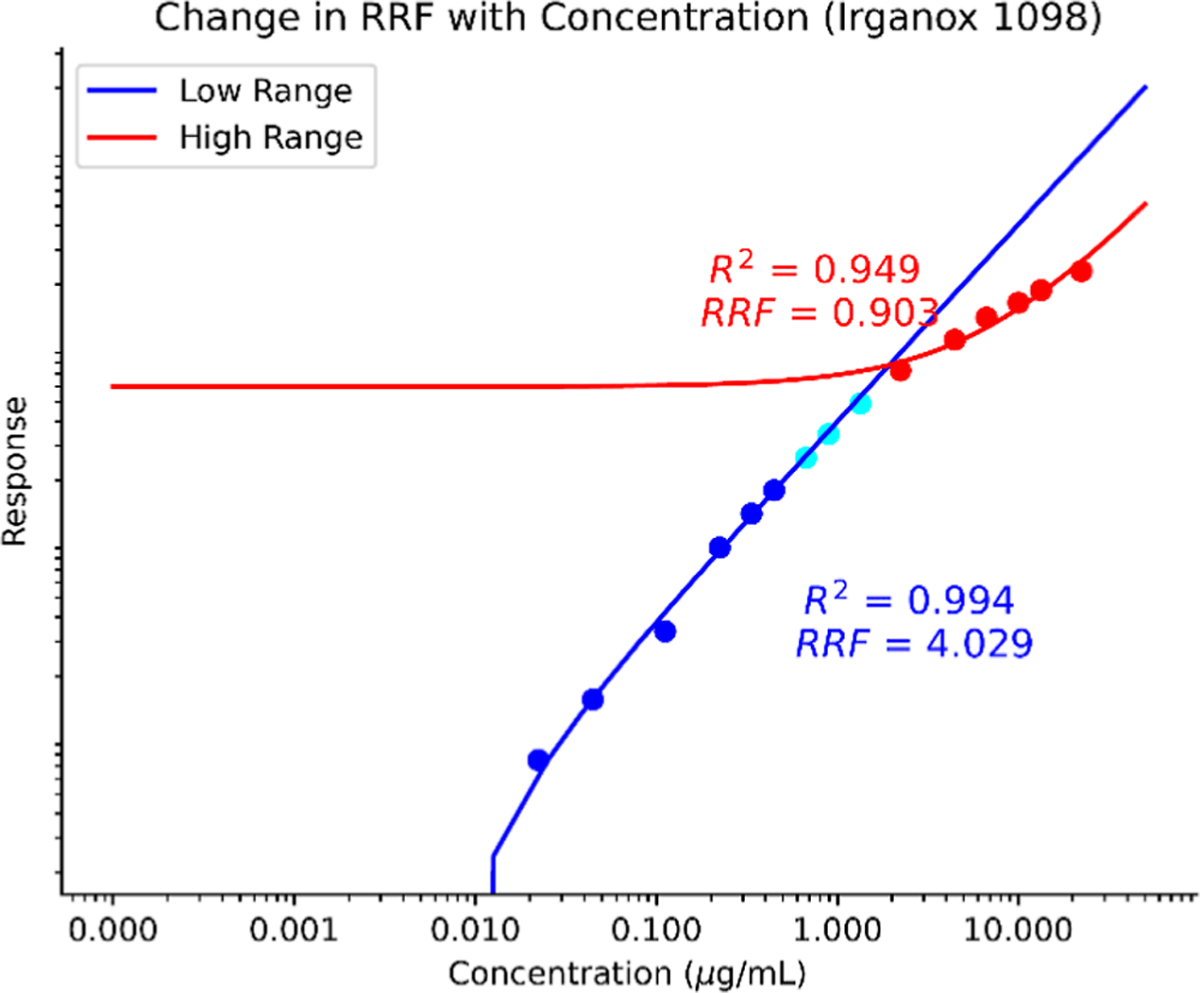
Log–log plot of Irganox 1098 across a wide range of concentrations using NVOC ESI-LC-MS. The RRFs across the concentrations are more pronounced in many NVOC-related compounds.

**Figure 8:**
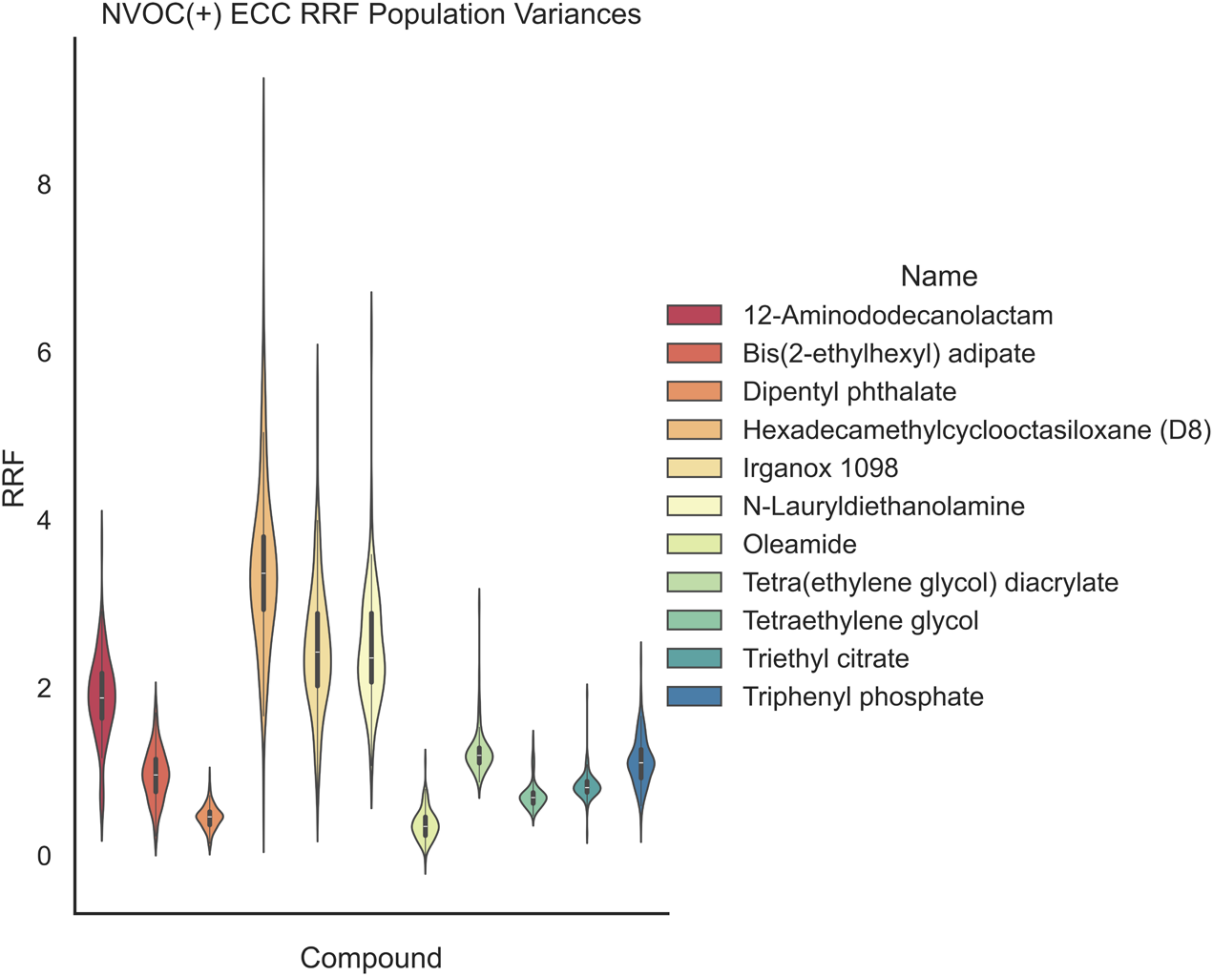
LCMS (+) ECC NVOC RRF population distributions. The variance of each compound can be large; single point measurements of RRFs ignore the normal population statistical measurements.

**Figure 9:**
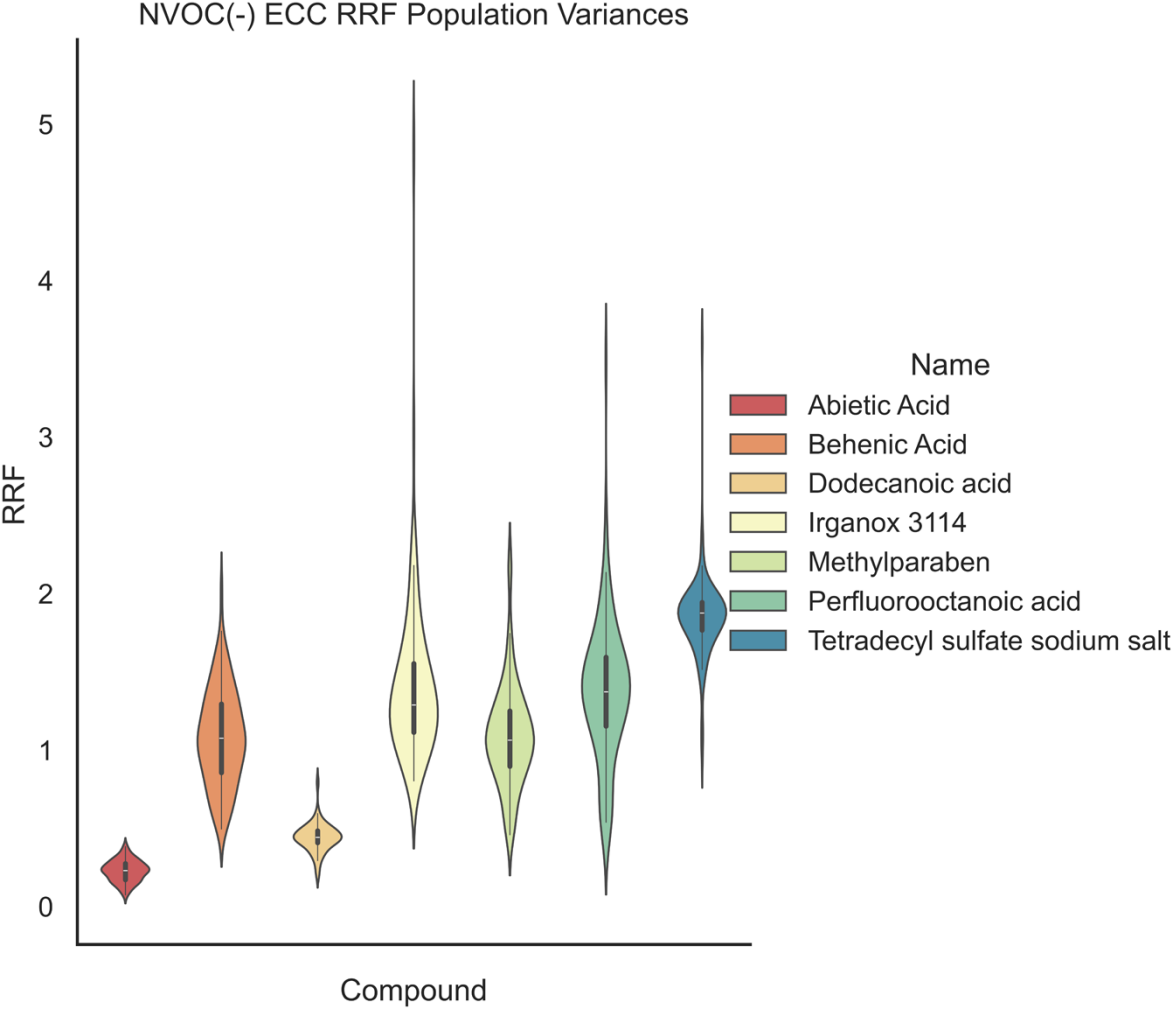
LCMS­(−) ECC NVOC RRF population distributions show an unequal variance, even within similar chemical groups.

**Figure 10:**
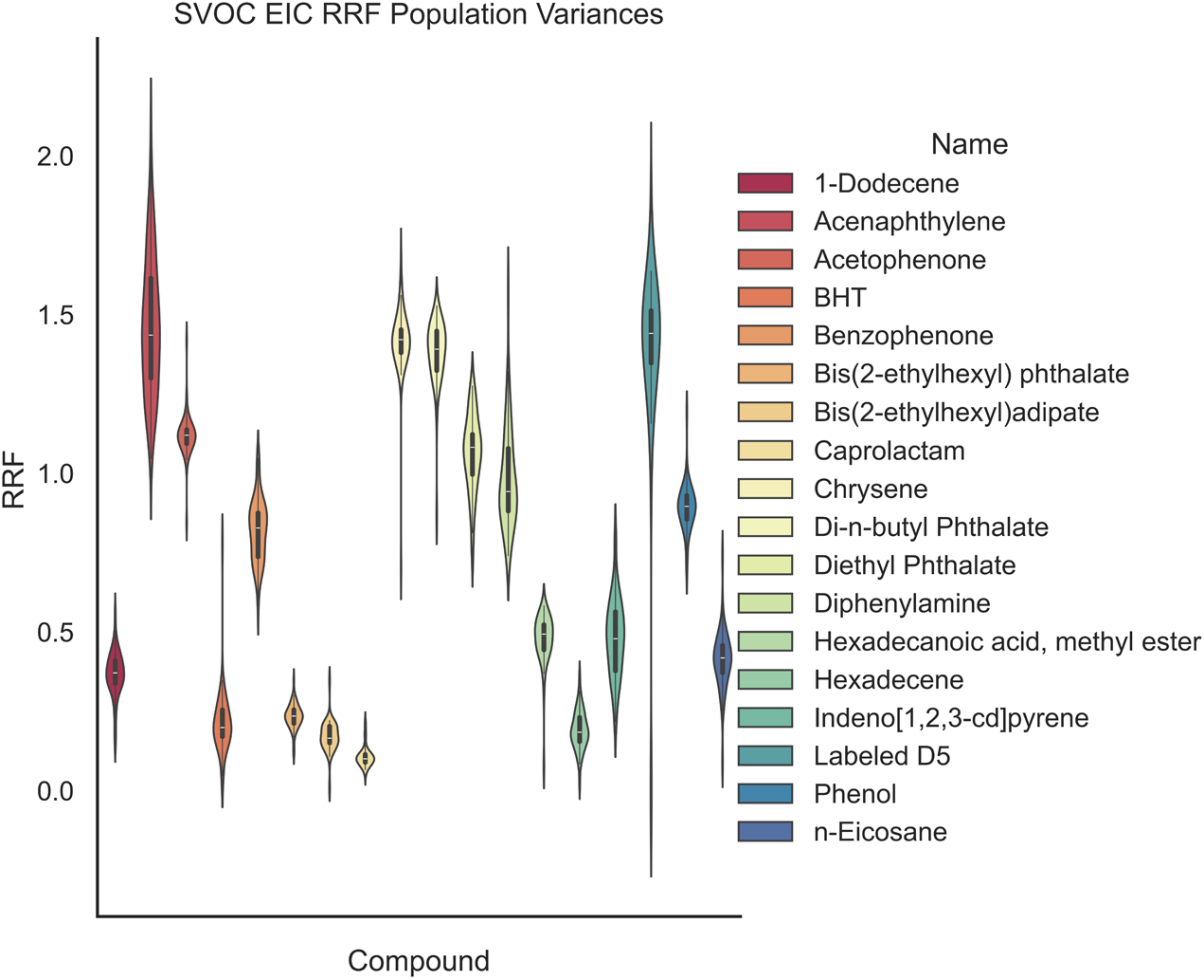
EI-GCMS EIC SVOC population distributions show a wide range of RRFs as a function of both chemistry and quantitation model biases.

**Figure 11:**
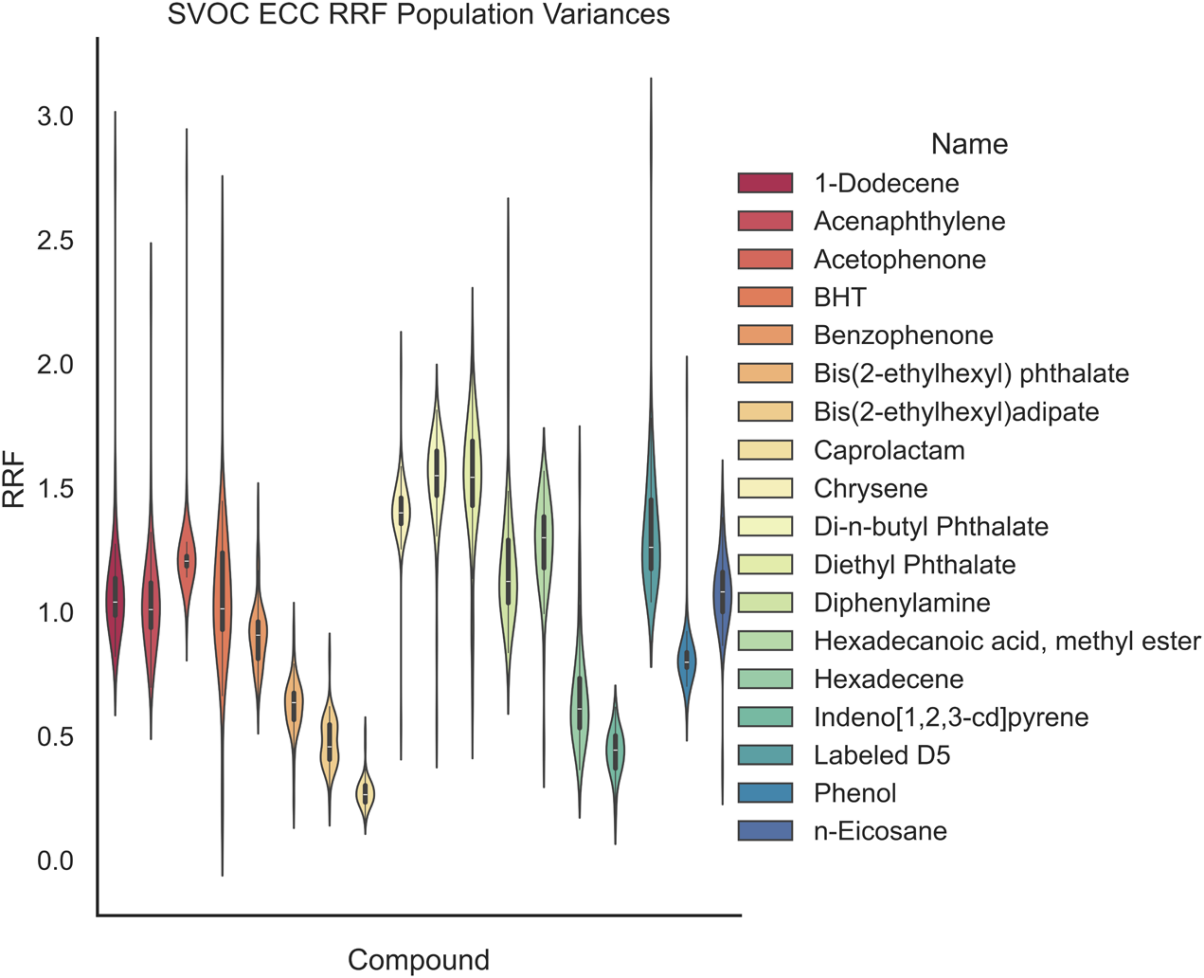
EI-GCMS ECC SVOC violin plots show a narrower variance across the entire range of chemicals; however, members of the similar class have significantly different populations.

In Figures 8 and 9, the LCMS(±) data show the population distribution of each monitored chemical. The population statistics for each compound are different even for chemicals from the same chemical class. Behenic and dodecanoic acid have significantly different population variances, the structrual difference is only a longer alkyl chain (LCMS(−) ECC RRF Welch *t*-test two tailed, *df* = 104, *p* = 3.42 × 10^–52^, α = 0.05). In Figures 10 and 11, the GCMS-monitored compounds are displayed in terms of both EIC and ECC population distributions. These plots show a similar story with DEHP and DBP, while similar chemical classes, their population means, and variances are different (GCMS ECC RRF Welch *t*-test two tailed, *df* = 47, *p* = 6.67 × 10^–31^, α = 0.05).

In this work, we show how the quantitation model can distort RRFs and how these distortions can manifest themselves over time. The overall variance of RRFs with ECC SVOC GCMS compounds is lower than the EIC model as shown in Figures 11 and 10, respectively. Reducing the quantitation model biases can result in a reduction of the UF.

Furthermore, population statistics are important for understanding RRFs. For chemical characterization publications^9,10^ which report the RRF of a compound once at a high concentration, a single measurement does not allow the laboratory to determine if this measured RRF is an outlier, nor understand the relationship of a chemical’s concentration with its RRF. If this RRF is used in quantitation, it may not lead to a calculated protective concentration—without knowing the population statistics, it is impossible to determine where the measured RRF lies in the protective or lower half of the population range. Because these RRFs may vary from run to run and instrument to instrument, the Edwards Lifesciences approach is to calculate the RRFs of reference and surrogate standards that describe the chemical space of medical devices on the day of analysis and not as a static number in perpetuity. Variability in determining RRFs exists and can often be unknowingly affecting the analysis. Thus, the RRF, and subsequently UF, of reference and surrogate standards that confidently describe the chemical space of the medical devices should be evaluated per instrument startup to accommodate all sources of variability: from unknown contaminants to the nature of competitive ionization to the quirks of specific instruments. These RRFs are applied only to the data set in which the calibrations were collected. The day-of-analysis RRFs represent the ability of the analytical methodology and instrument health to monitor and accommodate the compounds that represent potential extractables.

A notable advantage of day-of-analysis RRF determination is the enhanced flexibility it provides in the selection and application of internal standards. During chemical characterization analysis, where device-specific matrices can introduce unpredictable interferences, preassigned internal standards may not be universally applicable. The day-of-analysis approach allows analysts to select internal standards that are free from such interference and better align with the chemical constituents of the sample. This adaptability is particularly valuable when employing multiple internal standards to account for the chemical diversity of extractables provided that each is applied consistently throughout the analysis.

In contrast, external-data-driven approaches impose a significant constraint: the ISTD used during database construction must be identically applied during subsequent analyses. This requirement limits the ability to tailor ISTD selection to the specific matrix under investigation and may compromise quantitation accuracy when the database-assigned ISTD is affected by matrix-specific interferences. Thus, the day-of-analysis strategy offers a more robust and context-sensitive framework for ISTD application, supporting improved reproducibility and analytical confidence in semiquantitative chemical characterization and the resulting toxicological risk assessments.

Through our work, we interpret ISO10993-18 and the FDA draft guidance to be the following: selection of analytical method parameters and standards in chemical characterization is designed to be fit-for-purpose, accommodating the analytical needs specific to each device. We emphasize that RRFs observed at the time of testing and quantitation model show fit-for-use of these analytical method parameters, standards, and instrument/method performance for chemical characterization. Subsequently, RRFs and UFs are established during the analysis and applied only to that analysis, demonstrating both fit-for-use and fit-for-purpose.

Applying an UF to the analytical evaluation threshold concentration, as compared to a peak response_mean_ or RRF_84thpercentile_ ^16^—to define thresholds for identification and semiquantitation provides a more conservative and consistent starting point. This approach is consistent with Figure 1 for calculations to determine AET, where the units are μg/mL. This approach ensures that the same quantitation model is applied to both the standards and the extractables, enhancing the reliability and comparability of the results.

## Conclusions

RRFs play a critical role in the ISO10993-18 chemical characterization of extractables, both in determination of AET and semiquantitation. Recent literature^15^ has recommended using single measurements of standards to determine RRFs, enabling these one-time measurements to be used in chemical characterization. This approach ignores the reality that RRFs are not static because of a variety of factors, from tuning to unknown contaminants. There is attention given to the nature of “protective” concentrations, the idea that the quantitation based on the RRF of compounds should not underestimate the quantities of nontarget extractables. Throughout this work, we show that many different decisions can alter and bias the RRFs. This highlights the need for any approach or strategy to define its quantitation model and to understand the population statistics. If an approach does not resolve these issues, then this approach or strategy may not be protective. Using the external database built from RRFs measured once at high concentrations without description of the entries in the database nor its quantitation model, one cannot show a “protective” strategy without understanding the population statistics of the database in contrast to Jenke.^33^ In this work, the external database is then used to adjust the AET as well as to modify the reported concentrations. If RRFs are measured once and applied to analyses later, maybe years later, it is difficult to be confident whether the single measurement is an outlier. This can lead to an underreporting of concentration, which means compounds may not reach the threshold to be evaluated for patient safety, e.g., below the AET. The quantitation model directly affects the evaluation of biocompatibility of medical devices, and it should be well described in all instances.

The single measurements of RRFs, often at high concentrations, ignore the reality of the population statistics of mass spectrometric detection. We recommend that in the field of chemical characterization for medical device biocompatibility, this variance is accounted for by day-of measurements of RRFs and subsequently UF and AETs. Therefore, as corroborated by this research work, RRFs should be determined as follows: (a) every instrumental calibration during a nontarget chemical characterization study to account for known and unknown variability during analysis, (b) from a set of surrogates that represent the potential extractables, (c) surrogate compound RRFs should be determined from the slope of the dynamic range and only applied to the data collected post instrument calibration and within the dynamic range, and (d) at least one appropriate internal standard—preferably isotopically labeled version of a surrogate—should be used to account for instrumental and matrix effect variances. Our research shows that the ECC quantitation model is preferred over the EIC quantitation model, and where the EIC model is deemed appropriate, a multi-ion EIC monoisotopic ion for each adduct should be preferred over handpicking a monoisotopic ion of a single adduct EIC approach. This quantitation model should be consistent throughout the analysis for calculating RRFs, UFs, and AET determination and subsequently semiquantitation of NTEs.

Returning to the guidance recommendations of utilizing a database of 200–500 compounds,^5,15^ we have shown the need for frequent measurement of RRFs. A single measurement of an RRF for an external database does not account for variability; RRFs need to be constantly monitored for interferences. External databases assume no instrumental or quantitation modeling biases on compound concentration, which questions the presumption of a “protective concentration”. While these external databases are valuable for proving chemical space coverage for analytical methods and confirmation for identifications, it is inappropriate to utilize an external database model, as it does not match the reality of these mass spectrometers. Variability exists in these measurements, and external databases do not provide fit-for-purpose and fit-for-use.

## Supplementary Material













## Footnotes

a Refer to ISO10993-18:2020 for description on qualifying an analytical method for chemical characterization.

## Notes

Supporting Information Cover letter and table of contents of SI (PDF)
Example COAs for monitored chemicals in this study (ZIP)
HRAM-LCMS spectra showing additional situations where EIC underrepresents RRFs compared to the total ionization (PDF)
Spectra from dilaurylthiodipropionate 123-28-4 collected at various instances showing changing relative ratios of adducts (PDF)
All calibration plots—normal and log–log axes—for all SVOC- and NVOC-monitored compounds (ZIP)
Tabular data for the extended dynamic range plots and RRF for violin plots values (XLSX)

## Abbreviations

ISO: International Organization for Standards
UF: uncertainty factor
FDA: Food and Drug Administration
AET: analytical evaluation threshold
RSD: relative standard deviation
RRF: relative response factor
EI: electron ionization
GCMS: gas chromatography mass spectrometry
LCMS: liquid chromatography mass spectrometry
CAD: charged aerosol detector
UV/vis: ultraviolet visible detection
FID: flame ionization detector
ESI: electrospray ionization
APCI: atmospheric pressure chemical ionization
HRAM: high-resolution accurate mass
CLAP: chemicals list for analytical performance
RST: regulatory science tool
TIC: total ion chromatogram
EIC(s): extracted ion chromatogram(s)
NTEs: non-target extractables
HES: high efficiency source
SVOC: semivolatile organic compounds
MSD: mass selective detector
MS1: single-stage mass spectrometry
MS2: Tandem mass spectrometry
*m*/*z*: mass to charge ratio
AJS: Agilent jet stream
*R* ^2^: coefficient of determination
RT: retention time
SNR: signal to noise ratio
BPC: base peak chromatogram
ECC: extracted compound chromatogram
E&L: extractables and leachables
NIST AMDIS: National Institute of Standards and Technology Automated Mass Spectral Deconvolution and Identification System
BHT: butylated hydroxy toluene
DTDPP: dilauryl thiodipropionate
D8: hexadecamethylcyclooctasiloxane
ISTD: internal standard
DEHP: diethylhexyl phthalate

## References

[1] International Organization for Standardization . Biological Evaluation of Medical Devices—Process, Part 1: Evaluation and Testing within a Risk Management, 2018.

[2] Food and Drug Administration . General Considerations for Animal Studies Intended to Evaluate Medical Devices, 2023.

[3] International Organization for Standardization . Biological Evaluation of Medical Devices—Part 18: Chemical Characterization of Medical Device Materials within a Risk Management Process, 2020.

[4] International Organization for Standardization . Biological Evaluation of Medical Devices—Part 18: Chemical Characterization of Medical Device Materials within a Risk Management Process AMENDMENT 1: Determination of the Uncertainty Factor, 2022. https://www.iso.org/standard/64750.html#amendment.

[5] U.S. Food and Drug Administration . Center for Devices and Radiological Health. C. for B. E. and R. Chemical Analysis for Biocompatibility Assessment of Medical Devices; U.S. Food and Drug Administration, 2024. https://www.fda.gov/media/181952/download (accessed Sept 19, 24).

[6] Jenke D., Christiaens P., Beusen J.-M., Verlinde P., Baeten J. (2022). A Practical
Derivation of the Uncertainty Factor Applied to Adjust the Extractables/Leachables
Analytical Evaluation Threshold (AET) for Response Factor Variation. PDA J. Pharm. Sci. Technol..

[7] Jordi M., Heise T. (2021). An Analytical Strategy Based on Multiple
Complementary and Orthogonal
Chromatographic and Detection Methods (Multidetector Approach) to
Effectively Manage the Analytical Evaluation Threshold (AET). PDA J. Pharm. Sci. Technol..

[8] Zdravkovic S. A., Fu Q., Flick A., Jana B., Looney E., Kikandi S., Verdonck T., Nagao L., Bielinski M. K. (2025). Understanding
Alignment in the Execution of Extractable Screening Studies Between
Laboratories: Results of the ELSIE Lab Practices Sub-Team Industry
Surveys. PDA J. Pharm. Sci. Technol..

[9] Jenke D., Christiaens P., Verlinde P., Baeten J., Beusen J. M. (2024). Correlating
GC/MS Relative Response Factors to Analyte’s Physicochemical
and Chromatographic Properties to Facilitate the Quantitation of Organic
Extractables and Leachables in Non-Targeted Analysis (NTA). Concepts Empir. Consid..

[10] Zdravkovic S. A., Duong C. T., Hellenbrand A. A., Duff S. R., Dreger A. L. (2018). Establishment
of a Reference Standard Database for Use in the Qualitative and Semi-Quantitative
Analysis of Pharmaceutical Contact Materials within an Extractables
Survey by GC–MS. J. Pharm. Biomed. Anal..

[11] Norwood D., Michelson A., Dunn N., Duett J., Fleck L., Vas G. (2022). Impact of
the GC-MS Injection Solvent and the Analyte Concentration
on Relative Responses for Common Extractables Betasciencepress Publishing. Rev. Sep. Sci..

[12] Flohl M., Tolstyakova M., Seyyal E., Michelson A., Fleck L., Vas G. (2025). Practical Considerations of FDA’s
CLAP List to Support Testing of Extractables for Pharmaceuticals and
Medical Devices. ACS Omega.

[13] Yun B. H., Herath A., Jin Y., Kim J., Belton K., Rufer E., Rivera Betancourt O. (2025). Designing
a Set of Reference Standards
for Non-Targeted Analysis of Polymer Additives Extracted from Medical
Devices. J. Expo. Sci. Environ. Epidemiol..

[14] U.S. Food and Drug Administration . Chemicals List for Analytical Performance (CLAP), 2024. RST24MC05.01.

[15] Jenke D., Christiaens P., Verlinde P., Baeten J., Beusen J.-M. (2024). Correlating
GC/MS Relative Response Factors to Analyte′s Physicochemical
and Chromatographic Properties to Facilitate the Quantitation of Organic
Extractables and Leachables in Non-Targeted Analysis (NTA) I. Concepts
and Empirical Considerations. PDA J. Pharm.
Sci. Technol..

[16] Jenke D., Christiaens P., Heise T. (2024). Identification and Quantification
of Medical Device Extractables and Leachables via Non-Target Analysis
(NTA); Analytical Uncertainty. J. Pharm. Biomed.
Anal..

[17] Christiaens P., Beusen J. M., Verlinde P., Baeten J., Jenke D. (2020). Identifying
and Mitigating Errors in Screening for Organic Extractables and Leachables:
Part 2-Errors of Inexact Identification and Inaccurate Quantitation. PDA J. Pharm. Sci. Technol..

[18] Jenke D., Heise T. (2021). The Implications of Chromatographically
Screening Medical Products
for Organic Leachables Down to the Analytical Evaluation Threshold
Adjusted for Response Factor Variation. PDA
J. Pharm. Sci. Technol..

[19] Jordi M. A., Khera S., Roland K., Jiang L., Solomon P., Nelson J., Lateef S. S., Woods J., Martin L., Martin S., Aiello F., Chen N. (2018). Qualitative
Assessment
of Extractables from Single-Use Components and the Impact of Reference
Standard Selection. J. Pharm. Biomed. Anal..

[20] Jordi M. A., Rowland K., Liu W., Cao X., Zong J., Ren Y., Liang Z., Zhou X., Louis M., Lerner K. (2020). Reducing Relative
Response Factor Variation Using a Multidetector Approach for Extractables
and Leachables (E&L) Analysis to Mitigate the Need for Uncertainty
Factors. J. Pharm. Biomed. Anal..

[21] Sussman E. M., Oktem B., Isayeva I. S., Liu J., Wickramasekara S., Chandrasekar V., Nahan K., Shin H. Y., Zheng J. (2022). Chemical Characterization
and Non-Targeted Analysis of Medical Device Extracts: A Review of
Current Approaches, Gaps, and Emerging Practices. ACS Biomater. Sci. Eng..

[22] Singh G., Lu D., Liu C., Hower D. (2021). Analytical Challenges and Recent
Advances in the Identification and Quantitation of Extractables and
Leachables in Pharmaceutical and Medical Products. TrAC, Trends Anal. Chem..

[23] Charest N., Pu S., Mccord J. P., Williams A. J., Sobus J. R. (2025). Examining Structure—Based
Surrogate Selection for Quantitative Non—Targeted Analysis. Anal. Bioanal. Chem..

[24] Sica V. P., Krivos K. L., Kiehl D. E., Pulliam C. J., Henry I. D., Baker T. R. (2020). The Role of Mass Spectrometry and Related Techniques
in the Analysis of Extractable and Leachable Chemicals. Mass Spectrom. Rev..

[25] Tsugawa H., Cajka T., Kind T., Ma Y., Higgins B., Ikeda K., Kanazawa M., VanderGheynst J., Fiehn O., Arita M. (2015). MS-DIAL: data-independent MS/MS deconvolution
for comprehensive metabolome analysis. Nat.
Methods.

[26] Giera M., Aisporna A., Uritboonthai W., Hoang L., Derks R. J. E., Joseph K. M., Baker E. S., Siuzdak G. (2024). XCMS-METLIN: Data-Driven
Metabolite, Lipid, and Chemical Analysis. Mol.
Syst. Biol..

[27] Schmid R., Heuckeroth S., Korf A., Smirnov A., Myers O., Dyrlund T. S., Bushuiev R., Murray K. J., Hoffmann N., Lu M., Sarvepalli A., Zhang Z., Fleischauer M., Dührkop K., Wesner M., Hoogstra S. J., Rudt E., Mokshyna O. (2023). Integrative Analysis of Multimodal Mass Spectrometry
Data in MZmine 3. Nat. Biotechnol..

[28] Chambers (2012). A Cross-Platform Toolkit
for Mass Spectrometry and Proteomics. Nat. Biotechnol..

[29] Bishop L. M., Shen T., Fiehn O. (2023). Improving Quantitative Accuracy in
Nontargeted Lipidomics by Evaluating Adduct Formation. Anal. Chem..

[30] Technology, N. I. of S. and. Automated Mass Spectral Deconvolution and Identification System, 2025. http://www.amdis.net/index.html (accessed Nov 6, 2025).

[31] Page J. S., Kelly R. T., Tang K., Smith R. D. (2007). Ionization and transmission
efficiency in an electrospray ionization—mass spectrometry
interface. J. Am. Soc. Mass Spectrom..

[32] Liigand P., Liigand J., Kaupmees K., Kruve A. (2021). Analytica Chimica Acta
30 Years of Research on ESI/MS Response: Trends, Contradictions and
Applications. Anal. Chim. Acta.

[33] Jenke D. (2024). Accurate or
Protective: What Is the Goal of Non-Targeted Extractables and Leachables
Quantitation and Identification?. PDA J. Pharm.
Sci. Technol..

