# Supplementary material for: Challenging the Database: Day-of-Analysis Calibration and UF Modeling for Reliable RRF Use in Medical Device Chemical Characterization
Source: Anal Chem. 2025 Oct 8;97(41):22719–29. doi: 10.1021/acs.analchem.5c04247 (PMC12547855; doi:10.1021/acs.analchem.5c04247)
Supplement: Supplementary file 1 [file ac5c04247_si_001.pdf]

Challenging the Database: Day-of-Analysis Calibration and UF Modeling for Reliable RRF Use in Medical Device Chemical Characterization

Michael Rush[a], J. David Ricker[a], Pramod Prasad Poudel[a], Nuwan Kothalawala[a], Cary Watterson[a], Dmitriy Pastarnak[a]*

[a]Edwards Lifesciences, 12050 Lone Peak Parkway, Draper, Utah, 84020 USA

*email: Michael_rush@edwards.com

Table of Contents

SI1: Supplementary Information Title Page and Table of Contents

SI2: Example COAs for monitored chemicals in this study (.zip)

SI3: HRAM LCMS Spectra (.TIF)

SI4: Spectra from Dilaurylthiodipropionate 123-28-4 collected at various instances showing changing relative ratios of adducts (.TIF)

SI5: All calibration plots – normal and log log axes – for all SVOC and NVOC monitored compounds (.zip)

SI6: Tabular data for the extended dynamic range plots and RRF for violin plots values
