# Supplementary material for: Challenging the Database: Day-of-Analysis Calibration and UF Modeling for Reliable RRF Use in Medical Device Chemical Characterization
Source: Anal Chem. 2025 Oct 8;97(41):22719–29. doi: 10.1021/acs.analchem.5c04247 (PMC12547855; doi:10.1021/acs.analchem.5c04247)
Supplement: Supplementary file 2 [file ac5c04247_si_002.zip › 171468-BULK_______WXBD6815V_.pdf]

3050 Spruce Street, Saint Louis, MO 63103, USA
Website: www.sigmaaldrich.com
Email USA: techserv@sial.com
Outside USA: eurtechserv@sial.com

Certificate of Analysis

Product Name:
Perfluorooctanoic acid - 95%

**Product Number:**	**171468**	
**Batch Number:**	**WXBD6815V**	
Brand:	ALDRICH	
CAS Number:	335-67-1	
Formula:	C8HF15O2	
Formula Weight:	414,07 g/mol	
Quality Release Date:	26 NOV 2021	

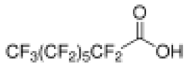

Test	Specification	Result
Appearance (Colour)	White to Off-White	White
Appearance (Form)<br>Powder, Crystals, Crystalline Powder and/or Flakes	Conforms to Requirements	Solid
Infrared Spectrum	Conforms to Structure	Conforms
Purity (Titration by NAOH)	95.5 - 104.5 %	101.2 %
Water (by Karl Fischer)	≤ 4.5 %	0.1 %
GC (area %)	≥ 95.5 %	99.8 %

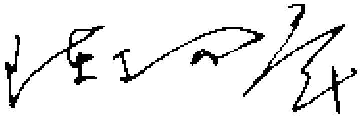

Steven Chen
Quality Manager
Wuxi, China CN

Sigma-Aldrich warrants, that at the time of the quality release or subsequent retest date this product conformed to the information contained in this publication. The current Specification sheet may be available at Sigma-Aldrich.com. For further inquiries, please contact Technical Service. Purchaser must determine the suitability of the product for its particular use. See reverse side of invoice or packing slip for additional terms and conditions of sale.
