# Supplementary material for: Challenging the Database: Day-of-Analysis Calibration and UF Modeling for Reliable RRF Use in Medical Device Chemical Characterization
Source: Anal Chem. 2025 Oct 8;97(41):22719–29. doi: 10.1021/acs.analchem.5c04247 (PMC12547855; doi:10.1021/acs.analchem.5c04247)
Supplement: Supplementary file 2 [file ac5c04247_si_002.zip › 216941-BULK_______MKCM6914__.pdf]

3050 Spruce Street, Saint Louis, MO 63103, USA
Website: www.sigmaaldrich.com
Email USA: techserv@sial.com
Outside USA: eurtechserv@sial.com

Certificate of Analysis

Product Name:
Behenic acid – 99%

Product Number: **216941**
Batch Number: **MKCM6914**
Brand: ALDRICH
CAS Number: 112-85-6
MDL Number: MFCD00002807
Formula: C22H44O2
Formula Weight: 340.58 g/mol
Quality Release Date: 30 JUN 2020

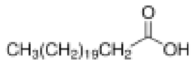

Test	Specification	Result
Appearance (Color)	White	White
Appearance (Form)	Conforms to Requirements	Flakes
Flakes, Powder, Beads or Chunks		
Infrared Spectrum	Conforms to Structure	Conforms
Carbon	76.4 - 78.7 %	77.5 %
GC (area %)	$\geq$ 98.5 %	99.4 %

Michael Grady, Manager
Quality Control
Milwaukee, WI US

Sigma-Aldrich warrants, that at the time of the quality release or subsequent retest date this product conformed to the information contained in this publication. The current Specification sheet may be available at Sigma-Aldrich.com. For further inquiries, please contact Technical Service. Purchaser must determine the suitability of the product for its particular use. See reverse side of invoice or packing slip for additional terms and conditions of sale.
