# Supplementary material for: Challenging the Database: Day-of-Analysis Calibration and UF Modeling for Reliable RRF Use in Medical Device Chemical Characterization
Source: Anal Chem. 2025 Oct 8;97(41):22719–29. doi: 10.1021/acs.analchem.5c04247 (PMC12547855; doi:10.1021/acs.analchem.5c04247)
Supplement: Supplementary file 2 [file ac5c04247_si_002.zip › 283843-BULK_______MBBC7706__.pdf]

SIGMA-ALDRICH®

sigma-aldrich.com

3050 Spruce Street, Saint Louis, MO 63103, USA
Website: www.sigmaaldrich.com
Email USA: techserv@sial.com
Outside USA: eurtechserv@sial.com

Certificate of Analysis

Product Name:
Triethyl phosphonoacetate-$^{13}C_2$ - 99 atom % ^{13}C

**Product Number:**	**283843**
**Batch Number:**	**MBBC7706**
Brand:	ALDRICH
CAS Number:	100940-60-1
MDL Number:	MFCD00064452
Formula:	13C2C6H17O5P
Formula Weight:	226.18 g/mol
Quality Release Date:	30 AUG 2019

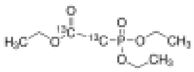

Test	Specification	Result
Appearance (Clarity)	Clear	Clear
Appearance (Color)	Colorless	Colorless
Appearance (Form)	Liquid	Liquid
Purity (GC)	$\geq$ 99 %	99 %
Proton NMR Spectrum	Conforms	Conforms
13C NMR Spectrum	Conforms	Conforms
Proton NMR	$\geq$ 99 %	99 %
Water (by Karl Fischer)	$\leq$ 0.5 %	0.4 %

Laura E. Baird, Manager
Quality Assurance & Control
Miamisburg, Ohio US

Sigma-Aldrich warrants, that at the time of the quality release or subsequent retest date this product conformed to the information contained in this publication. The current Specification sheet may be available at Sigma-Aldrich.com. For further inquiries, please contact Technical Service. Purchaser must determine the suitability of the product for its particular use. See reverse side of invoice or packing slip for additional terms and conditions of sale.
