# Supplementary material for: Challenging the Database: Day-of-Analysis Calibration and UF Modeling for Reliable RRF Use in Medical Device Chemical Characterization
Source: Anal Chem. 2025 Oct 8;97(41):22719–29. doi: 10.1021/acs.analchem.5c04247 (PMC12547855; doi:10.1021/acs.analchem.5c04247)
Supplement: Supplementary file 2 [file ac5c04247_si_002.zip › 1545034-R181Y0.pdf]

Certificate

PLASTIC ADDITIVE 12

(Oleamide)

USP Catalog No.:	1545034
USP Lot No.:	R181Y0

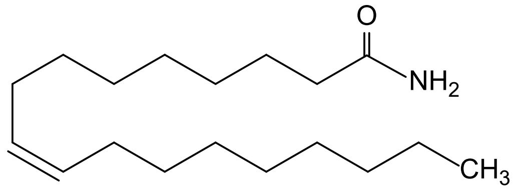

CAS Number:	301-02-0
Molecular Formula:	$C_{18}H_{35}NO$
Molecular Weight:	281.48

Copyright 2018 The United States Pharmacopeial Convention. All rights reserved.

LABEL TEXT

For use with specified USP compendial tests. Not for use as a drug. See SDS prior to use at www.usp.org/sds.

Lot: R181Y0

REFERENCE STANDARD

PLASTIC ADDITIVE 12 100 mg

Warning! Causes eye irritation.

For quantitative applications, use a value of 1.00 mg of plastic additive 12 per mg of material on the as is basis.
Store in a freezer. Keep container tightly closed.

See certificate for any additional information.
USP, 12601 Twinbrook Pkwy, Rockville, MD, +1-301-881-0666
Cat. No. 1545034 Material mfd. in United States

Wash thoroughly after handling. If in eyes: Rinse cautiously with water for several minutes. Remove contact lenses, if present and easy to do. Continue rinsing. If eye irritation persists: Get medical advice/attention.

Danielle A. Vattimo

Quality Assurance

Certificate Version History

Version Number	Date	Reasons for Change
00 (Current)	**20-APR-2023**	First issue

Copyright 2018 The United States Pharmacopeial Convention. All rights reserved.

Assigned Value
Please refer to the USP Reference Standard label and/or USP Certificate for the assigned value of the specific lot. Ifan assigned value is not included on the label or Certificate, the lot was developed for qualitative USP compendial use and an assigned value will not be provided.

Valid Use Date
It is the responsibility of the user to ascertain that a particular lot of a USP Reference Standard has official status either as a "Current Lot" or as a "Previous Lot" within the assigned valid use date. The online USP Reference Standards Catalog and the online USP Store at www.usp.org are updated daily. USP recommends referring to one of these sources prior to using a USP RS to make sure the lot is valid for use.

Storage
Storage conditions are lot–specific and may change from one lot to another. The storage condition for an unopened USP Reference Standard is provided on the container label only, not on the Safety Data Sheet. If no specific directions or limitations are on the label, conditions of storage include storage at room temperature and protection from moisture, light, freezing, and excessive heat. See General Chapter <659> in the USP-NF Online for storage and handling definitions.

Instructions for Use
Follow the instructions provided on this Certificate, on the label of the USP Reference Standard, and in theassociated USP documentary standard(s). Please refer to General Chapter <11> for additional information.

Non-USP Compendial Use
USP Reference Standards are intended only for use in analytical or laboratory applications generally as specified in USP compendia. They are not for use in humans or animals as drugs or medical devices. It may be possible to use a USP RS outside of its associated USP compendial applications; however, it is the responsibility of the user to determine the suitability of the USP RS for a non-USP use.

LEGAL NOTICE
USP WARRANTS GOOD TITLE TO USP REFERENCE STANDARDS ON DISPATCH FROM USP. THE FOREGOING WARRANTY IS IN LIEU OF ANY OTHER WARRANTIES, EXPRESS OR IMPLIED, INCLUDING WITHOUT LIMITATION ANY WARRANTY OF MERCHANTABILITY OR FITNESS FOR A PARTICULAR PURPOSE, OR ANY WARRANTY THAT THE PRODUCTS, INCLUDING THIS CERTIFICATE, ARE OF MERCHANTABLE QUALITY. USP'S LIABILITY ARISING OUT OF OR RELATING TO THE SUPPLY OF USP REFERENCE STANDARDS AND THIS CERTIFICATE SHALL IN NO EVENT INCLUDE LOSS OF PROFITS, COST OF PROCURING SUBSTITUTE GOODS OR SERVICES, OR ANY INCIDENTAL, INDIRECT, OR CONSEQUENTIAL DAMAGES OF ANY KIND, EVEN IF USP IS AWARE OF THE POSSIBILITY OF SUCH DAMAGES. WITHOUT LIMITING THE GENERALITY OF THE FOREGOING, USP DOES NOT WARRANT THAT THE USE OR RESALE OF USP REFERENCE STANDARDS, INCLUDING THEIR USE TO PERFORM TESTS AND ASSAYS PUBLISHED BY USP, WILL NOT INFRINGE UNITED STATES OR ANY OTHER PATENTS.

USP Reference Standards are not intended for use as drugs, dietary supplements, or as medical devices.

This certificate may not be reproduced without the express written permission of USP.

Copyright 2018 The United States Pharmacopeial Convention. All rights reserved.
