# Supplementary material for: Challenging the Database: Day-of-Analysis Calibration and UF Modeling for Reliable RRF Use in Medical Device Chemical Characterization
Source: Anal Chem. 2025 Oct 8;97(41):22719–29. doi: 10.1021/acs.analchem.5c04247 (PMC12547855; doi:10.1021/acs.analchem.5c04247)
Supplement: Supplementary file 2 [file ac5c04247_si_002.zip › COA Sigma Methylparaben PHR1012 LRAD2978.pdf]

Certificate of Analysis – Certified Reference Material

METHYL PARAHYDROXYBENZOATE

(Methylparaben)

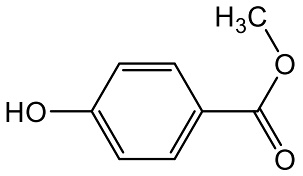

Product no.: PHR1012-1G

Lot no.: LRAD2978

Description of CRM: White Powder

Expiry date: 30 November 2026

Storage: Room Temperature

Certificate version: LRAD2978.01 *(Note: Certificates may be updated due to Pharmacopeial Lot Changes or the availability of new data. Check our website at: www.sigma-aldrich.com for the most current version.)*

Chemical formula: $C_8H_8O_3$

Molecular mass: 152.15

CAS No.: 99-76-3

Analyte	Certified Purity ± associated uncertainty *U, U=k · u (k=)* (Mass Balance/ basis)
**Methylparaben**	**99.9 % Ucrm = ± 0.2 %, k = 2.0 (as is basis)**

Metrological traceability: Traceable to the SI and higher order standards from NIST through an unbroken chain of comparisons. Additional traceability to Primary Standards is established through comparative assay determinations. See “Details on metrological traceability” on page 2.

Measurement method: Where applicable, the certified value is based on a purity determination by mass balance. See “Certification process details” on page 3.

Intended use: Intended for R&D and Analytical Use only. Not for drug, household or other uses.

Minimum sample size: 10 mg

Instructions for handling and correct use: Do not dry, use on the as is basis. The internal pressure of the container may be slightly different from the atmospheric pressure at the user’s location. Open slowly and carefully to avoid dispersion of the material. Attachment of a 20 mm aluminum crimp seal recommended for unused portions.

Health and safety information: All chemical reference materials should be considered potentially hazardous and should be used only by qualified laboratory personnel. Please refer to the Safety Data Sheet for detailed information about the nature of any hazard and appropriate precautions to be taken.

Accreditation: Sigma-Aldrich RTC is accredited by the US accreditation authority ANAB as a registered reference material producer AR-1470 in accordance with ISO 17034.

Certificate issue date: 25 October 2022

ANAB ACCREDITED ISO 17034 REFERENCE MATERIAL

ISO 17034
AR-1470

[Andy Ommen; Quality Control]

Shawn Stetler- QA Manager

Sigma-Aldrich RTC, 2931 Soldier Springs Rd. Laramie, WY 82070, USA;
Tel. 1 307-742-5452; Fax 1 307-855-831-9211; www.sigmaaldrich.com
Sigma-Aldrich RTC is a subsidiary of Merck KGaA, Darmstadt, Germany.

**Packaging:**	1 gram in amber vial
**Details on metrological traceability:**	This standard has been gravimetrically prepared using balances that have been fully qualified and calibrated to ISO 17025 requirements. All calibrations utilize NIST traceable weights which are calibrated externally by a qualified ISO 17025 accredited calibration laboratory to NIST standards. Qualification of each balance includes the assignment of a minimum weighing by a qualified and ISO 17025 accredited calibration vendor taking into consideration the balance and installed environmental conditions to ensure compliance with USP tolerances of NMT 0.10% relative error. Fill volume to predetermined specifications is gravimetrically verified throughout the dispensing process using qualified and calibrated balances. Further traceability to a corresponding Primary Standard may be achieved through a direct comparison assay. Where a Primary Standard is available, the assay value will be included in the specified section of the COA.
**Associated uncertainty:**	Uncertainty values in this document are expressed as Expanded Uncertainty (U_{CRM}) corresponding to the 95% confidence interval. U_{CRM} is derived from the combined standard uncertainty multiplied by the coverage factor *k*, which is obtained from a *t*-distribution and degrees of freedom. The components of combined standard uncertainty include the uncertainties due to characterization, homogeneity, long term stability, and short term stability (transport). The components due to stability are generally considered to be negligible unless otherwise indicated by stability studies.

Traceability Assay:
Comparative assay demonstrates direct traceability to Pharmacopeial Standards

ASSAY vs. USP REFERENCE STANDARD (1432005) (as is basis)

**ASSAY VALUE**	**vs. USP LOT**
**99.0 %**	**R098B0**<br>Labeled Content = 0.998 mg/mg

ASSAY vs. EP CRS (M1650000) (as is basis)

**ASSAY VALUE**	**vs. EP BATCH**
**99.3 %**	**5.0**<br>Labeled Content = 99.8 %

Method: HPLC (ref.: Methylparaben, Current Compendial Monographs)
Column: Ascentis Express C18, 150 mm x 4.6mm, 5µm particle size
Mobile Phase A: 6.8 g/L Potassium phosphate monobasic in water
Mobile Phase B: Methanol
Mobile Phase Ratio: 35:65(A: B)
Flow Rate: 0.8 mL/min
Column Temperature: 30 °C
Injection Volume: 10 µL
Detector: DAD, Wavelength: 272 nm

Representative Chromatogram from Lot: LRAD2978 Analysis

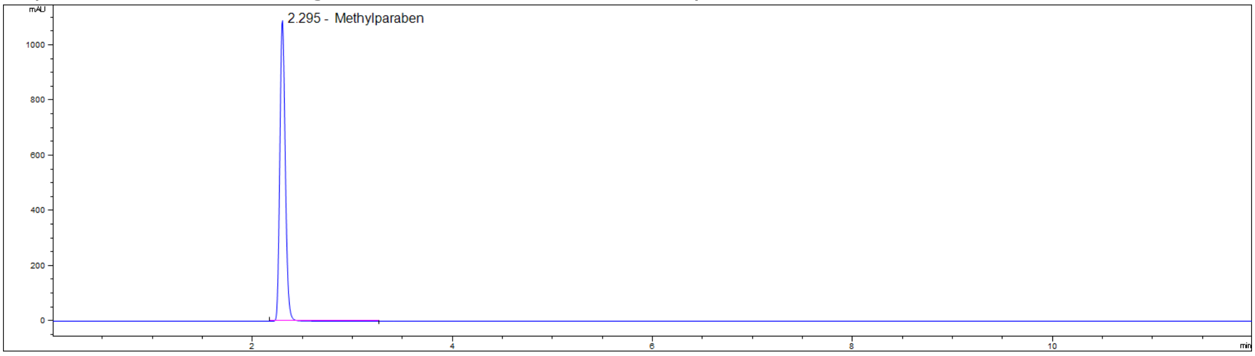

Certification process details:

The certified purity is determined by mass balance and calculated as

$$\% \, Purity = (\mathbf{100 - ROI - LOD - H_2O - RS}) * \left(\frac{\mathbf{100 - \textit{TCI}}}{\mathbf{100}}\right)$$

- TCI = Total Chromatographic Impurities
- LOD = Loss on Drying
- H_2O = Water content determined by Karl Fischer analysis
- ROI = Residue on Ignition
- RS = Residual Solvents

Methods for impurity determination may be added or deleted as required. The following techniques are applied:

CHROMATOGRAPHIC IMPURITY ANALYSIS

METHOD: HPLC (ref.: Methylparaben, Current Compendial Monographs)

See HPLC Assay

Impurities Detected:

Impurity 1:	0.015 %
4-hydroxybenzoic Acid:	0.007 %
Total Impurities:	**0.022 %**

Representative Chromatogram from Lot: LRAD2978 Impurity Analysis

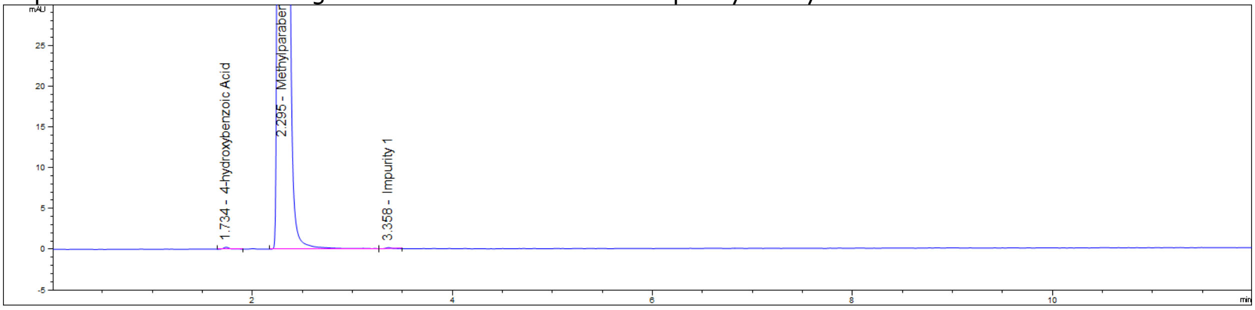

RESIDUAL SOLVENTS
Method: GC-MS Headspace (ref.: Adapted from Residual Solvents USP <467>)
Column: SPB-624, 30 m x 0.25 mm x 1.4 µm
Carrier gas: He
Flow: 1.0 mL/min
Split Ratio: 5:1
Injection/Temperature: 1 mL/180 °C
Temperature Program: 40 °C for 5 min, 8 °C/min to 200 °C, hold 5 min

Solvents Detected: **None**

WATER DETERMINATION
Method: Karl Fischer (ref.: Current Compendial Monographs)
Mean of three measurements, Water Content = **None**

RESIDUE ANALYSIS
Method: Sulfated Ash (ref.: Current Compendial Monographs)
Sample Size: ~ 300 mg
Mean of three measurements, Residue = **0.032 %**

CERTIFIED PURITY BY MASS BALANCE

99.9 % U_{crm} = ± 0.2 %, k = 2.0

(as is basis)

Homogeneity assessment: Homogeneity was assessed in accordance with ISO Guide 35. Completed units were sampled using a random stratified sampling protocol. The results of chemical analysis were then compared by Single Factor Analysis of Variance (ANOVA). The uncertainty due to homogeneity was derived from the ANOVA. Heterogeneity was not detected under the conditions of the ANOVA.

Analytical method: HPLC

Sample size: 10 mg

Stability assessment: Significance of the stability assessment will be demonstrated if the analytical result of the study and the range of values represented by the Expanded Uncertainty do not overlap the result of the original assay and the range of its values represented by the Expanded Uncertainty. The method employed will usually be the same method used to characterize the assay value in the initial evaluation.

Long Term Stability Evaluation - An assessment, or re-test, versus a Compendial Reference Standard may be scheduled, within the 3 year anniversary date of a release of a Secondary Standard. The re-test interval will be determined on a case-by-case basis. Short Term Stability Study - It is useful to assess stability under reasonably anticipated, short term transport conditions by simulating exposure of the product to humidity and temperature stress. This type of study is conducted under controlled conditions of elevated temperature and humidity.

Identification Test:

INFRARED SPECTROPHOTOMETRY (Comparative identification analysis demonstrates direct traceability to Pharmacopeial standards)

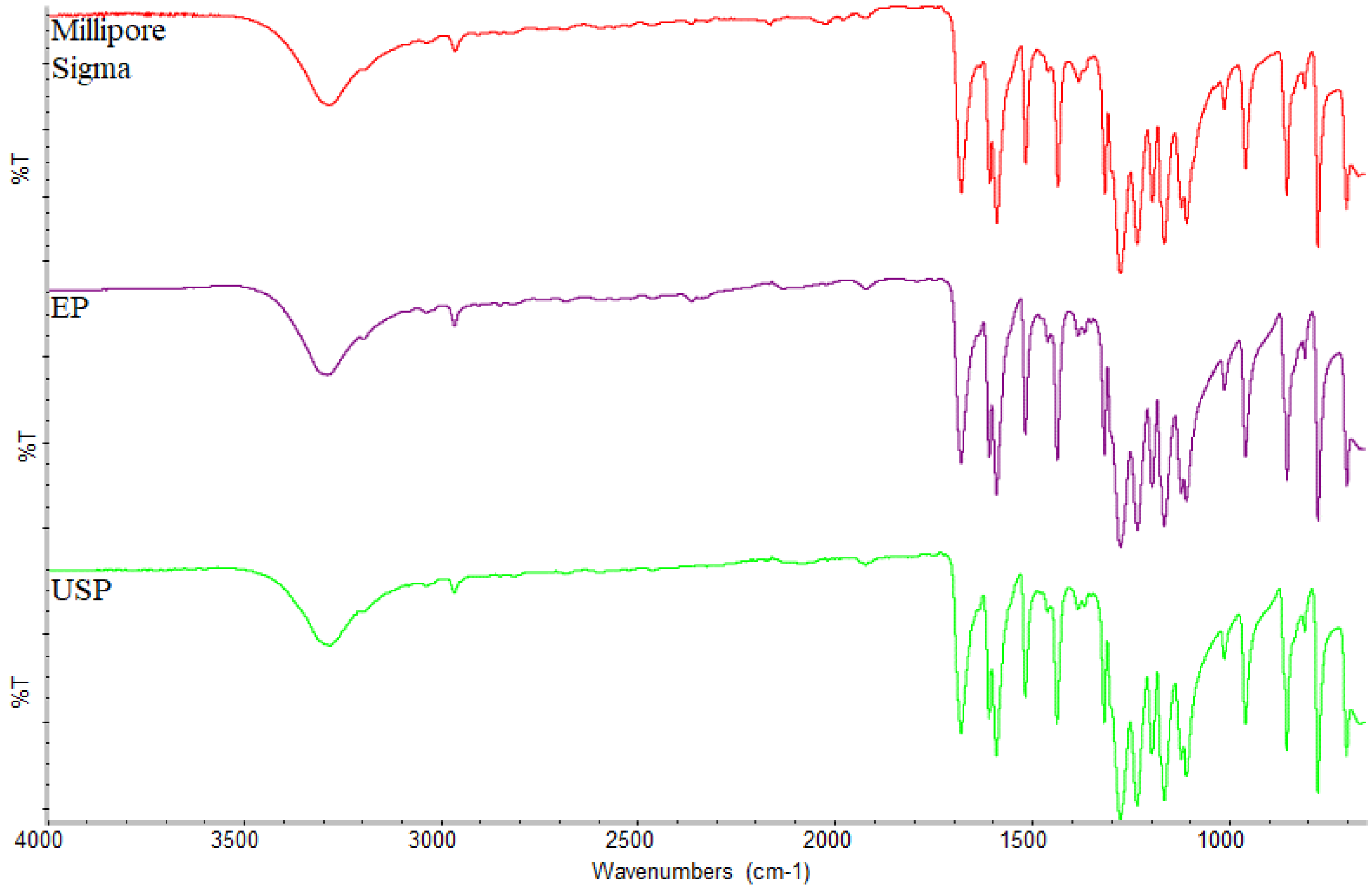

MilliporeSigma Lot LRAD2978 vs. EP Batch 5.0 / USP Lot R098B0

Indicative Values:

MASS SPECTRUM

Method: HR-QTOF; 4.0 kV ESI+; temperature: 325 °C

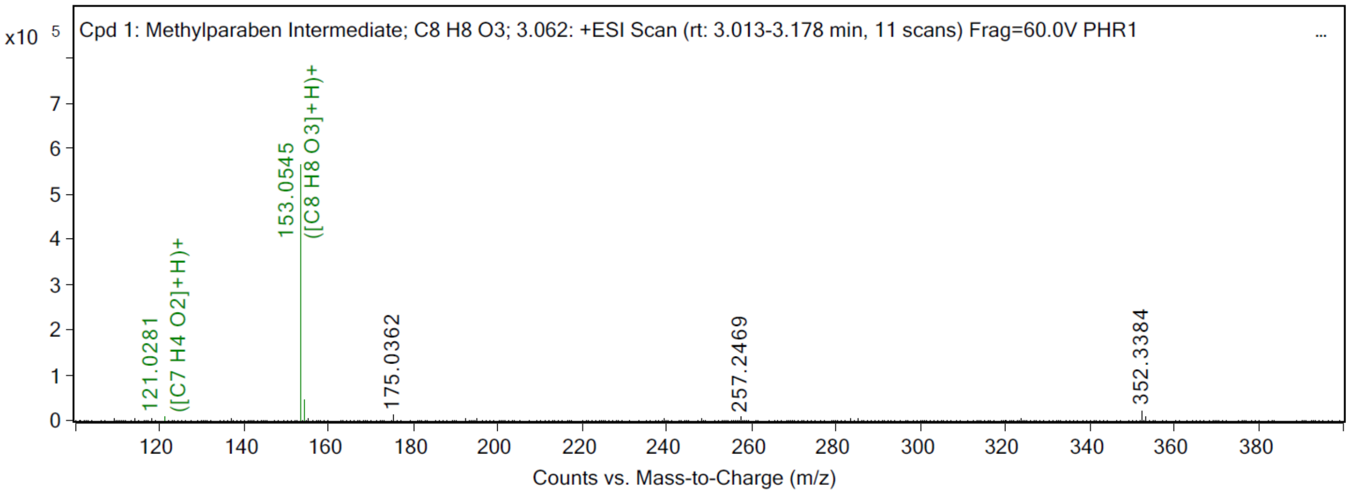

Theoretical value: 153.0551 m/z

The signal of the MS spectrum is consistent with the theoretical value and its interpretation is consistent with the structural formula.

[1]**H NMR** (Data provided by an external laboratory; not in scope of accreditation)

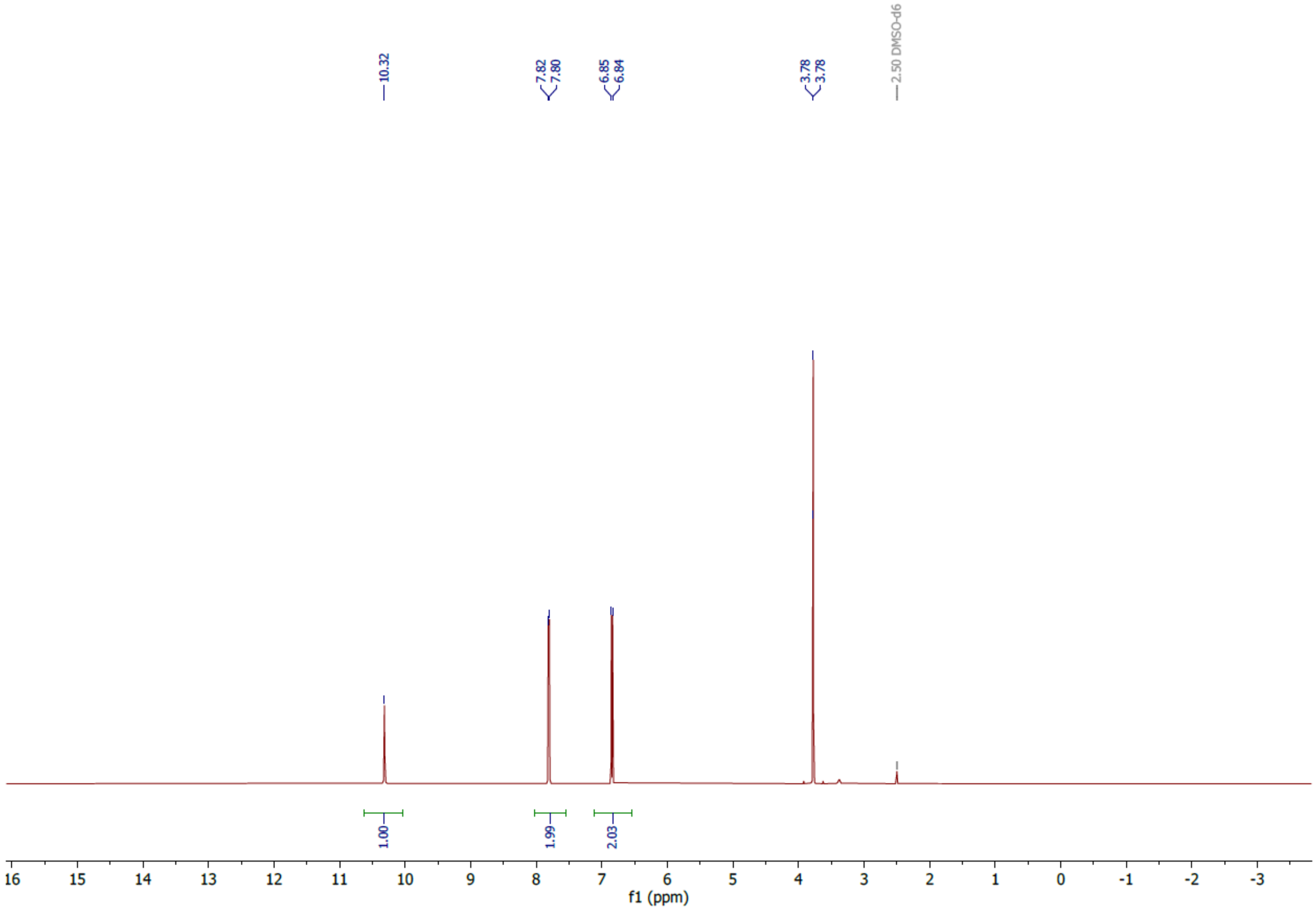

Consistent with structure

MELTING POINT
Specification: 125 – 128 °C (USP)
Mettler Toledo FP900 Thermosystem with FP81 Measuring Cell
Mean of three measurements = **126.13 – 127.40 °C**

Certificate of analysis revision history:

Certificate version	Date	Reason for version
LRAD2978.01	25 October 2022	Original Release

Disclaimer:

The purchaser is required to determine the suitability of this product for any particular application. Sigma-Aldrich RTC makes no warranty of any kind, express or implied, other than its products meet all quality control standards set by Sigma-Aldrich RTC. We do not guarantee that the product can be used for any particular application.

The vibrant M, Supelco, TraceCERT and Sigma-Aldrich are trademarks of Merck KGaA, Darmstadt, Germany or its affiliates.
All other trademarks are the property of their respective owners.
Detailed information on trademarks is available via publicly accessible resources.
© 2018 Merck KGaA, Darmstadt, Germany and/or its affiliates. All Rights Reserved.

The life science business of Merck KGaA, Darmstadt, Germany operates as MilliporeSigma in the US and Canada.
