# Supplementary material for: Challenging the Database: Day-of-Analysis Calibration and UF Modeling for Reliable RRF Use in Medical Device Chemical Characterization
Source: Anal Chem. 2025 Oct 8;97(41):22719–29. doi: 10.1021/acs.analchem.5c04247 (PMC12547855; doi:10.1021/acs.analchem.5c04247)
Supplement: Supplementary file 2 [file ac5c04247_si_002.zip › COA TRC Methyl-d3 Paraben M325662 3-PRN-164-1.pdf]

Toronto Research Chemicals
products for innovative research

CERTIFICATE OF ANALYSIS

1. Identification

Catalogue Number
M325662

Lot Number
3-PRN-164-1

Structure

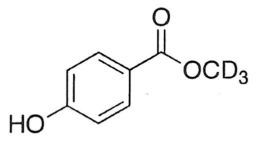

Product Name
Methyl-d3 Paraben

Synonyms
4-Hydroxybenzoic Acid Methyl-d3 Ester; 4-(Carbomethoxy-d3)phenol; 4-(Methoxycarbonyl-d3)phenol; 4-Hydroxybenzoic Acid Methyl-d3 Ester; 4-Hydroxymethyl-d3 Benzoate; Danisol M-d3; E 218-d3; Killitol-d3; Maseptol-d3; Mekkings M-d3; Metaben-d3; Metagin-d3; Paridol-d3; Preserval-d3; Solbrol-d3;

CAS Number
1216543-26-8

Solubility
Chloroform (Slightly), Methanol (Slightly)

Shipping Condition
This Product Is Stable To Be Shipped At Room Temperature

Molecular Formula
$C_8H_5D_3O_3$

Source of Product
Synthetic

Long Term Storage Conditions
4°C

Molecular Weight
155.17

Purity
98%

2. Warnings

Not Determined

3. Analytical Information

Tests	Specifications	Results
Appearance	White to Off-White Solid	White to Off-White Solid
NMR	Conforms to Structure	Conforms
Elemental Analysis	Conforms	%C: 61.93, %H: 5.21
HPLC Purity	Report Result	99.95% (255 nm)
MS	Conforms to Structure	Conforms
Isotopic Purity	>95%	99.9%

Additional Information

Normalized Intensity: d_0 = 0.02%, d_1 = 0.03%, d_2 = 0.25%, d_3 = 99.71%

Purity is based on the analytical results of the tests performed. NMR and Elemental Analysis (if available) may have an accuracy of ± 2%. Isotopic purity is based on mass distribution observed.
The contents of the specifications are subject to change without advance notice, and the specification values displayed here are the most up to date values.

4. Signatures

Reviewed by	Reviewed by	C of A Approved by	Test Date	Retest Date
[signature]	[signature]	[signature]	1/25/2024	1/23/2028
Sarbjeet Kaur	Toni Rantanen	Chanell Chu		
Product Quality Specialist	Manager, Quality Assurance	Quality Assurance Associate		
