# Supplementary material for: Challenging the Database: Day-of-Analysis Calibration and UF Modeling for Reliable RRF Use in Medical Device Chemical Characterization
Source: Anal Chem. 2025 Oct 8;97(41):22719–29. doi: 10.1021/acs.analchem.5c04247 (PMC12547855; doi:10.1021/acs.analchem.5c04247)
Supplement: Supplementary file 2 [file ac5c04247_si_002.zip › coa_20250211055842.pdf]

Certificate of Analysis

02/11/2025(JST)

TOKYO CHEMICAL INDUSTRY CO.,LTD.
T-PLUS Nihonbashi-Kodemmacho
16-12 Nihonbashi-kodemmacho, Chuo-ku, Tokyo 103-0001, Japan

Chemical Name: ***N*-Lauryldiethanolamine**		
Product Number: L0020<br>CAS RN: 1541-67-9	Lot: 6MGZJ	

Tests	Results	Specifications
Appearance	White lump	White or Colorless to Light yellow to Light orange powder to lump to clear liquid
Purity(GC)	98.1 %	min. 95.0 %

TCI Lot numbers are 4-5 characters in length. Characters listed after the first 4-5 characters are control numbers for internal purpose only.
The contents of the specifications are subject to change without advance notice. The specification values displayed here are the most up to date values. There may be cases where the product labels display a different specification, however, the product quality still meets the latest specification.

Customer Service:

TCI AMERICA
Tel: +1-800-423-8616 / +1-503-283-1681
Fax: +1-888-520-1075 / +1-503-283-1987
E-mail: Sales-US@TCIchemicals.com

Takuya Nishioka
Quality Assurance Department Manager
