# Supplementary material for: Challenging the Database: Day-of-Analysis Calibration and UF Modeling for Reliable RRF Use in Medical Device Chemical Characterization
Source: Anal Chem. 2025 Oct 8;97(41):22719–29. doi: 10.1021/acs.analchem.5c04247 (PMC12547855; doi:10.1021/acs.analchem.5c04247)
Supplement: Supplementary file 2 [file ac5c04247_si_002.zip › COA_Irganox_3114_European _Pharmacopoeia_P2155013_Batch3.pdf]

European Directorate for the Quality of Medicines & HealthCare
European Pharmacopoeia (Ph. Eur.)
7, Allée Kastner CS 30026, F-67081 Strasbourg (France)
Tel. +33 (0)3 88 41 20 35   Fax. + 33 (0)3 88 41 27 71
For any questions: www.edqm.eu (HelpDesk)

INFORMATION LEAFLET Ph. Eur. Reference Standard

PLASTIC ADDITIVE 13 CRS batch 3

1. Identification

Catalogue code: P2155013

2. Scientific Information

2.1 Intended use

Reference Standard for laboratory tests as prescribed in the European Pharmacopoeia only.
Established for use with the monograph(s): 30103, 30105, 30106.

2.2 Analytical information related to intended use, when applicable

2.3 Uncertainty of the assigned value, when applicable

The uncertainty of the assigned value is not stated since it is considered to be negligible in relation to the defined limits of the method-specific assays for which the reference standard is used. Please also refer to Ph. Eur. chapter 5.12.

2.4 Validity

Ph. Eur. RS are periodically tested to ensure their continuous fitness for purpose. For each valid Ph. Eur. RS, a Batch Validity Statement at the time of use can be downloaded and printed from the EDQM website (Reference Standards Database).

2.5 Instructions for use

The container should not be opened until required for use. Allow the closed container to equilibrate at ambient temperature before opening to avoid uptake of moisture. Use "as is". Do not dry/desiccate before use. Ph. Eur. RS are for immediate use. Once the container has been opened, its entire content must be used immediately. Any further storage and re-use are not warranted.

3. Storage conditions

In the original container at +5°C ± 3°C, protected from light. Re-instate promptly upon receipt.

4. Safety

For scientific research, development and analysis only. Handle in accordance with good occupational hygiene, safety and laboratory practices and take precautions to avoid exposure. More information is available at the EDQM website (Reference Standards Database): Safety Data Sheet for hazardous chemicals and Safety Data Statement for other materials.

5. Shipping conditions

Each Ph. Eur. RS is shipped under conditions that preserve its suitability for use and comply with the relevant regulations. For more details see EDQM website (Reference Standards Database).

6. Warranties, Liabilities and responsibility

- Safety
In the event of any safety concerns, please read carefully the safety data sheets or safety data statements available for each product. It is for Purchasers to determine independently the risks associated with the items and to take appropriate safety measures, including the provision of appropriate information, equipment and training of those persons coming into contact with the item.

- Warranties
Except for the use of Reference Standards in tests and assays carried out in accordance with the official methods of the European Pharmacopoeia and by professionals with the necessary technical skills and at their own discretion and risk, the EDQM makes no representation, contractual statement, or expression of opinion concerning the quality or safety of any item supplied, the presence of any defect in it, or its fitness for any particular purpose except that as described above.

The EDQM does not guarantee that the items will meet the Purchaser's specific expectations. The EDQM only guarantees that the items (i) were fit for use according to EDQM's intended use of the product ;(ii) were fit for use at the moment that they were handed over to the carrier being responsible for the delivery of the items to the Purchaser with such accessories including packaging, delivery instructions or other instructions for the item's delivery and reception as the Purchaser may expect to receive; and (iii) possess qualities and performance capabilities which are normal in goods of the same type and which the Purchaser may expect given the nature of the goods and the information provided on the EDQM's website and (iv) the carrier and the Purchaser received clear and accurate instructions for the item's delivery and reception. No other guarantees, whether explicitly or implied, are given by the EDQM. The EDQM does not guarantee that the purchase or use of the items will not infringe any intellectual property rights, in particular patents.

– Limitation of Liability
In no event shall the EDQM be liable for any damages due to the use of items, included, but not limited to loss of business, loss of profit, loss of use, loss of opportunity, costs of procurement of substitute goods, services or systems or for any indirect, special, incidental, punitive or consequential damages, however caused and, whether in contract, tort or under any other theory of liability, whether or not the Purchaser has been advised of the possibility of such damages or costs.
Any liability of the EDQM for injury, loss or damage arising from the supply or use of any such item is in any event hereby excluded to the fullest extent permitted internationally accepted commercial standards; in particular, no liability is accepted for loss of profits or indirect or consequential loss.

7. Arbitration & Applicable Law

The aim of the EDQM is to settle any disputes amicably in the framework of its Terms and Conditions. In accordance with the provisions of article 21 of the General Agreement on the Privileges and Immunities of the Council of Europe, all disputes between the EDQM and the Purchaser as regards the application of these General Terms shall be submitted, if a mutual agreement cannot be reached between the parties, to arbitration as laid down in Order No. 481 of the Secretary General, approved by the Committee of Ministers.
This transaction shall be governed by the Council of Europe's relevant regulatory framework, complemented, where necessary, by French national substantive law.

8. Citation

Users shall ensure that any reference made to an EDQM Reference Standard in any publication, presentation or public document (ex. scientific articles, data sheets for kits) bears the exact name, and catalogue code of the Reference Standard and the exact name and address of EDQM as given on the first page of this information leaflet.

9. Adoption

The suitability for intended use has been officially adopted by the European Pharmacopoeia Commission.

10. Signature

This document is approved by:

Ms Caroline Offerlé
Head of the Quality and Risk Management Section
