# Supplementary material for: Challenging the Database: Day-of-Analysis Calibration and UF Modeling for Reliable RRF Use in Medical Device Chemical Characterization
Source: Anal Chem. 2025 Oct 8;97(41):22719–29. doi: 10.1021/acs.analchem.5c04247 (PMC12547855; doi:10.1021/acs.analchem.5c04247)
Supplement: Supplementary file 2 [file ac5c04247_si_002.zip › Dipentyl phthalate 80154 BCCD6920 Sigma aldrich.pdf]

SIGMA-ALDRICH

3050 Spruce Street, Saint Louis, MO 63103 USA
Email USA: techserv@sial.com Outside USA: eurtechserv@sial.com

Certificate of Analysis

**Product Name:**	Dipentyl phthalate<br>Selectophore®, >= 99.0 %
**Product Number:**	80154
**Batch Number:**	BCCD6920
**Brand:**	Sigma-Aldrich
**CAS Number:**	131-18-0
**Formula:**	$C_{18}H_{26}O_4$
**Formula Weight:**	306.40
**Quality Release Date:**	13 AUG 2020
**Recommended Retest Date:**	JUL 2026

TEST	SPECIFICATION	RESULT
**APPEARANCE (COLOR)**	COLORLESS TO VERY FAINT YELLOW	COLORLESS
**APPEARANCE (FORM)**	LIQUID	LIQUID
**PURITY (GC AREA %)**	≥ 99.0 %	99.4 %
**REFRACTIVE INDEX N20/D**	1.489 - 1.491	1.490
**PROTON NMR SPECTRUM**	CONFORMS TO STRUCTURE	CONFORMS

Dr. Reinhold Schwenninger
Quality Assurance
Buchs, Switzerland

Sigma-Aldrich warrants that at the time of the quality release or subsequent retest date this product conformed to the information contained in this publication. The current specification sheet may be available at Sigma-Aldrich.com. For further inquiries, please contact Technical Service. Purchaser must determine the suitability of the product for its particular use. See reverse side of invoice or packing slip for additional terms and conditions of sale.
