# Supplementary material for: Challenging the Database: Day-of-Analysis Calibration and UF Modeling for Reliable RRF Use in Medical Device Chemical Characterization
Source: Anal Chem. 2025 Oct 8;97(41):22719–29. doi: 10.1021/acs.analchem.5c04247 (PMC12547855; doi:10.1021/acs.analchem.5c04247)
Supplement: Supplementary file 2 [file ac5c04247_si_002.zip › Dodecanoic acid 61609 BCCM3172.pdf]

3050 Spruce Street, Saint Louis, MO 63103, USA
Website: www.sigmaaldrich.com
Email USA: techserv@sial.com
Outside USA: eurtechserv@sial.com

Certificate of Analysis

Product Name:
Dodecanoic acid - analytical standard

**Product Number:**	**61609**
**Batch Number:**	**BCCM3172**
Brand:	SIAL
CAS Number:	143-07-7
Formula:	C12H24O2
Formula Weight:	200,32 g/mol
Quality Release Date:	27 SEP 2024
Expiration Date:	AUG 2027

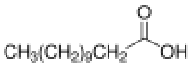

Test	Specification	Result
Appearance (Color)	Colorless or White	White
Appearance (Form)	Powder or Crystals or Flakes	Crystals
Purity (GC)	$\geq$ 99.0 %	99.8 %
Melting Point	43 - 46 ℃	45 ℃
1H NMR Spectrum	Conforms to Structure	Conforms

Dr.Reinhold Schwenninger
Quality Assurance
Buchs,Switzerland CH

Sigma-Aldrich warrants, that at the time of the quality release or subsequent retest date this product conformed to the information contained in this publication. The current Specification sheet may be available at Sigma-Aldrich.com. For further inquiries, please contact Technical Service. Purchaser must determine the suitability of the product for its particular use. See reverse side of invoice or packing slip for additional terms and conditions of sale.
