# Supplementary material for: Challenging the Database: Day-of-Analysis Calibration and UF Modeling for Reliable RRF Use in Medical Device Chemical Characterization
Source: Anal Chem. 2025 Oct 8;97(41):22719–29. doi: 10.1021/acs.analchem.5c04247 (PMC12547855; doi:10.1021/acs.analchem.5c04247)
Supplement: Supplementary file 2 [file ac5c04247_si_002.zip › Hexadecamethylcyclooctasiloxane Lot # V7YQC-UC H1288 TCI.pdf]

Certificate of Analysis

12/22/2022(JST)

TOKYO CHEMICAL INDUSTRY CO.,LTD.
4-10-1 Nihonbashi-Honcho, Chuo-ku, Tokyo 103-0023 Japan

**Chemical Name: Hexadecamethylcyclooctasiloxane**		
Product Number: H1288<br>CAS RN: 556-68-3	Lot: V7YQC	

Tests	Results	Specifications
Appearance	White lump	White or Colorless to Almost white or Almost colorless powder to lump to clear liquid
Purity(GC)	99.8 %	min. 96.0 %

TCI Lot numbers are 4-5 characters in length. Characters listed after the first 4-5 characters are control numbers for internal purpose only.
The contents of the specifications are subject to change without advance notice. The specification values displayed here are the most up to date values. There may be cases where the product labels display a different specification, however, the product quality still meets the latest specification.

Customer Service:

TOKYO CHEMICAL INDUSTRY CO., LTD
E-mail: globalbusiness@TCIchemicals.com

Takuya Nishioka
Quality Assurance Department Manager
