# Supplementary material for: Challenging the Database: Day-of-Analysis Calibration and UF Modeling for Reliable RRF Use in Medical Device Chemical Characterization
Source: Anal Chem. 2025 Oct 8;97(41):22719–29. doi: 10.1021/acs.analchem.5c04247 (PMC12547855; doi:10.1021/acs.analchem.5c04247)
Supplement: Supplementary file 2 [file ac5c04247_si_002.zip › Irganox 1098 Ambeed A244037_A244037-QH5_97_NMR.pdf]

Ambeed, Inc.

Certificate of Analysis

Compound Information

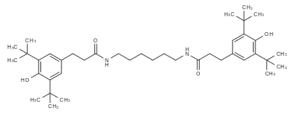

Product Name:	N,N'-(Hexane-1,6-diyl)bis(3-(3,5-di-tert-butyl-4-hydroxyphenyl)propanamide)		
Cat. No.:	A244037	Formula:	C40H64N2O4
CAS No.:	23128-74-7	MW:	636.95

Batch Information

Batch Number:	A244037-QH5	Mfg. Date:	Jan 13, 2022
Report Date:	Jan 13, 2022	Retest Date:	Dec 23, 2025

QC Test Information

Items	Result
Appearance	White Solid
1H NMR Spectrum	Conforms
Water(KF)(%)	0.146%
Melting Point (°C)	157.8-159.7°C
Purity (NMR)	97%

Analyst: Sayah Li

Approved: Marry Zhang

Sarah Li

Marry Zhang

Product Url: http://www.ambeed.com/products/23128-74-7.html

For Laboratory Use Only !

630-580-1088 630-581-0788 sales@ambeed.com www.ambeed.com

3205 N WILKE Rd, STE 3205-125, Arlington Hts, IL 60004, USA
