# Supplementary material for: Challenging the Database: Day-of-Analysis Calibration and UF Modeling for Reliable RRF Use in Medical Device Chemical Characterization
Source: Anal Chem. 2025 Oct 8;97(41):22719–29. doi: 10.1021/acs.analchem.5c04247 (PMC12547855; doi:10.1021/acs.analchem.5c04247)
Supplement: Supplementary file 2 [file ac5c04247_si_002.zip › Restek SVOC ISTDs A0214427.pdf]

RESTEK

110 Benner Circle
Bellefonte, PA 16823-8812
Tel: 1-814-353-1300
Fax: 1-814-353-1309

www.restek.com

CERTIFIED REFERENCE MATERIAL

Certificate of Analysis

chromatographic plus

ilac-MRA ACCREDITED ISO 17034 Accredited Reference Material Producer Certificate #3222.01

ilac-MRA ACCREDITED ISO/IEC 17025 Accredited Testing Laboratory Certificate #3222.02

FOR LABORATORY USE ONLY-READ SDS PRIOR TO USE.

This Reference Material is intended for Laboratory Use Only as a standard for the qualitative and/or quantitative determination of the analyte(s) listed.

Catalog No. : 579263 **Lot No.:** A0214427

Description : Custom Deuterated SVOC Standard

Custom Deuterated SVOC Standard 1,000µg/mL, Ethyl acetate/Methylene Chloride (90:10), 1mL/ampul

Container Size : 2 mL **Pkg Amt:** > 1 mL

Expiration Date : July 31, 2026 **Storage:** 10°C or colder

Handling: Sonication required. Mix is photosensitive. **Ship:** Ambient

CERTIFIED VALUES

Elution Order	Compound	CAS #	Lot #	Purity	Grav. Conc. (weight/volume)	Expanded Uncertainty * (95% C.L.; K=2)
1	Acetophenone-d8	19547-00-3	G-466	99%	1,005.0 µg/mL	+/- 34.7496
2	Dibutyl phthalate-d4	93952-11-5	KL-167	99%	1,006.4 µg/mL	+/- 34.7980
3	Chrysene-d12	1719-03-5	PR-33506	99%	1,004.0 µg/mL	+/- 34.7150

* Expanded Uncertainty displayed in same units as Grav. Conc.

Solvent: Ethyl acetate/Methylene Chloride (90:10)

CAS # 141-78-6/75-09-2

Purity 99%

Quality Confirmation Test

Column:
30m x 0.25mm x 0.25µm
Rtx-5 (cat.#10223)

Carrier Gas:
hydrogen-constant flow 1.8 mL/min.

Temp. Program:
80°C (hold 0.1 min.) to 330°C
@ 9.6°C/min. (hold 2.86 min.)

Inj. Temp:
250°C

Det. Temp:
340°C

Det. Type:
FID

Split Vent:
100 ml/min.

Inj. Vol
1µl

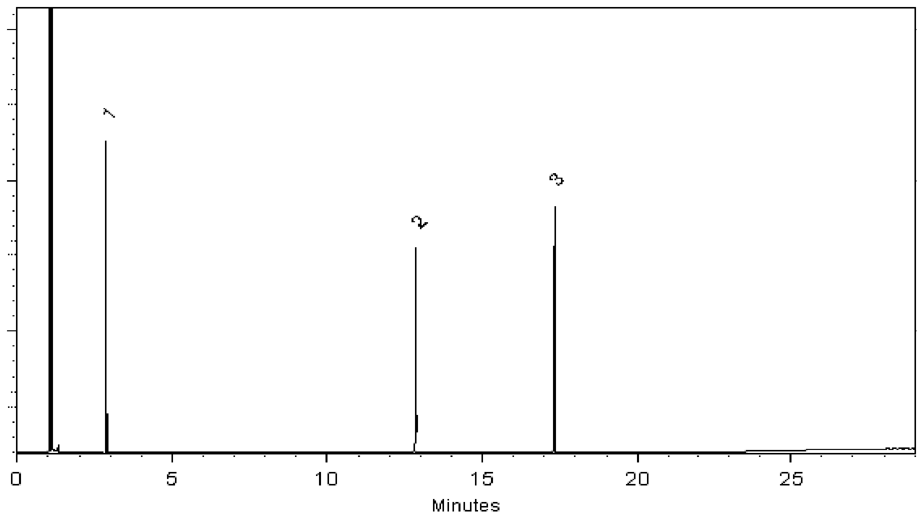

This chromatogram represents a general set of testing conditions chosen for product acceptance. For optimal results in your lab, conditions should be adjusted for your specific instrument, method, and application.

Penelope Riglin - Operations Tech I

Date Mixed: 25-Jul-2024 **Balance Serial #** 1128360905

Dillan Murphy - Operations Technician I

Date Passed: 01-Aug-2024

Manufactured under Restek's ISO 9001:2015 Registered Quality System Certificate #FM 80397

General Certified Reference Material Notes

Expiration Notes:

- Expiration date valid for unopened ampul stored in compliance with the recommended conditions.
- Uncertainty, concentration, and expiration of the CRM are based on the unopened product being stored according to the recommended condition found in the storage field.

Purity Notes:

- Purity and/or chemical identity are determined by one or more of the following techniques: GC/FID, HPLC, GC/μECD, GC/MS, LC/MS, RI, and/or melting point.
- Compounds with a listed purity of less than 99% have been weight corrected to compensate for impurities and/or salts. A correction factor is used to calculate the amount of compound necessary to achieve the desired concentration of the parent compound in solution.
- Purity of isomeric compounds is reported as the sum of the isomers.
- Purity values are rounded to the nearest whole number.

Certified Uncertainty Value Notes:

- The uncertainties are determined in accordance with ISO 17034 and Guide 35. The certified expanded uncertainty value includes gravimetric uncertainty, homogeneity between-ampul uncertainty, storage stability uncertainty and shipping stability uncertainty and were combined using the following formula:

$$U_{combined\ uncertainty} = k\sqrt{u^2_{gravimetric} + u^2_{homogeneity} + u^2_{storage\ stability} + u^2_{shipping\ stability}}$$

k is a coverage factor of 2, which gives a level of confidence of approximately 95%.

- The packaged amount is the minimum sample size for which uncertainty is valid. The ampuls are over-filled to ensure that the minimum packaged amount can be sufficiently transferred.

Manufacturing Notes:

- Concentration is based upon gravimetric preparation using either a balance whose calibration has been verified daily using NIST traceable weights, and/or dilutions with Class A glassware.

Handling Notes:

- Stability of the unopened product, when stored in compliance with the recommended conditions, is guaranteed through the expiration displayed on the product label and certificate. Contact Restek for additional opened product stability information, with the knowledge/understanding that open product stability is subject to the specific handling and environmental conditions to which the product is exposed. For your convenience Restek supplies deactivated vials with most standards packed in 2mL ampuls. Larger volume deactivated vials are available through Restek as a custom ordered item. Additionally, Restek sells DMDCS for the purpose of glassware deactivation as catalog number 31861, which includes complete instructions.
- If any undissolved material is visible inside the ampul, sonicate the unopened ampul until the material is completely dissolved.
