# Supplementary material for: Challenging the Database: Day-of-Analysis Calibration and UF Modeling for Reliable RRF Use in Medical Device Chemical Characterization
Source: Anal Chem. 2025 Oct 8;97(41):22719–29. doi: 10.1021/acs.analchem.5c04247 (PMC12547855; doi:10.1021/acs.analchem.5c04247)
Supplement: Supplementary file 2 [file ac5c04247_si_002.zip › SVOC CoA.pdf]

Reference Standard Data Pack

Custom SVOC Standard

Catalog # 579551

Lot # A0207258

110 Benner Circle Bellefonte, PA 16823-8812
1-814-353-1300   1-800-356-1688
www.restek.com

FOR LABORATORY USE ONLY. READ SDS PRIOR TO USE.

RAW MATERIAL TEST INFORMATION AVAILABLE UPON REQUEST

MANUFACTURED UNDER RESTEK'S ISO 9001 REGISTERED QUALITY SYSTEM

REVIEWED

110 Benner Circle
Bellefonte, PA 16823-8812
Tel: 1-814-353-1300
Fax: 1-814-353-1309

www.restek.com

Certificate of Analysis

chromatographic plus

FOR LABORATORY USE ONLY-READ SDS PRIOR TO USE.

This Reference Material is intended for Laboratory Use Only as a standard for the qualitative and/or quantitative determination of the analyte(s) listed.

Catalog No. : 579551 **Lot No.:** A0207258

Description : Custom SVOC Standard

Custom SVOC Standard 500µg/mL, Ethyl acetate/Methylene Chloride (90:10), 5mL/ampul

Container Size : 5 mL **Pkg Amt:** > 5 mL

Expiration Date : July 31, 2025 **Storage:** 0°C or colder

Handling: Sonication required. Mix is photosensitive. **Ship:** Ambient

Elution Order	Compound	CAS #	Percent Purity	Grav. Conc. (weight/volume)	Expanded Uncertainty (95% C.L.; K=2)
1	Phenol	108-95-2	99%	503.3 µg/mL	+/- 2.9896 µg/mL
2	1-Decene	872-05-9	98%	501.1 µg/mL	+/- 2.9764 µg/mL
3	Acetophenone	98-86-2	99%	503.3 µg/mL	+/- 2.9896 µg/mL
4	1-dodecene	112-41-4	98%	500.5 µg/mL	+/- 2.9725 µg/mL
5	epsilon-Caprolactam	105-60-2	99%	502.7 µg/mL	+/- 2.9857 µg/mL
6	Acenaphthylene	208-96-8	95%	501.0 µg/mL	+/- 2.9756 µg/mL
7	2,6-Di-tert-butyl-4-methylphenol (BHT)	128-37-0	99%	502.7 µg/mL	+/- 2.9857 µg/mL
8	1-Hexadecene	629-73-2	99%	504.0 µg/mL	+/- 2.9936 µg/mL
9	Diethylphthalate	84-66-2	99%	502.0 µg/mL	+/- 2.9817 µg/mL
10	Diphenylamine	122-39-4	99%	501.3 µg/mL	+/- 2.9778 µg/mL
11	Benzophenone	119-61-9	99%	504.0 µg/mL	+/- 2.9936 µg/mL
12	Methyl Palmitate (C16:0)	112-39-0	99%	500.7 µg/mL	+/- 2.9738 µg/mL
13	Di-n-butylphthalate	84-74-2	99%	504.0 µg/mL	+/- 2.9936 µg/mL
14	n-Eicosane (C20)	112-95-8	97%	501.8 µg/mL	+/- 2.9806 µg/mL
15	Methyl Oleate (C18:1 cis 9)	112-62-9	98%	502.4 µg/mL	+/- 2.9842 µg/mL
16	4,4'-Diaminodiphenylmethane	101-77-9	99%	502.7 µg/mL	+/- 2.9857 µg/mL
17	Ethyl octadecanoate	111-61-5	99%	502.0 µg/mL	+/- 2.9817 µg/mL
18	Bis(2-ethylhexyl)adipate	103-23-1	99%	500.7 µg/mL	+/- 2.9738 µg/mL
19	Chrysene	218-01-9	99%	502.7 µg/mL	+/- 2.9857 µg/mL
20	Bis(2-ethylhexyl)phthalate	117-81-7	99%	502.7 µg/mL	+/- 2.9857 µg/mL
21	n-Octacosane (C28)	630-02-4	99%	502.7 µg/mL	+/- 2.9857 µg/mL
22	Indeno(1,2,3-cd)pyrene	193-39-5	97%	502.5 µg/mL	+/- 2.9845 µg/mL

Solvent: Ethyl acetate/Methylene Chloride (90:10) 141-78-6/75-09-2 99%

Tech Tips:

N-Nitrosodiphenylamine (86-30-6) is prone to breakdown in the injection port and will be converted to Diphenylamine (122-39-4). When comparing the response of Diphenylamine to mixtures manufactured using N-Nitrosodiphenylamine, a difference in response will be observed. The ratio of the MW can be used to calculate the theoretical concentration of the N-Nitrosodiphenylamine.

Quality Confirmation Test

Column:
30m x 0.25mm x 0.25µm
Rtx-5 (cat.#10223)

Carrier Gas:
hydrogen-constant flow 1.8 mL/min.

Temp. Program:
80°C (hold 0.1 min.) to 330°C
@ 9.6°C/min. (hold 2.86 min.)

Inj. Temp:
250°C

Det. Temp:
340°C

Det. Type:
FID

Split Vent:
100 ml/min.

Inj. Vol
1µl

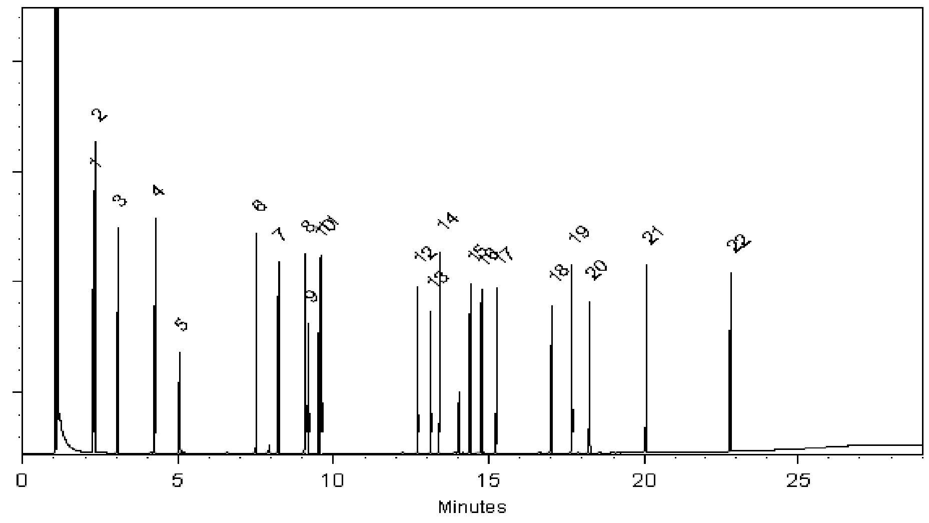

This chromatogram represents a general set of testing conditions chosen for product acceptance. For optimal results in your lab, conditions should be adjusted for your specific instrument, method, and application.

Daniel Wasson - Operations Tech I

Date Mixed: 31-Jan-2024 **Balance Serial #** 1128353505

Jennifer Pollino - Operations Tech III - ARM QC

Date Passed: 08-Feb-2024

**Manufactured under Restek's ISO 9001:2015
Registered Quality System
Certificate #FM 80397**

General Reference Material Notes

Expiration Notes:

- Expiration date valid for unopened ampul stored in compliance with the recommended conditions.
- Uncertainty, concentration, and expiration of the RM are based on the unopened product being stored according to the recommended condition found in the storage field.

Purity Notes:

- Purity and/or chemical identity are determined by one or more of the following techniques: GC/FID, HPLC, GC/µECD, GC/MS, LC/MS, RI, and/or melting point.
- Compounds with a listed purity of less than 99% have been weight corrected to compensate for impurities and/or salts. A correction factor is used to calculate the amount of compound necessary to achieve the desired concentration of the parent compound in solution.
- Purity of isomeric compounds is reported as the sum of the isomers.
- Purity values are rounded to the nearest whole number.

Uncertainty Value Notes:

- Uncertainties are determined using data from balances and glassware, raw material purity, and, when significant, equipment tolerances or calibration results.

Manufacturing Notes:

- Concentration is based upon gravimetric preparation using either a balance whose calibration has been verified daily using NIST traceable weights, and/or dilutions with Class A glassware.

Handling Notes:

- Stability of the unopened product, when stored in compliance with the recommended conditions, is guaranteed through the expiration displayed on the product label and certificate. Contact Restek for additional opened product stability information, with the knowledge/understanding that open product stability is subject to the specific handling and environmental conditions to which the product is exposed. For your convenience Restek supplies deactivated vials with most standards packed in 2mL ampules. Larger volume deactivated vials are available through Restek as a custom ordered item. Additionally, Restek sells DMDCS for the purpose of glassware deactivation as catalog number 31861, which includes complete instructions.

RESTEK

Reference Material Batch Record
Lot #: A0207258

**Catalog #:** 579551	**Target:** 500 ug/mL		
**Description:** Custom SVOC Standard			
**Solvent:** Ethyl acetate/Methylene Chloride (90:10)	**Solvent Lot:** 21K1862004/233607	**Final Volume:**	150 ml

**Made by:** Daniel Wasson	**Date:** 2/6/2024 5:50:13PM		
**Tested by:** Alicia Leathers	**Date:** 2/7/2024 7:52:49AM		
Pass	**By:** Jennifer Pollino	**Date:** 2/8/2024 11:23:03AM	
**Packaged by:** Malina Homan / Daniel Wasson	**Date:** 2/6/2024 8:24:49PM	**No. Units:** 20	**Pkg Size:** 5 mL
**Balance Used:** BEDE001734 XP205	**Serial #:** 1128353505		

Compound	CAS	Storage Location	Lot #	Purity	Target Conc(ug/mL)	Target	Actual	Calc Conc(ug/mL)
n-Octacosane (C28)	630-02-4	R0536	BCCG0084	0.99	500.00	75.00 mg	75.40 mg	502.7
n-Eicosane (C20)	112-95-8	R0546	MKCN8767	0.97	500.00	77.32 mg	77.60 mg	501.8
Ethyl octadecanoate	111-61-5	F0298	N-18E-JA31-C	0.99	500.00	75.00 mg	75.30 mg	502.0
Methyl Oleate (C18:1 cis 9)	112-62-9	R0010	U-46M-O31-G	0.98	500.00	76.53 mg	76.90 mg	502.4
Acenaphthylene	208-96-8	R0497	L10L	0.95	500.00	78.95 mg	79.10 mg	501.0
Chrysene	218-01-9	R0505	RP230601	0.99	500.00	75.00 mg	75.40 mg	502.7
Methyl Palmitate (C16:0)	112-39-0	R0007	N-16M-N9-F	0.99	500.00	75.00 mg	75.10 mg	500.7
Indeno(1,2,3-cd)pyrene	193-39-5	R0521	12-JKL-118-9	0.97	500.00	77.32 mg	77.70 mg	502.5
epsilon-Caprolactam	105-60-2	R0814	I16X016	0.99	500.00	75.00 mg	75.40 mg	502.7
1-Hexadecene	629-73-2	R1605	015677/2	0.99	500.00	75.00 mg	75.60 mg	504.0
2,6-Di-tert-butyl-4-methylphenol (BHT)	128-37-0	VL-025	8022	0.99	500.00	75.00 mg	75.40 mg	502.7
1-dodecene	112-41-4	R1562	BCBR9583V	0.98	500.00	76.53 mg	76.60 mg	500.5
1-Decene	872-05-9	R1591	14315500	0.98	500.00	76.53 mg	76.70 mg	501.1
Benzophenone	119-61-9	R0719	0000233517	0.99	500.00	75.00 mg	75.60 mg	504.0
Diphenylamine	122-39-4	R0691	MKCT1512	0.99	500.00	75.00 mg	75.20 mg	501.3
Bis(2-ethylhexyl)adipate	103-23-1	R0660	MKCM1988	0.99	500.00	75.00 mg	75.10 mg	500.7
Diethylphthalate	84-66-2	R0631	MKCD2547	0.99	500.00	75.00 mg	75.30 mg	502.0
Phenol	108-95-2	R0627	MKCK1120	0.99	500.00	75.00 mg	75.50 mg	503.3
Acetophenone	98-86-2	R0561	STBH8205	0.99	500.00	75.00 mg	75.50 mg	503.3
Di-n-butylphthalate	84-74-2	R0508	MKCN4337	0.99	500.00	75.00 mg	75.60 mg	504.0
Bis(2-ethylhexyl)phthalate	117-81-7	R0479	MKCQ3468	0.99	500.00	75.00 mg	75.40 mg	502.7
4,4'-Diaminodiphenylmethane	101-77-9	R2336	YDVUO	0.99	500.00	75.00 mg	75.40 mg	502.7

QA Report: Custom SVOC Standard (Cat.#579551)

COMPONENT	Runs of Lot # A0194707 Run #1	Run #2	Run #3	AVG	STD DEV	% RSD	Runs of Lot # A0207258 Run #1	Run #2	Run #3	AVG	STD DEV	% RSD	%D MEAN	P/F
Phenol	85577	85515	86754	85949	698	0.81	86081	85578	85038	85566	522	0.61	0.45	PASS
1-Decene	101391	101579	102675	101882	693	0.68	103258	102778	101755	102597	768	0.75	-0.70	PASS
Acetophenone	88257	88084	89362	88568	693	0.78	88623	88336	87665	88208	492	0.56	0.41	PASS
1-dodecene	104195	104003	105471	104556	798	0.76	105940	105305	104490	105245	727	0.69	-0.66	PASS
epsilon-Caprolactam	63767	63617	64216	63867	312	0.49	64580	63453	64166	64066	570	0.89	-0.31	PASS
Acenaphthylene	122169	122004	124106	122760	1169	0.95	123378	122747	122027	122717	676	0.55	0.03	PASS
2,6-Di-tert-butyl-4-methylphenol (BHT)	101733	101488	103264	102162	962	0.94	104652	104341	103837	104277	411	0.39	-2.07	PASS
1-Hexadecene	110251	109960	111923	110711	1059	0.96	111205	110911	110285	110800	470	0.42	-0.08	PASS
Diethylphthalate	69491	69466	70612	69856	655	0.94	69643	69600	69211	69485	238	0.34	0.53	PASS
Diphenylamine	111745	111660	113656	112354	1129	1.00	111794	111456	110898	111383	452	0.41	0.86	PASS
Benzophenone	109118	109040	110942	109700	1076	0.98	109062	108820	108313	108732	382	0.35	0.88	PASS
Methyl Palmitate (C16:0)	96476	96237	98212	96975	1078	1.11	95881	96282	96213	96125	214	0.22	0.88	PASS
Di-n-butylphthalate	80483	80113	81583	80726	765	0.95	81953	82474	82498	82308	308	0.37	-1.96	PASS
n-Eicosane (C20)	121526	121003	123423	121984	1273	1.04	121311	121938	121891	121713	349	0.29	0.22	PASS
Methyl Oleate (C18:1 cis 9)	100764	100356	102155	101092	943	0.93	100255	101047	101315	100872	551	0.55	0.22	PASS
4,4'-Diaminodiphenylmethane	97674	98082	99744	98500	1096	1.11	99963	99965	99759	99896	118	0.12	-1.42	PASS
Ethyl octadecanoate	100715	100155	101939	100936	912	0.90	99674	100558	100769	100334	581	0.58	0.60	PASS
Bis(2-ethylhexyl)adipate	90953	90485	91855	91098	696	0.76	90376	90767	90749	90631	221	0.24	0.51	PASS
Chrysene	128575	128152	129739	128822	822	0.64	129018	128928	128063	128670	527	0.41	0.12	PASS
Bis(2-ethylhexyl) phthalate	95111	94384	96012	95169	816	0.86	95326	95288	94815	95143	285	0.30	0.03	PASS
n-Octacosane (C28)	126681	126100	127877	126886	906	0.71	126641	126055	125499	126065	571	0.45	0.65	PASS
Indeno(1,2,3-cd)pyrene	128061	127999	129920	128660	1092	0.85	133474	133571	132613	133219	527	0.40	-3.54	PASS
