# Supplementary material for: Challenging the Database: Day-of-Analysis Calibration and UF Modeling for Reliable RRF Use in Medical Device Chemical Characterization
Source: Anal Chem. 2025 Oct 8;97(41):22719–29. doi: 10.1021/acs.analchem.5c04247 (PMC12547855; doi:10.1021/acs.analchem.5c04247)
Supplement: Supplementary file 2 [file ac5c04247_si_002.zip › Tetra(Ethylene glycol) diacrylate 398802 Lot # MKCQ8235 no exp sigma aldrich.pdf]

3050 Spruce Street, Saint Louis, MO 63103, USA
Website: www.sigmaaldrich.com
Email USA: techserv@sial.com
Outside USA: eurtechserv@sial.com

Certificate of Analysis

Product Name:
Tetra(ethylene glycol) diacrylate - technical grade, contains 150-200 ppm MEHQ as inhibitor, 100-150 ppm HQ as inhibitor

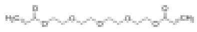

**Product Number:**	**398802**
**Batch Number:**	**MKCQ8235**
Brand:	ALDRICH
CAS Number:	17831-71-9
MDL Number:	MFCD00008630
Formula:	$C_{14}H_{22}O_7$
Formula Weight:	302.32 g/mol
Quality Release Date:	10 DEC 2021

Test	Specification	Result
Appearance (Color)	Colorless to Yellow	Colorless
Appearance (Form)	Liquid	Liquid
Refractive index at 20 ° C	1.461 - 1.469	1.466
Infrared Spectrum	Conforms to Structure	Conforms
Purity (GC)	$\geq$ 87.0 %	90.9 %
HQ as Inhibitor	100 - 150 ppm	105 ppm
MEHQ as Inhibitor	150 - 200 ppm	161 ppm

Larry Coers, Director
Quality Control
Milwaukee, WI US

Sigma-Aldrich warrants, that at the time of the quality release or subsequent retest date this product conformed to the information contained in this publication. The current Specification sheet may be available at Sigma-Aldrich.com. For further inquiries, please contact Technical Service. Purchaser must determine the suitability of the product for its particular use. See reverse side of invoice or packing slip for additional terms and conditions of sale.
