# Supplementary material for: Challenging the Database: Day-of-Analysis Calibration and UF Modeling for Reliable RRF Use in Medical Device Chemical Characterization
Source: Anal Chem. 2025 Oct 8;97(41):22719–29. doi: 10.1021/acs.analchem.5c04247 (PMC12547855; doi:10.1021/acs.analchem.5c04247)
Supplement: Supplementary file 2 [file ac5c04247_si_002.zip › TJ1886.pdf]

Certificate of Analysis

**Product Name:**	Palmitic acid-2,2-d_2
**Product Description:**	98 atom % D
**Product Brand:**	Sigma-Aldrich
**Product Number:**	489662
**Molecular Weight:**	258.44
**Molecular Formula:**	$CH_3(CH_2)_{13}CD_2CO_2H$
**CAS Number:**	62689-96-7

TEST	SPECIFICATION	LOT TJ1886 RESULTS
**% Water: < = 0.5%**		0.04%
**GC: Minimum 99% chemical purity**		99.8%
**1H-NMR (FT): Structure and purity verification**		Meets requirement
**1H-NMR (FT): Minimum 98 atom% D**		99.0 atom% D
**m.p.: Conforms to standard**		61.9-63.0oC
**Appearance: White powder**		White powder
**Release Date:**		1996/10/21

C. T. Tan, PhD, Scientist
Quality Control Laboratory
Isotec Stable Isotopes (Sigma-Aldrich)
Miamisburg, Ohio, USA

© 2022 Merck KGaA, Darmstadt, Germany and/or its affiliates. All Rights Reserved.
Reproduction of any materials from the site is strictly forbidden without permission.
Site Use Terms | Privacy Policy | General Terms and Conditions of Sale | Copyright Consent | Cookie Settings
