# Supplementary material for: Challenging the Database: Day-of-Analysis Calibration and UF Modeling for Reliable RRF Use in Medical Device Chemical Characterization
Source: Anal Chem. 2025 Oct 8;97(41):22719–29. doi: 10.1021/acs.analchem.5c04247 (PMC12547855; doi:10.1021/acs.analchem.5c04247)
Supplement: Supplementary file 2 [file ac5c04247_si_002.zip › Triphenyl phosphate 241288 Lot # BCCC4994 No exp sigma aldrich.pdf]

SIGMA-ALDRICH

3050 Spruce Street, Saint Louis, MO 63103 USA
Email USA: techserv@sial.com Outside USA: eurtechserv@sial.com

Certificate of Analysis

**Product Name:**	Triphenyl phosphate<br>>= 99 %
**Product Number:**	241288
**Batch Number:**	BCCC4994
**Brand:**	Aldrich
**CAS Number:**	115-86-6
**Formula:**	$(C_6H_5O)_3PO$
**Formula Weight:**	326.28
**Quality Release Date:**	29 NOV 2019

TEST	SPECIFICATION	RESULT
**APPEARANCE (COLOR)**	WHITE	WHITE
**APPEARANCE (FORM)**	FLAKES AND CHIPS	FLAKES
**PURITY (GC AREA %)**	≥ 99.0 %	99.8 %
**INFRARED SPECTRUM**	CONFORMS TO STRUCTURE	CONFORMS

Dr. Reinhold Schwenninger
Quality Assurance
Buchs, Switzerland

Sigma-Aldrich warrants that at the time of the quality release or subsequent retest date this product conformed to the information contained in this publication. The current specification sheet may be available at Sigma-Aldrich.com. For further inquiries, please contact Technical Service. Purchaser must determine the suitability of the product for its particular use. See reverse side of invoice or packing slip for additional terms and conditions of sale.
