# Supplementary material for: Challenging the Database: Day-of-Analysis Calibration and UF Modeling for Reliable RRF Use in Medical Device Chemical Characterization
Source: Anal Chem. 2025 Oct 8;97(41):22719–29. doi: 10.1021/acs.analchem.5c04247 (PMC12547855; doi:10.1021/acs.analchem.5c04247)
Supplement: Supplementary file 2 [file ac5c04247_si_002.zip › Triphenyl phosphate-d15 615218 Lot # TA2534V sigma aldrich.pdf]

3050 Spruce Street, Saint Louis, MO 63103, USA
Website: www.sigmaaldrich.com
Email USA: techserv@sial.com
Outside USA: eurtechserv@sial.com

Certificate of Analysis

Product Name:
Triphenyl phosphate-d15 - 98 atom % D

Product Number: **615218**
Batch Number: **TA2534V**
Brand: ALDRICH
Formula: C18D15O4P
Formula Weight: 341.08 g/mol
Quality Release Date: 23 FEB 2022

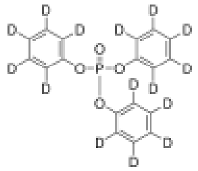

Test	Specification	Result
Appearance (Color)	White	White
Appearance (Form)	Powder	Powder
Water (by Karl Fischer)	$\leq$ 0.5 %	0.2 %
Purity (HPLC)	$\geq$ 99.0 %	99.8 %
1H NMR Spectrum	Conforms to Structure	Conforms
Deuterium NMR Spectrum	Conforms to Structure	Conforms
D Enrichment	$\geq$ 98.0 atom %	99.3 atom %

Laura E. Baird, Manager
Quality Assurance & Control
Miamisburg, Ohio US

Sigma-Aldrich warrants, that at the time of the quality release or subsequent retest date this product conformed to the information contained in this publication. The current Specification sheet may be available at Sigma-Aldrich.com. For further inquiries, please contact Technical Service. Purchaser must determine the suitability of the product for its particular use. See reverse side of invoice or packing slip for additional terms and conditions of sale.
