# Supplementary figures and images for: Challenging the Database: Day-of-Analysis Calibration and UF Modeling for Reliable RRF Use in Medical Device Chemical Characterization
Source: Anal Chem. 2025 Oct 8;97(41):22719–29. doi: 10.1021/acs.analchem.5c04247 (PMC12547855; doi:10.1021/acs.analchem.5c04247)

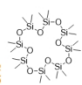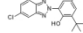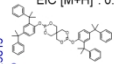

316x102mm (150 x 150 DPI)

Supplement: Supplementary file 3 [file ac5c04247_si_003.pdf]

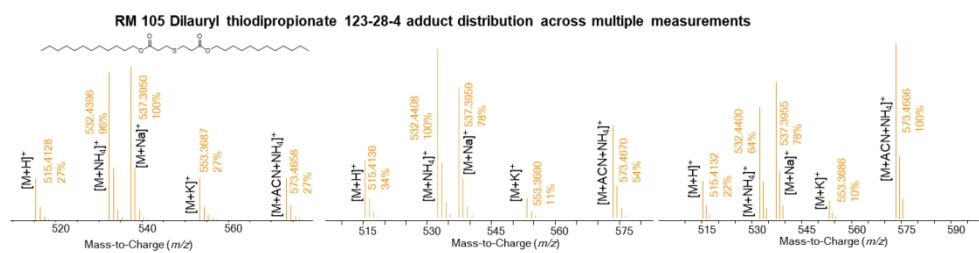

256x71mm (150 x 150 DPI)

Supplement: Supplementary file 4 [file ac5c04247_si_004.pdf]

Change in RRF with Concentration (1-Dodecene)

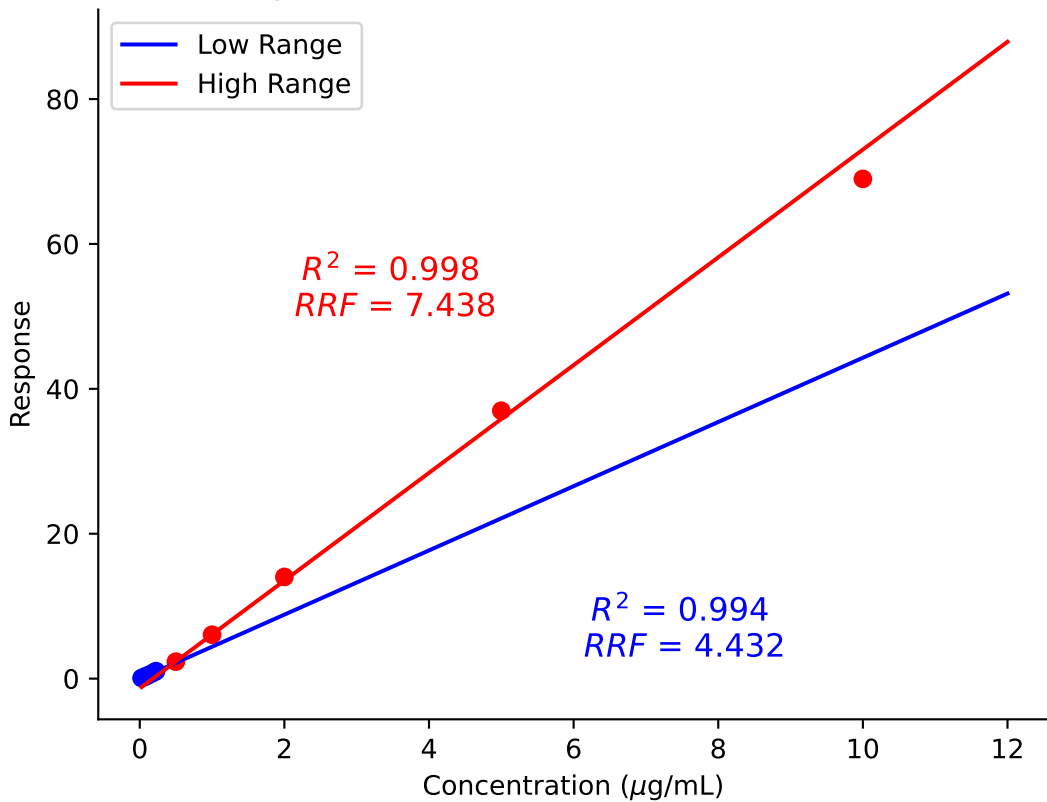

Supplement: Supplementary file 5 [file ac5c04247_si_005.zip › 1-Dodecene-SVOC-EWandHigh.pdf]

# Change in RRF with Concentration (1-Dodecene)

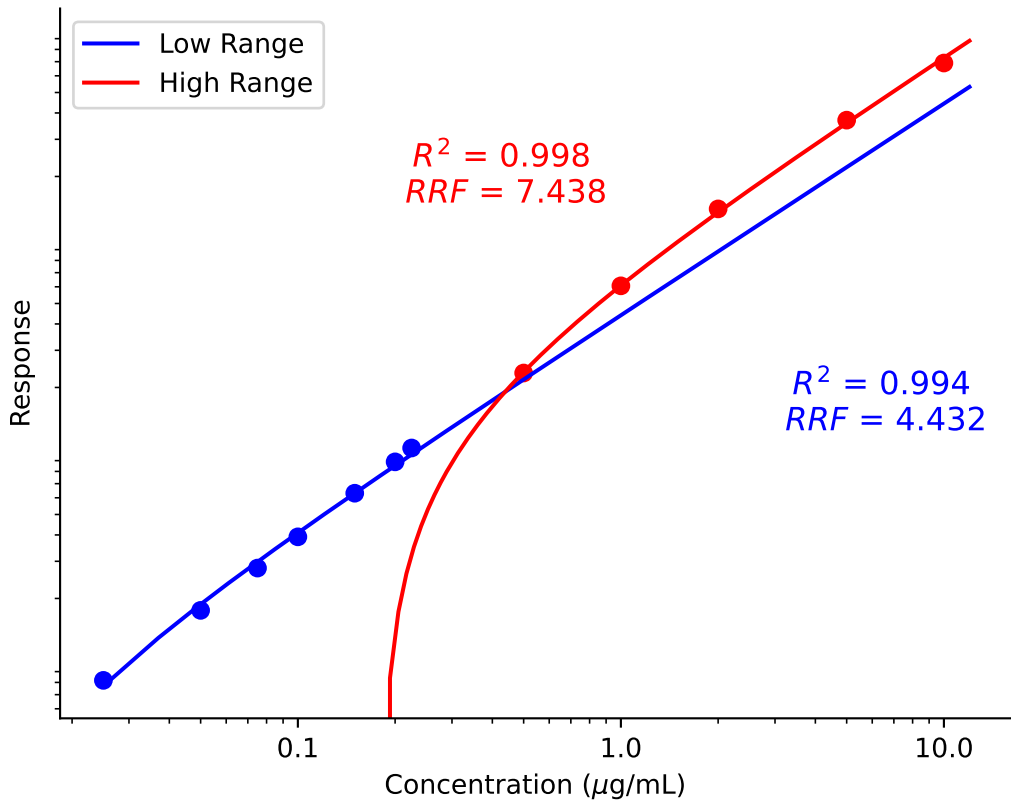

Supplement: Supplementary file 5 [file ac5c04247_si_005.zip › 1-Dodecene-SVOCloglog-EWandHigh.pdf]

Change in RRF with Concentration (1-Dodecene)

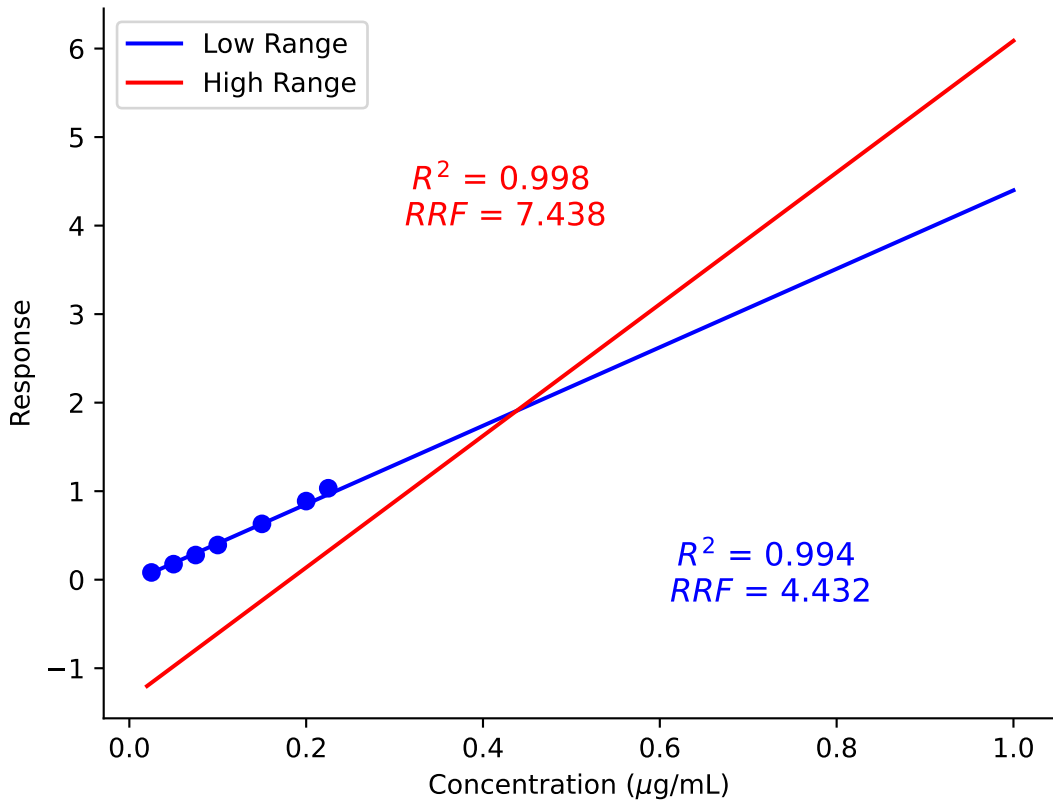

Supplement: Supplementary file 5 [file ac5c04247_si_005.zip › 1-Dodecene-SVOC-LowView.pdf]

# Change in RRF with Concentration (12-Aminododecanolactam)

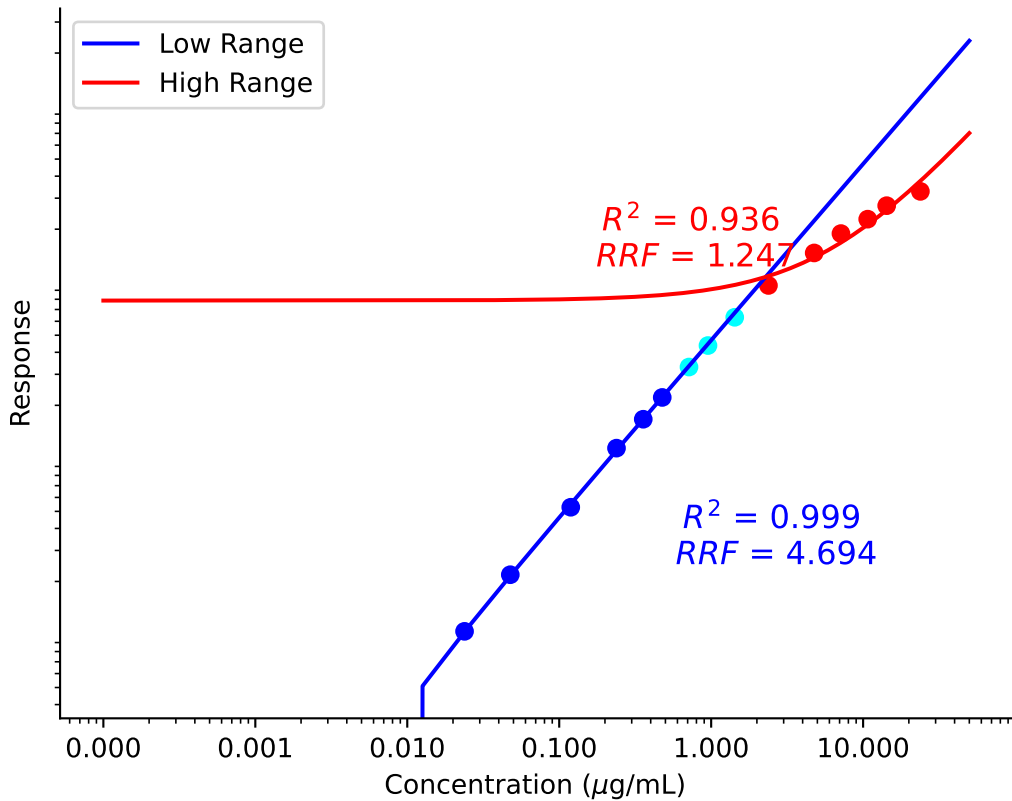

Supplement: Supplementary file 5 [file ac5c04247_si_005.zip › 12-Aminododecanolactam-NVOCloglog-EWandHigh.pdf]

# Change in RRF with Concentration (Acenaphthylene)

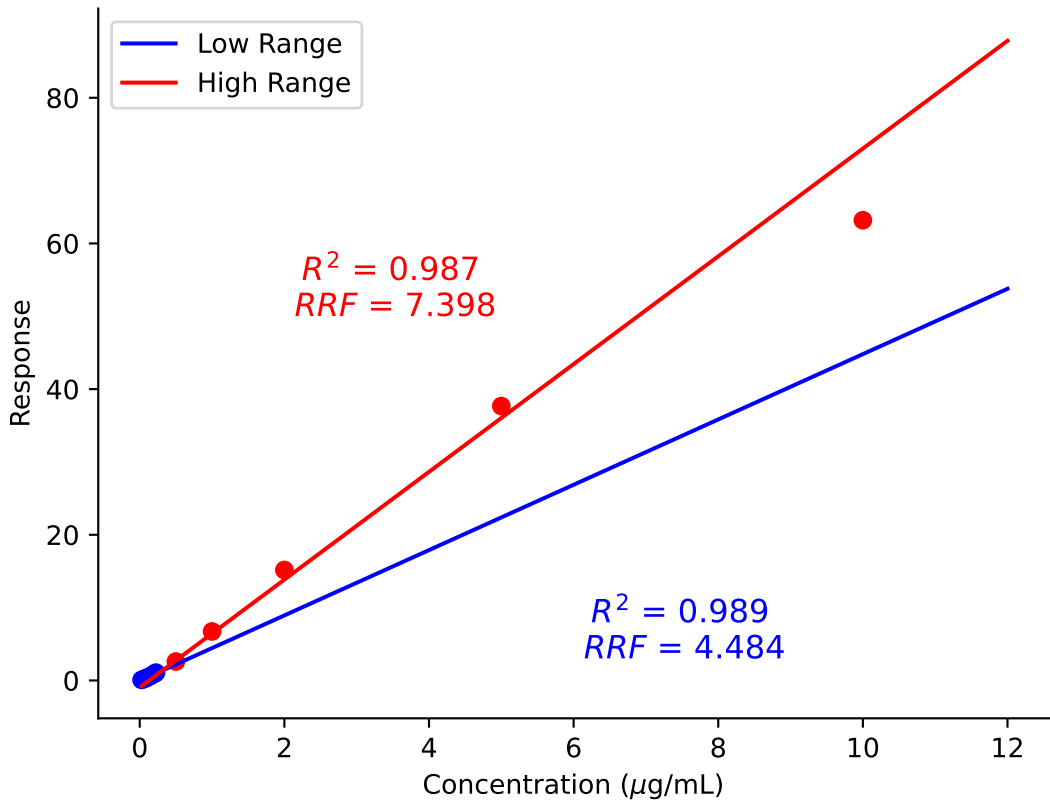

Supplement: Supplementary file 5 [file ac5c04247_si_005.zip › Acenaphthylene-SVOC-EWandHigh.pdf]

# Change in RRF with Concentration (Acenaphthylene)

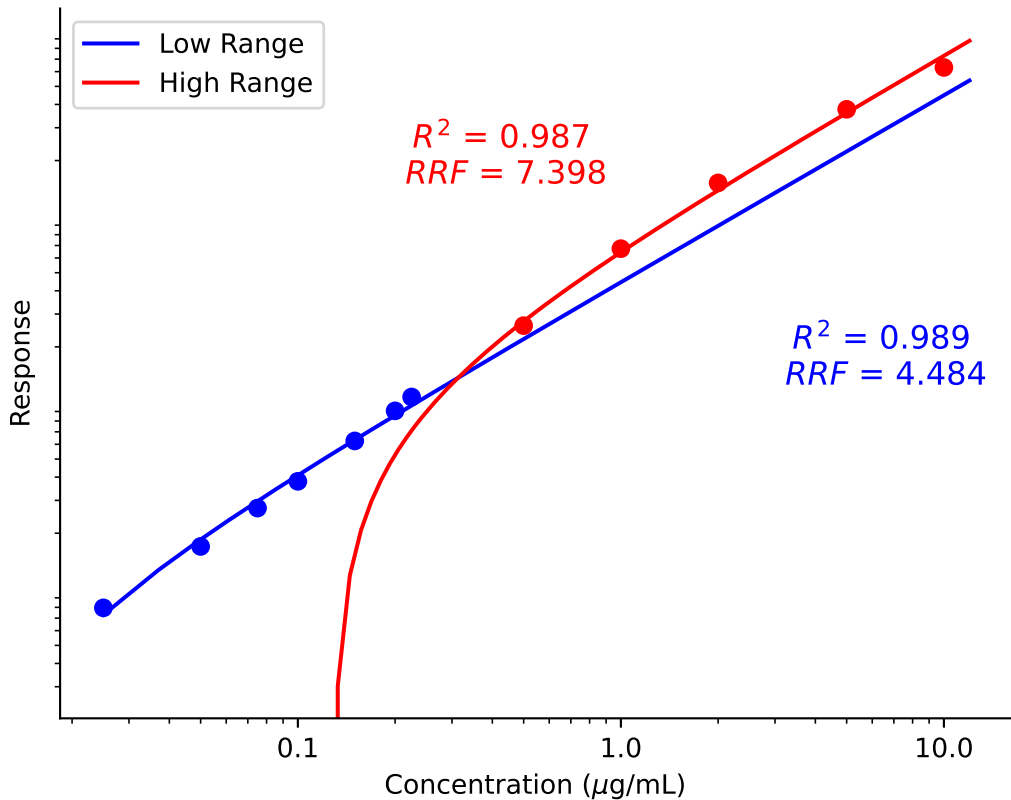

Supplement: Supplementary file 5 [file ac5c04247_si_005.zip › Acenaphthylene-SVOCloglog-EWandHigh.pdf]

# Change in RRF with Concentration (Acenaphthylene)

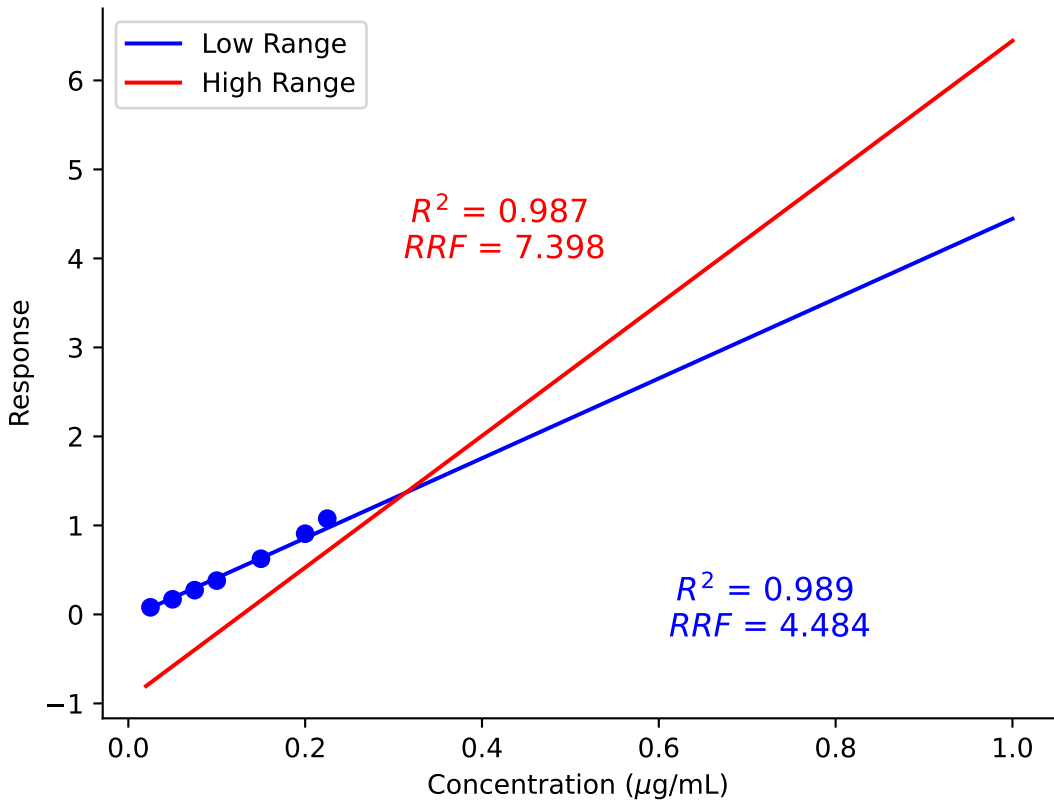

Supplement: Supplementary file 5 [file ac5c04247_si_005.zip › Acenaphthylene-SVOC-LowView.pdf]

# Change in RRF with Concentration (Acetophenone)

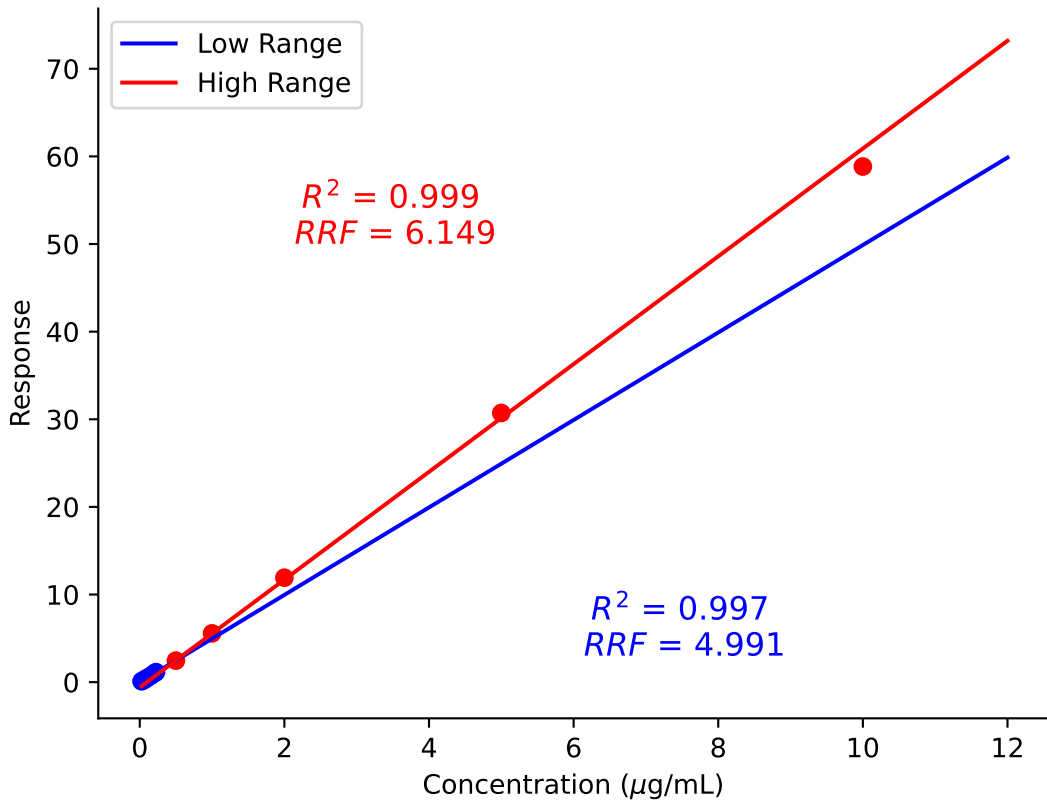

Supplement: Supplementary file 5 [file ac5c04247_si_005.zip › Acetophenone-SVOC-EWandHigh.pdf]

# Change in RRF with Concentration (Acetophenone)

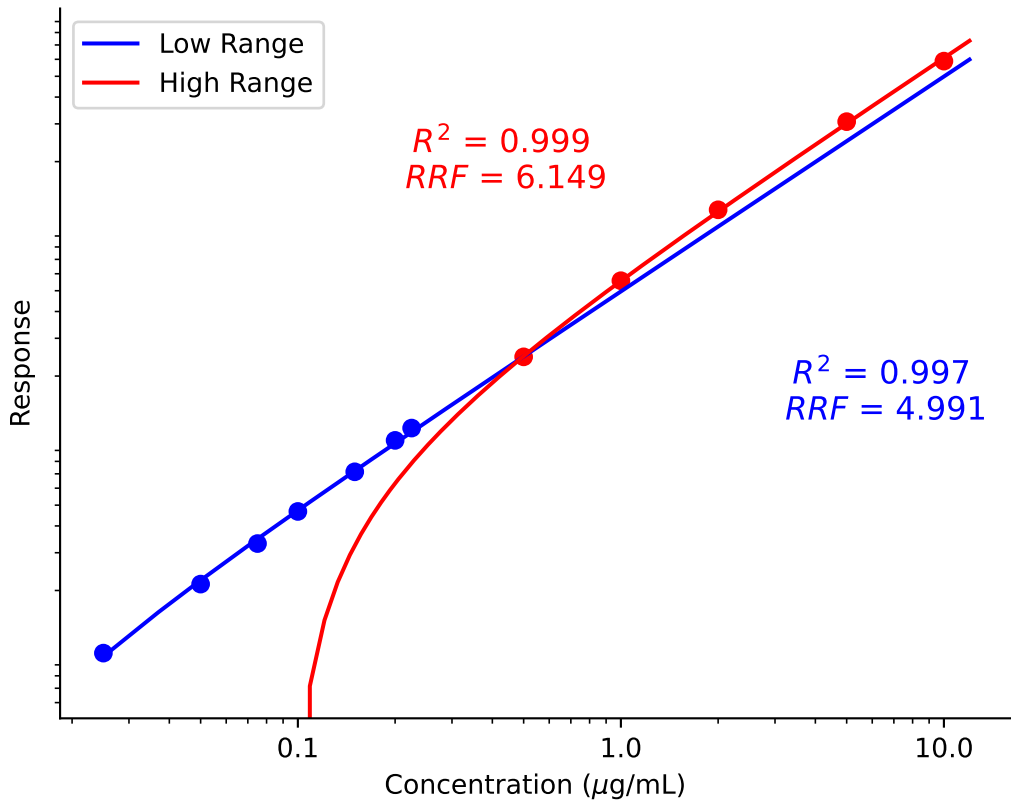

Supplement: Supplementary file 5 [file ac5c04247_si_005.zip › Acetophenone-SVOCloglog-EWandHigh.pdf]

# Change in RRF with Concentration (Acetophenone)

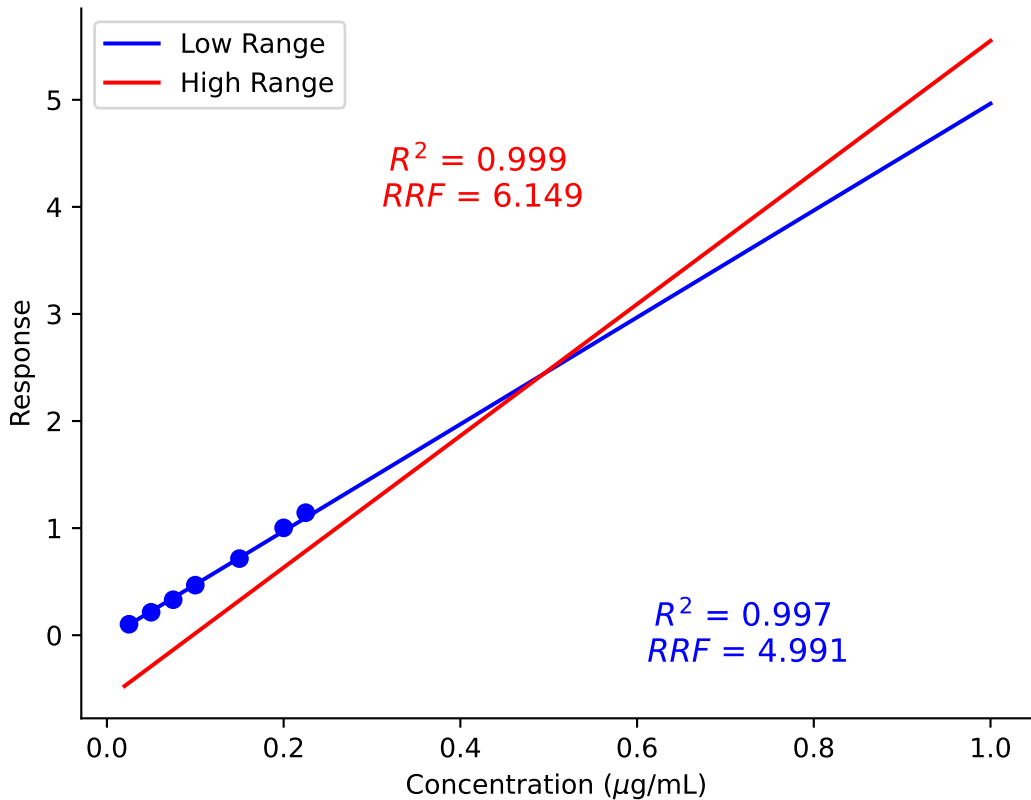

Supplement: Supplementary file 5 [file ac5c04247_si_005.zip › Acetophenone-SVOC-LowView.pdf]

# Change in RRF with Concentration (Behenic Acid)

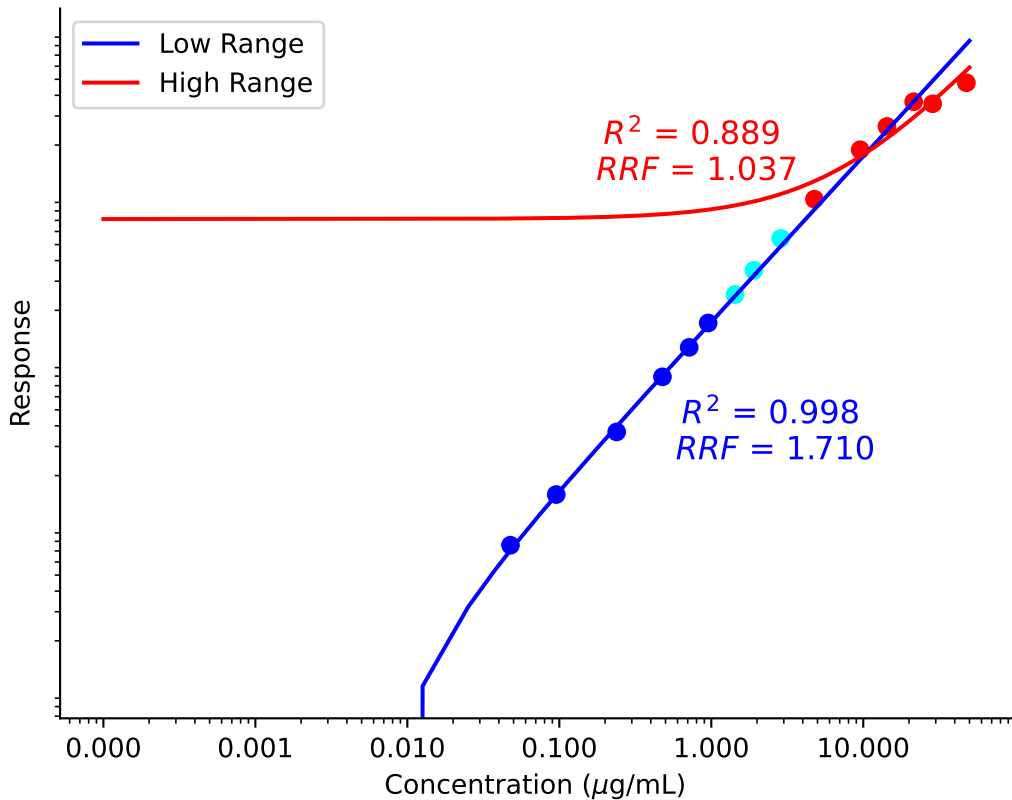

Supplement: Supplementary file 5 [file ac5c04247_si_005.zip › Behenic Acid-NVOCloglog-EWandHigh.pdf]

# Change in RRF with Concentration (Benzophenone)

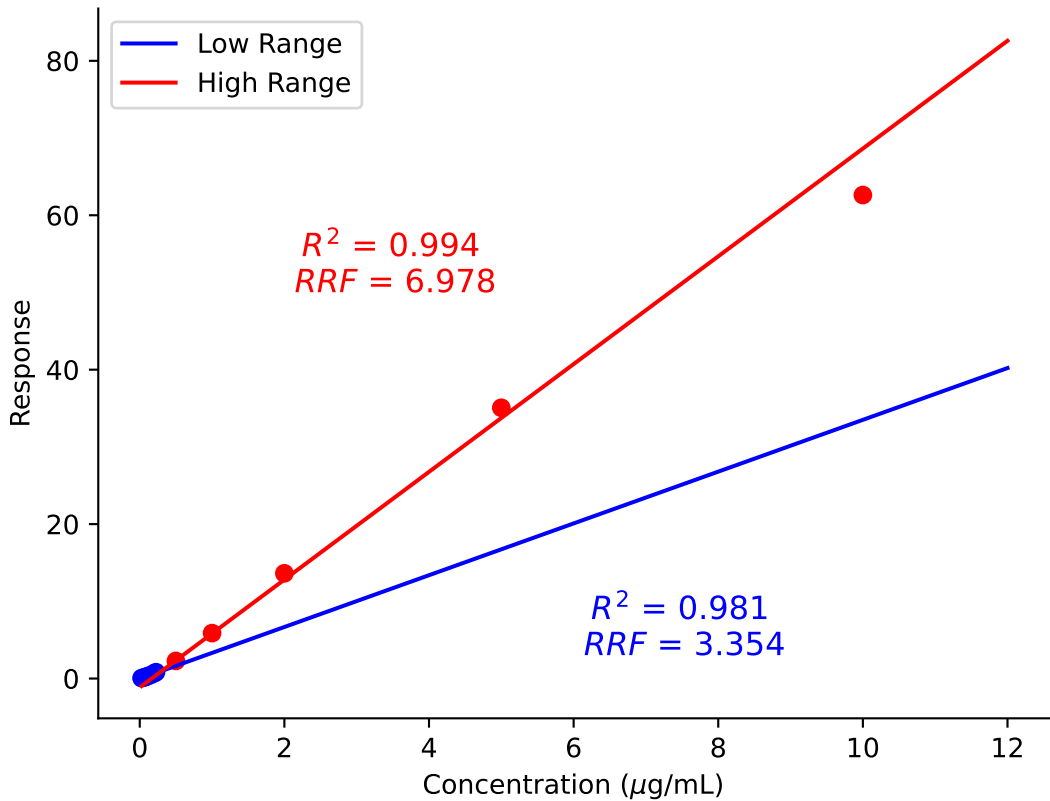

Supplement: Supplementary file 5 [file ac5c04247_si_005.zip › Benzophenone-SVOC-EWandHigh.pdf]

# Change in RRF with Concentration (Benzophenone)

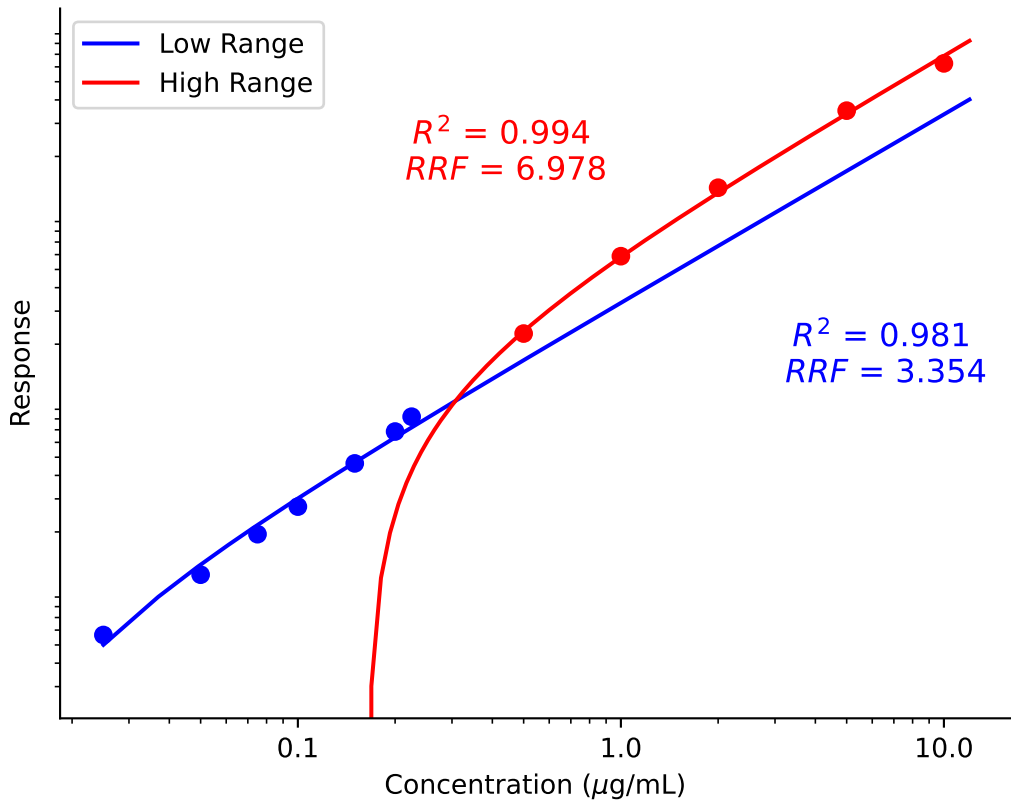

Supplement: Supplementary file 5 [file ac5c04247_si_005.zip › Benzophenone-SVOCloglog-EWandHigh.pdf]

# Change in RRF with Concentration (Benzophenone)

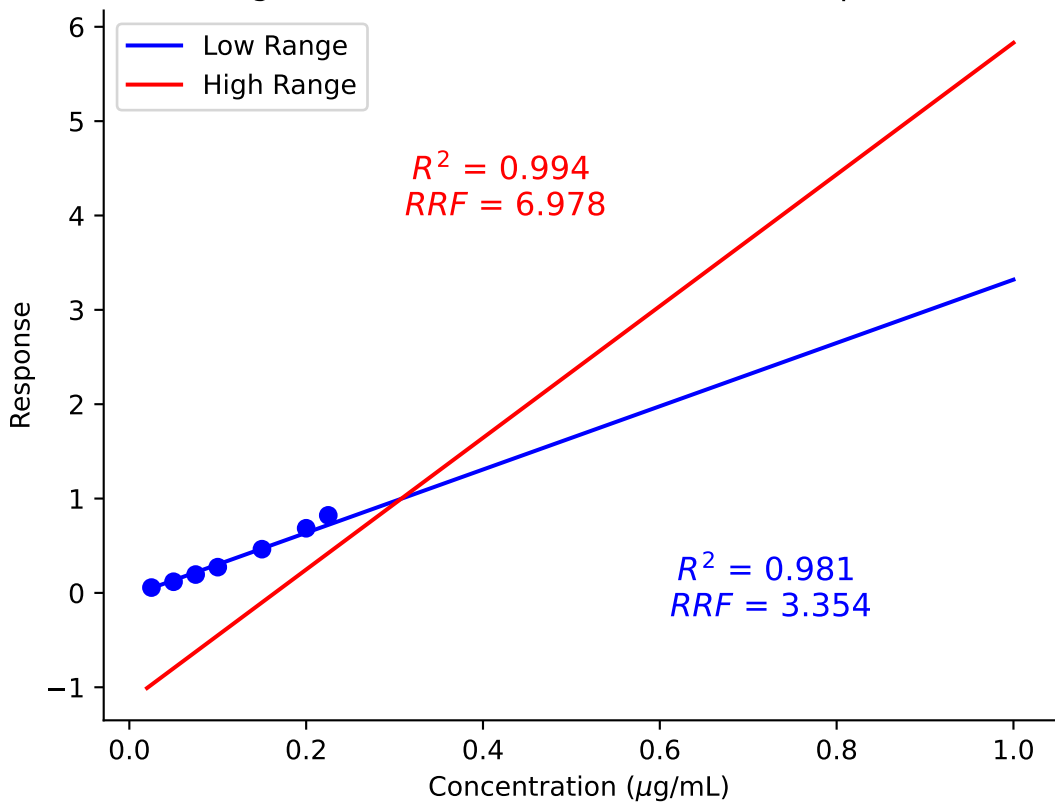

Supplement: Supplementary file 5 [file ac5c04247_si_005.zip › Benzophenone-SVOC-LowView.pdf]

# Change in RRF with Concentration (Bis(2-ethylhexyl) adipate)

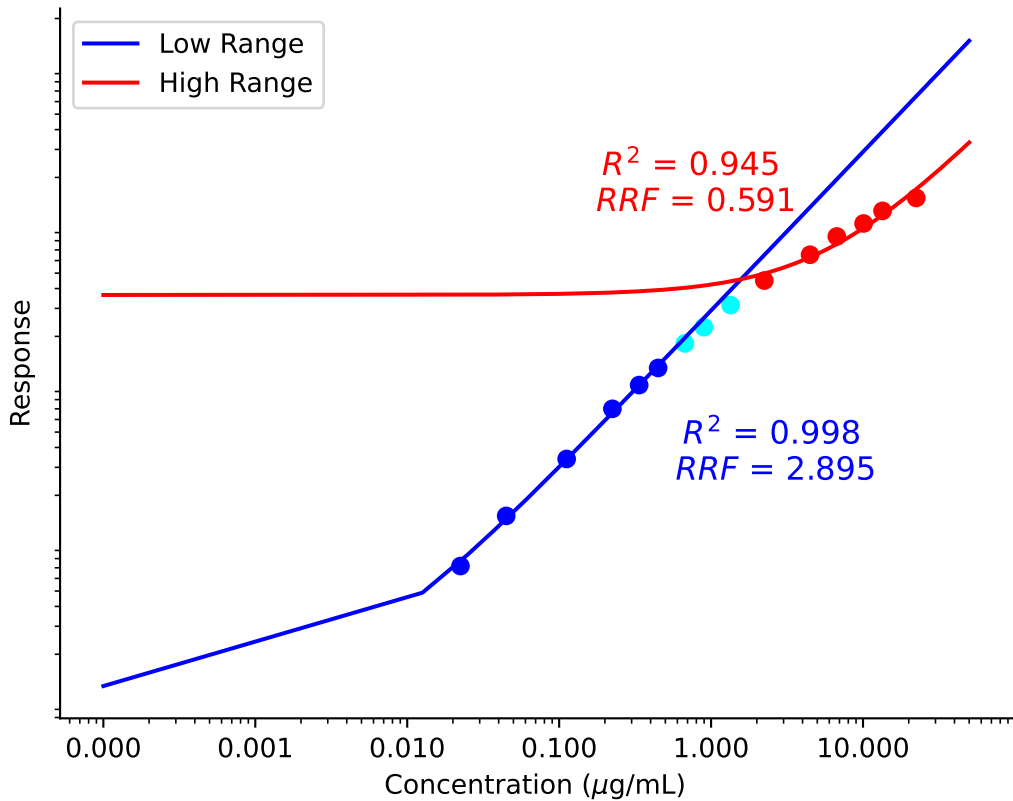

Supplement: Supplementary file 5 [file ac5c04247_si_005.zip › Bis(2-ethylhexyl) adipate-NVOCloglog-EWandHigh.pdf]

Change in RRF with Concentration (Bis(2-ethylhexyl) phthalate)

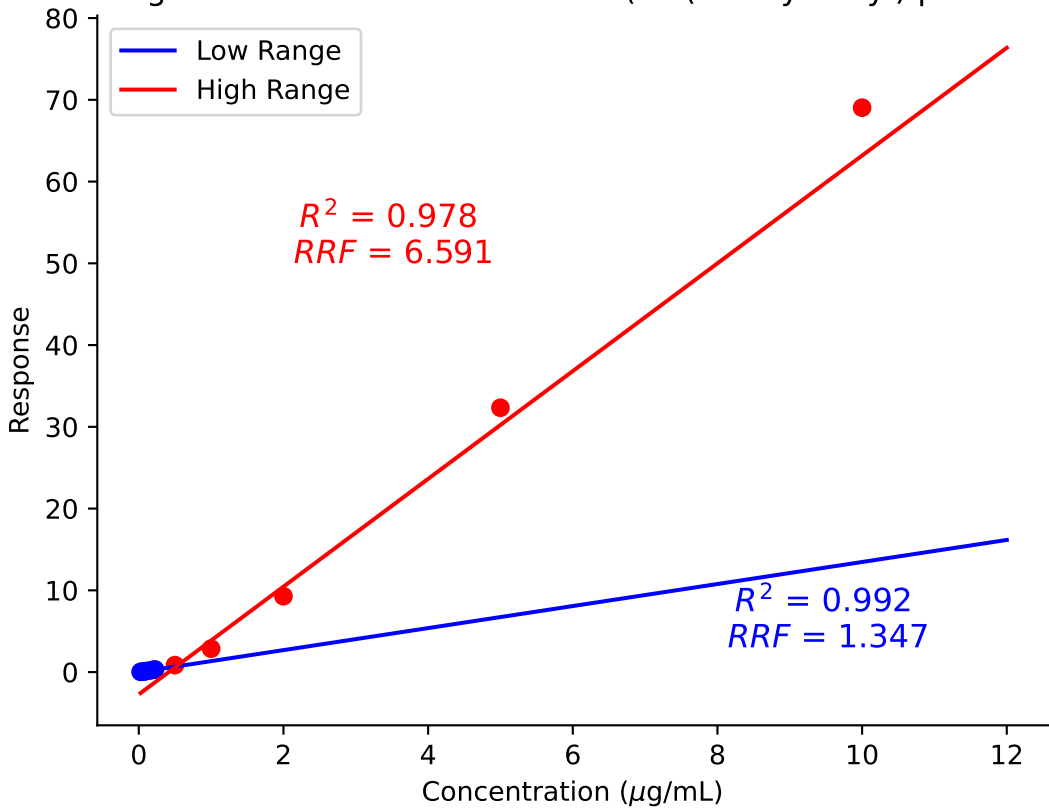

Supplement: Supplementary file 5 [file ac5c04247_si_005.zip › Bis(2-ethylhexyl) phthalate-SVOC-EWandHigh.pdf]

# Change in RRF with Concentration (Bis(2-ethylhexyl) phthalate)

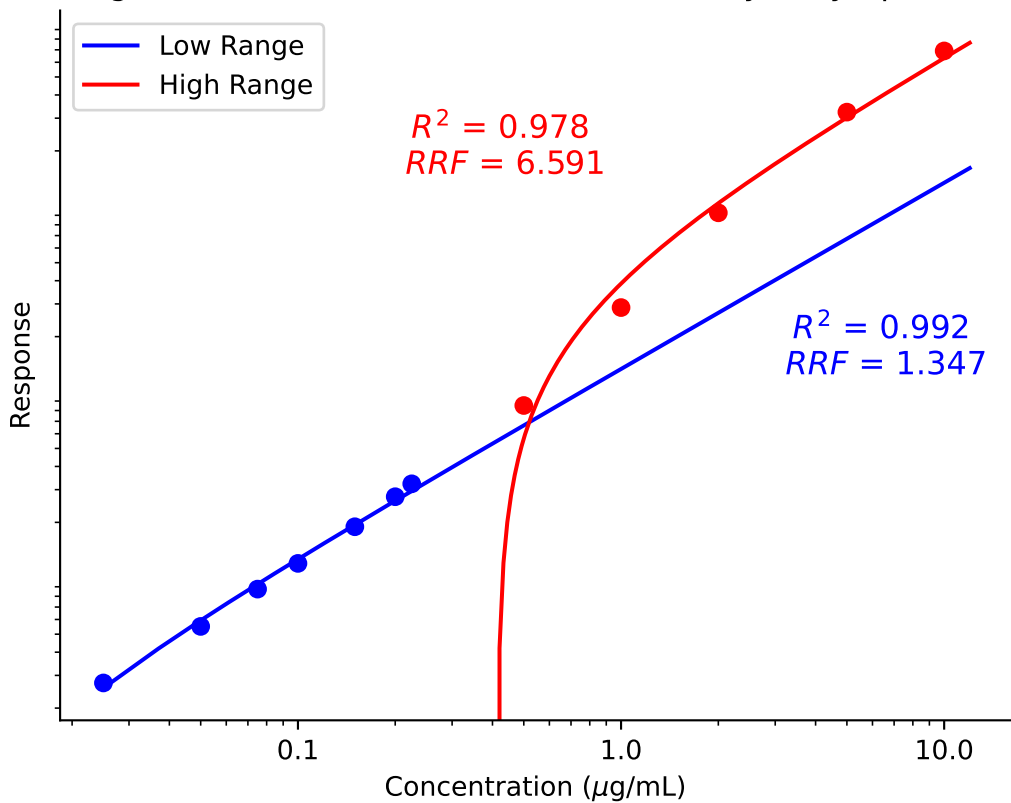

Supplement: Supplementary file 5 [file ac5c04247_si_005.zip › Bis(2-ethylhexyl) phthalate-SVOCloglog-EWandHigh.pdf]

# Change in RRF with Concentration (Bis(2-ethylhexyl) phthalate)

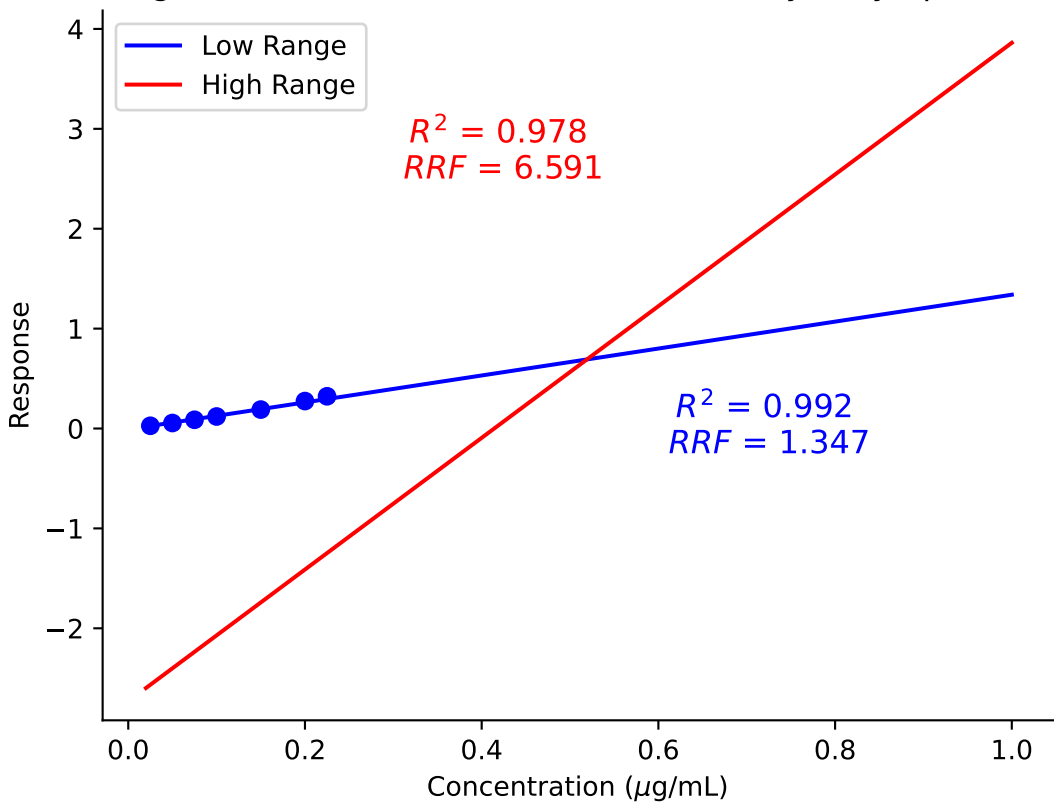

Supplement: Supplementary file 5 [file ac5c04247_si_005.zip › Bis(2-ethylhexyl) phthalate-SVOC-LowView.pdf]

# Change in RRF with Concentration (Butylated Hydroxytoluene)

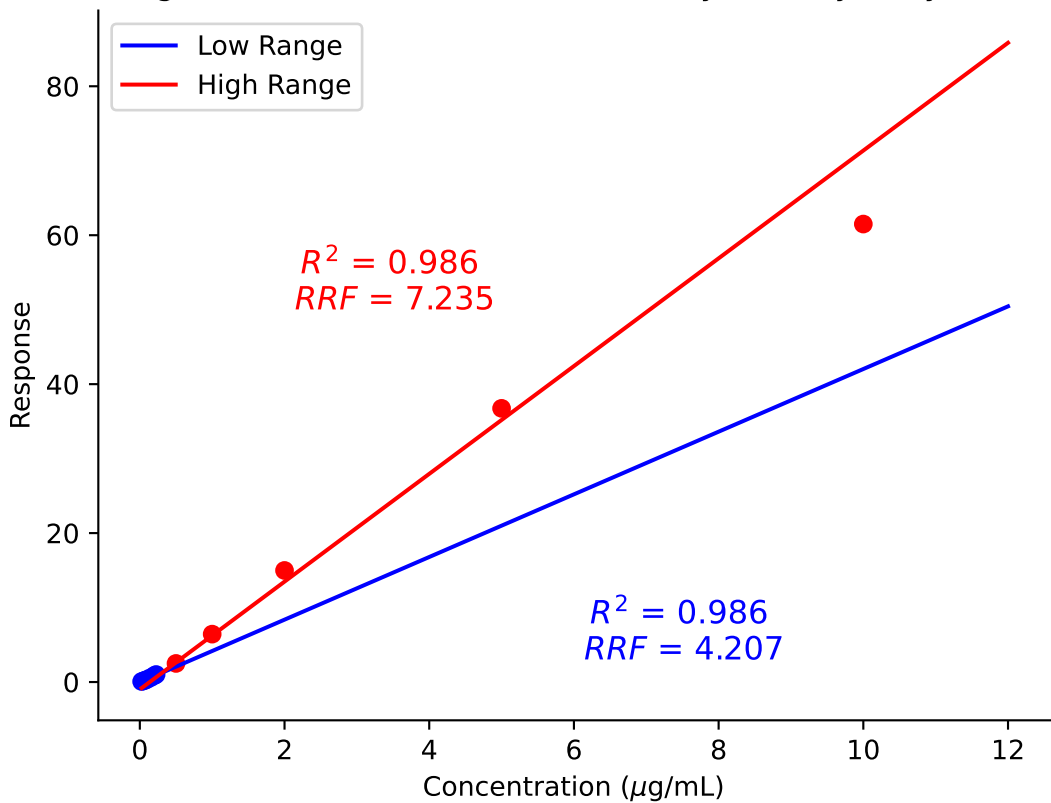

Supplement: Supplementary file 5 [file ac5c04247_si_005.zip › Butylated Hydroxytoluene-SVOC-EWandHigh.pdf]

# Change in RRF with Concentration (Butylated Hydroxytoluene)

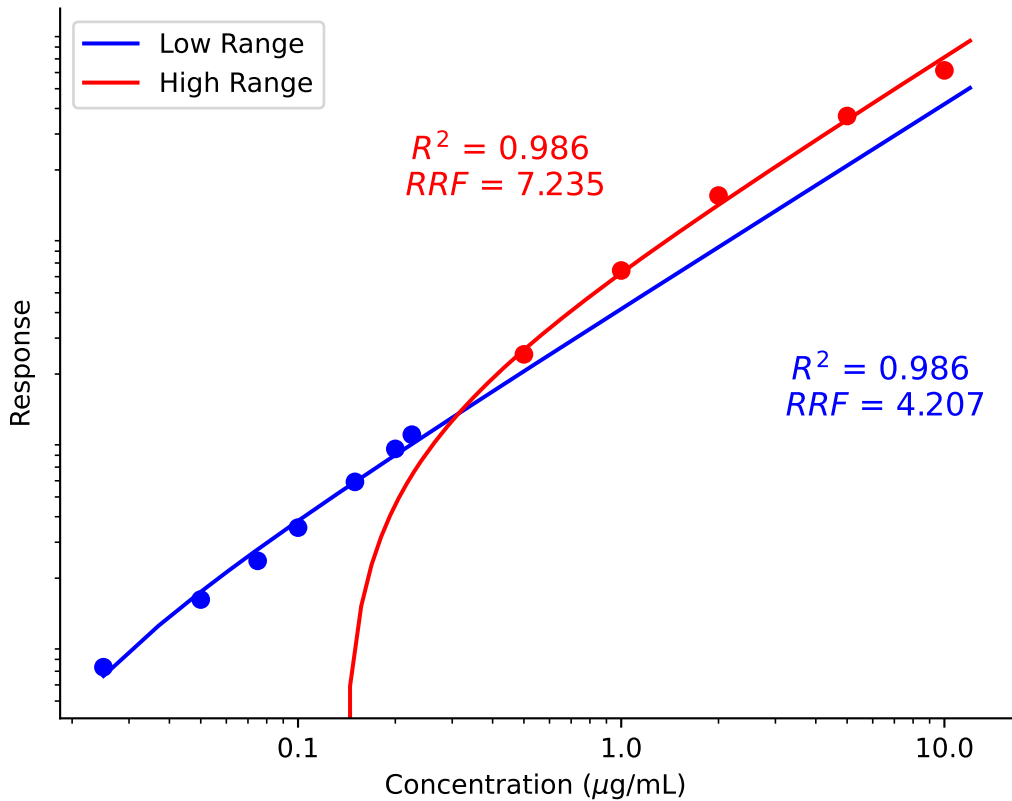

Supplement: Supplementary file 5 [file ac5c04247_si_005.zip › Butylated Hydroxytoluene-SVOCloglog-EWandHigh.pdf]

Change in RRF with Concentration (Butylated Hydroxytoluene)

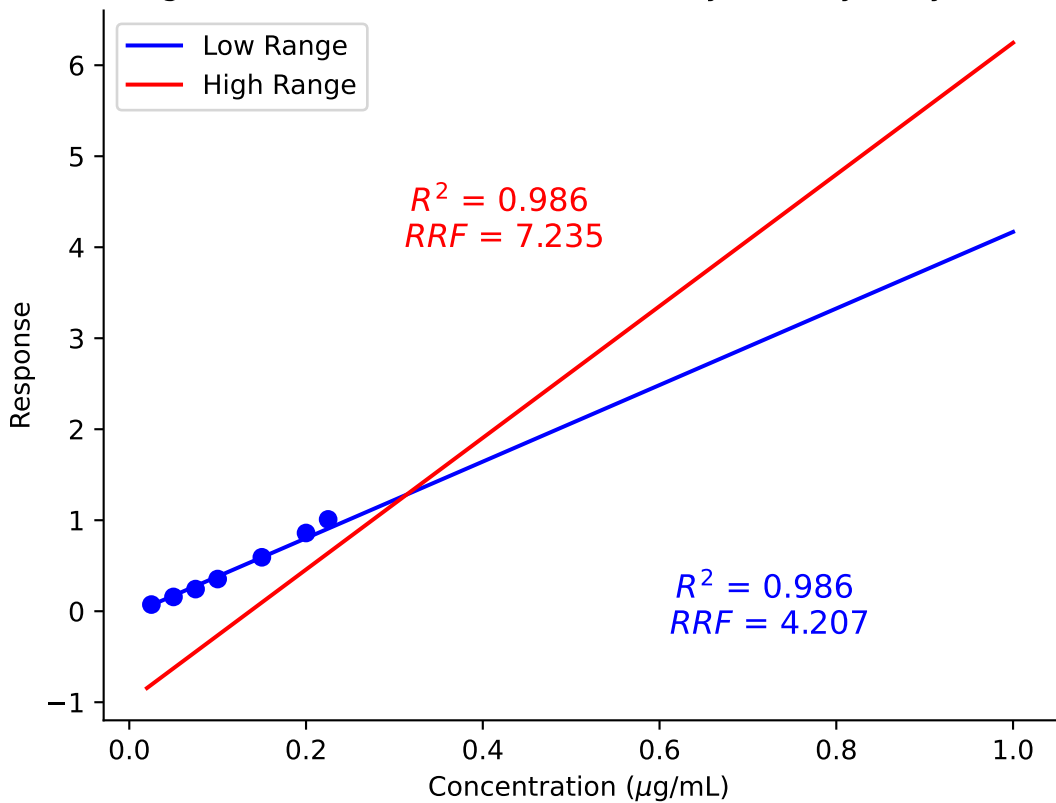

Supplement: Supplementary file 5 [file ac5c04247_si_005.zip › Butylated Hydroxytoluene-SVOC-LowView.pdf]

Change in RRF with Concentration (Caprolactam)

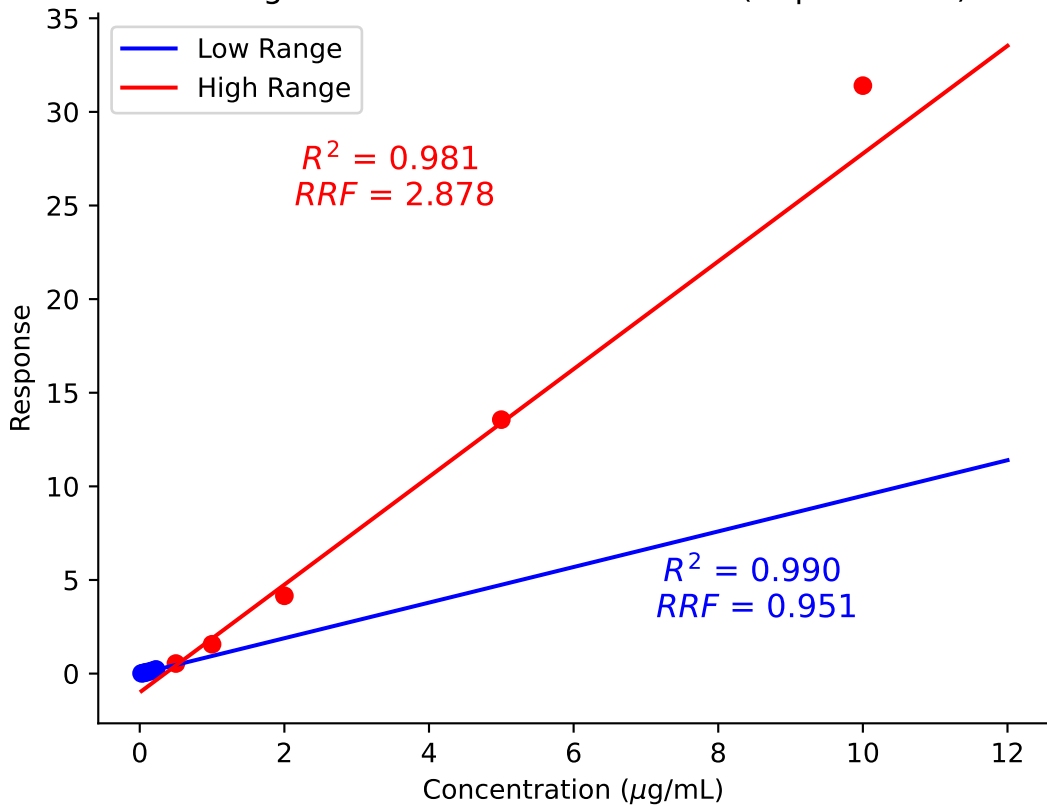

Supplement: Supplementary file 5 [file ac5c04247_si_005.zip › Caprolactam-SVOC-EWandHigh.pdf]

# Change in RRF with Concentration (Caprolactam)

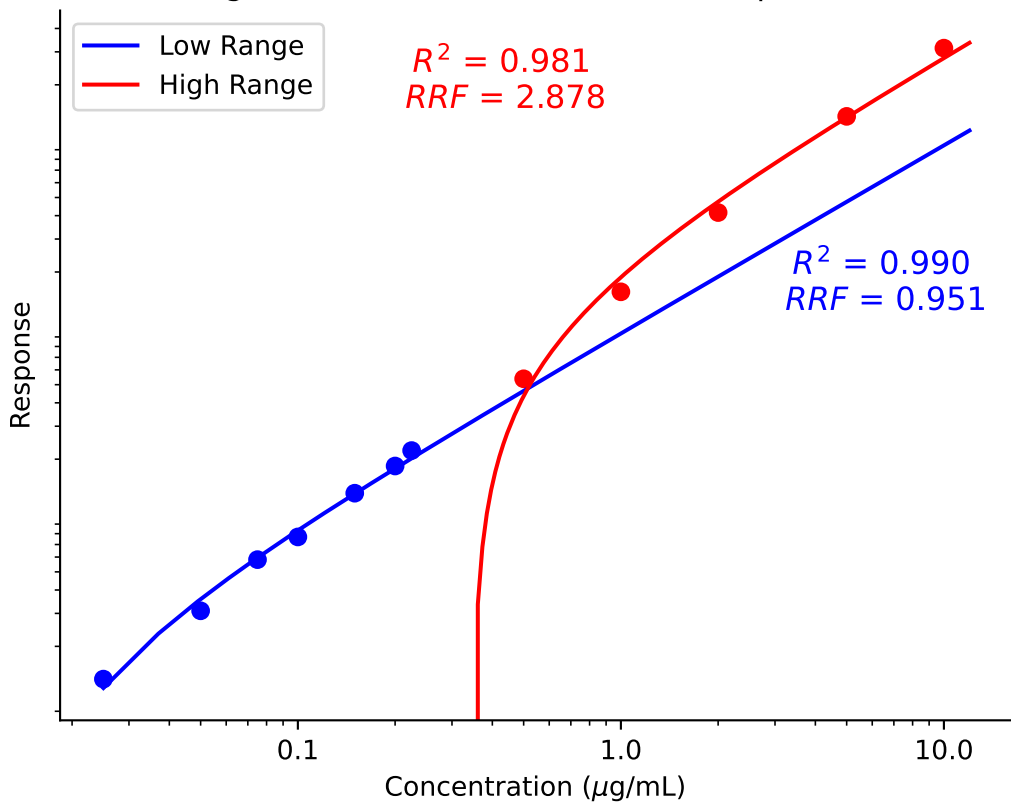

Supplement: Supplementary file 5 [file ac5c04247_si_005.zip › Caprolactam-SVOCloglog-EWandHigh.pdf]

Change in RRF with Concentration (Caprolactam)

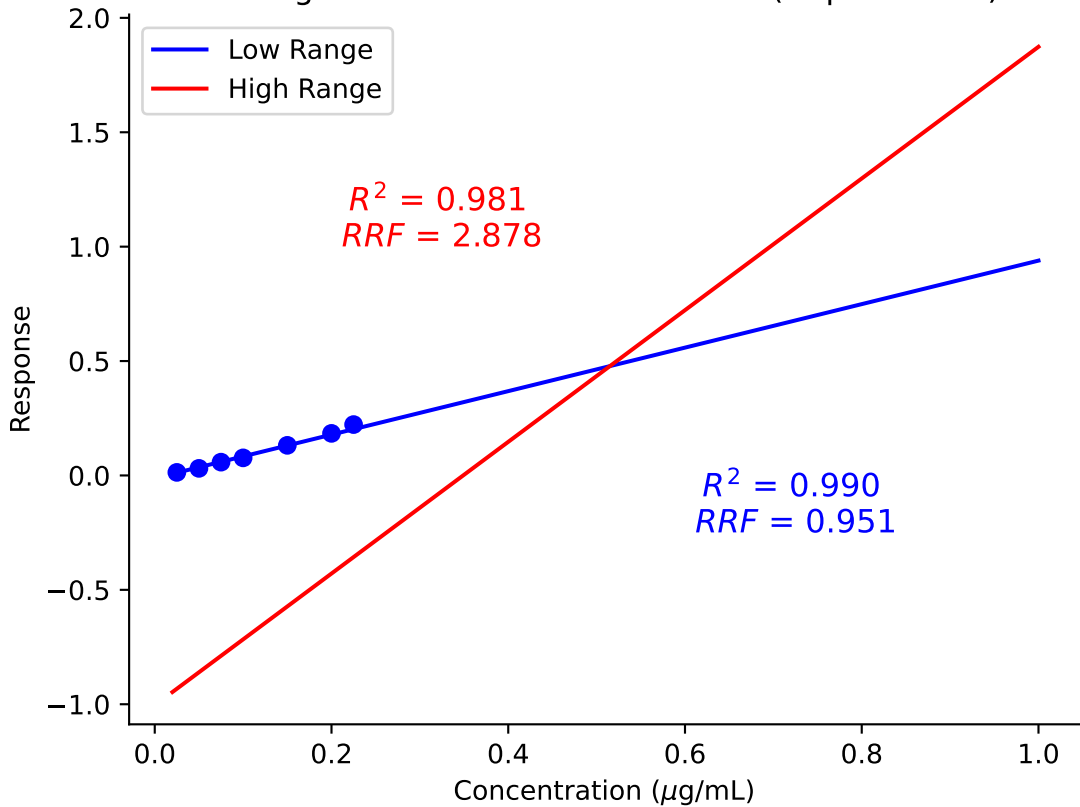

Supplement: Supplementary file 5 [file ac5c04247_si_005.zip › Caprolactam-SVOC-LowView.pdf]

Change in RRF with Concentration (Cetene)

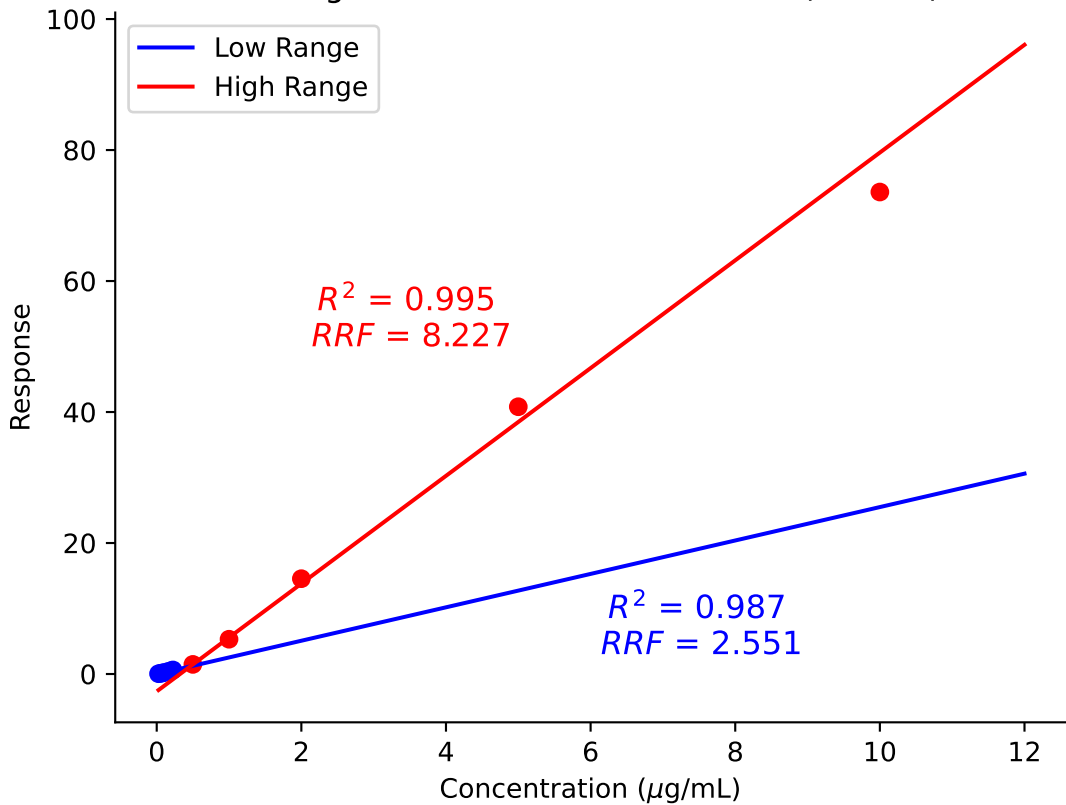

Supplement: Supplementary file 5 [file ac5c04247_si_005.zip › Cetene-SVOC-EWandHigh.pdf]

# Change in RRF with Concentration (Cetene)

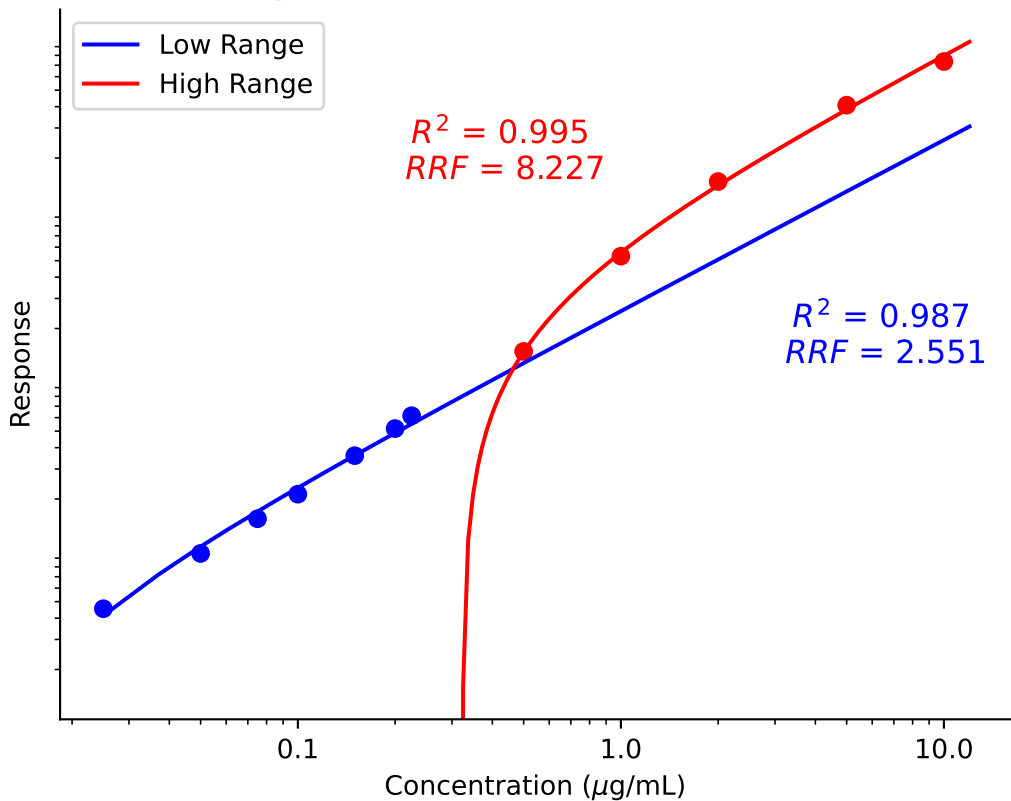

Supplement: Supplementary file 5 [file ac5c04247_si_005.zip › Cetene-SVOCloglog-EWandHigh.pdf]

Change in RRF with Concentration (Cetene)

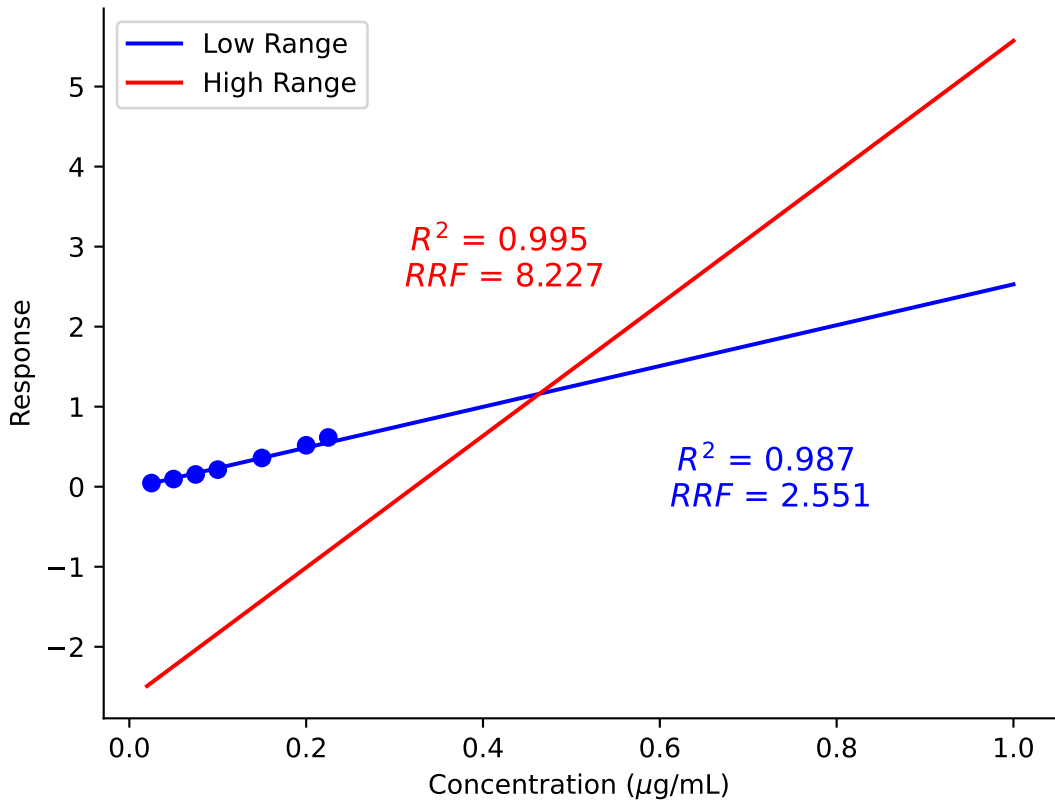

Supplement: Supplementary file 5 [file ac5c04247_si_005.zip › Cetene-SVOC-LowView.pdf]

Change in RRF with Concentration (Chrysene)

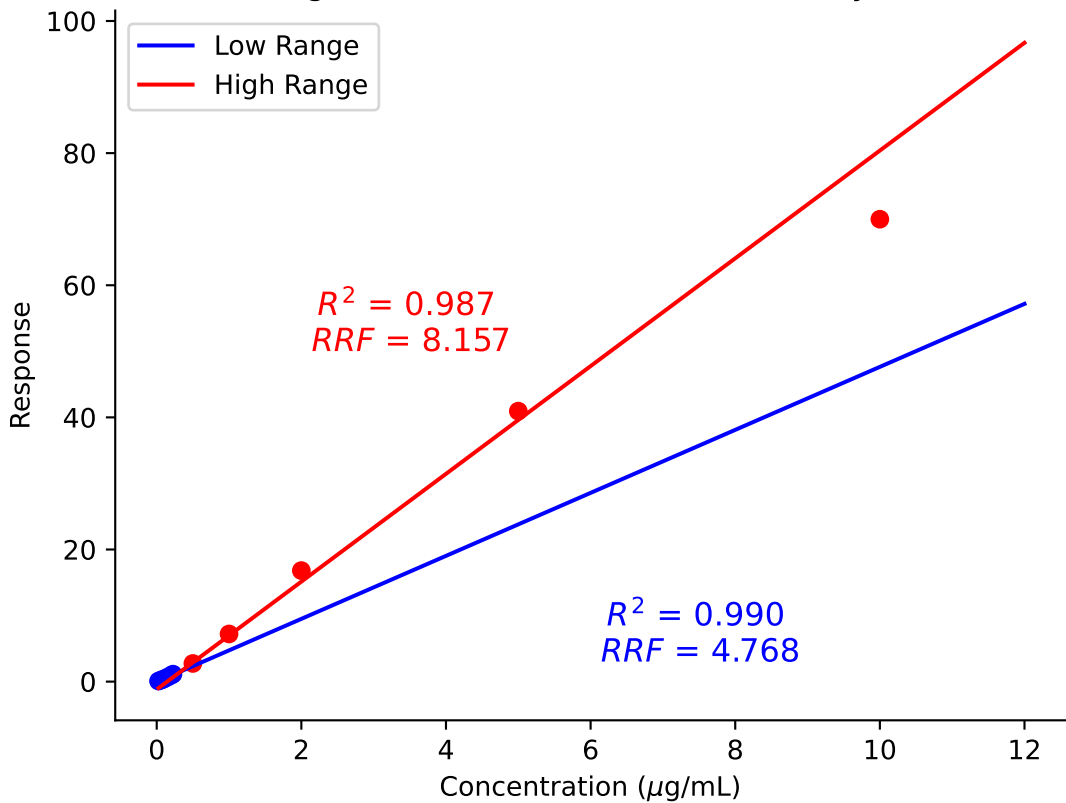

Supplement: Supplementary file 5 [file ac5c04247_si_005.zip › Chrysene-SVOC-EWandHigh.pdf]

# Change in RRF with Concentration (Chrysene)

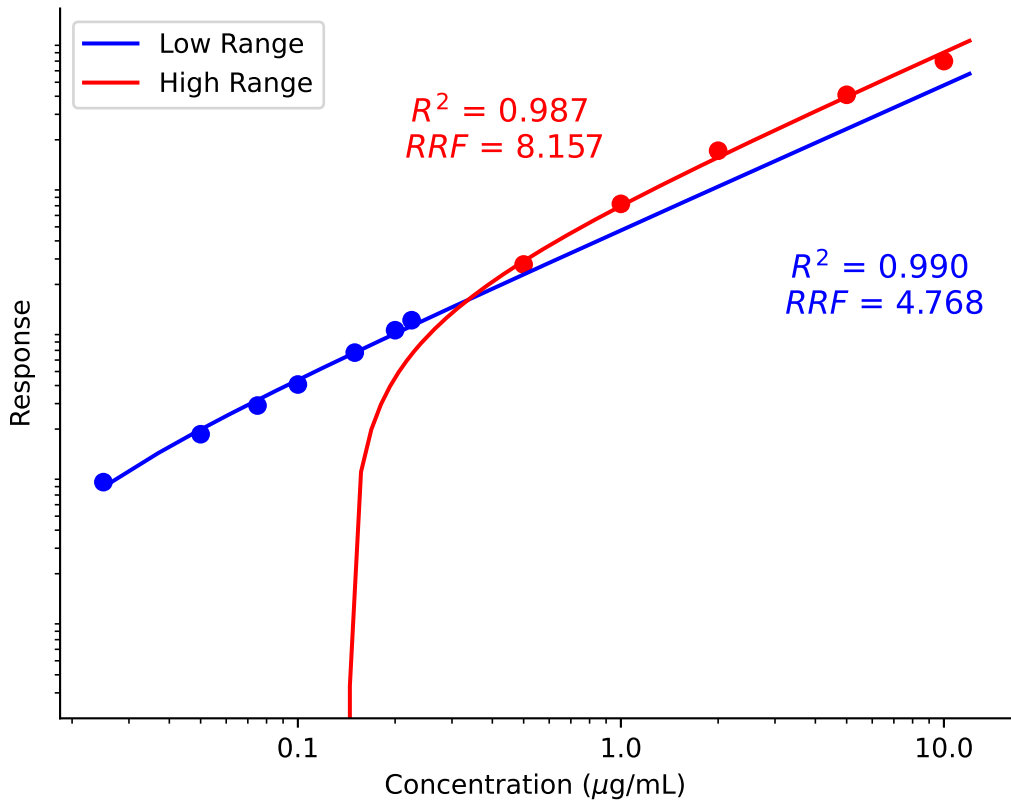

Supplement: Supplementary file 5 [file ac5c04247_si_005.zip › Chrysene-SVOCloglog-EWandHigh.pdf]

Change in RRF with Concentration (Chrysene)

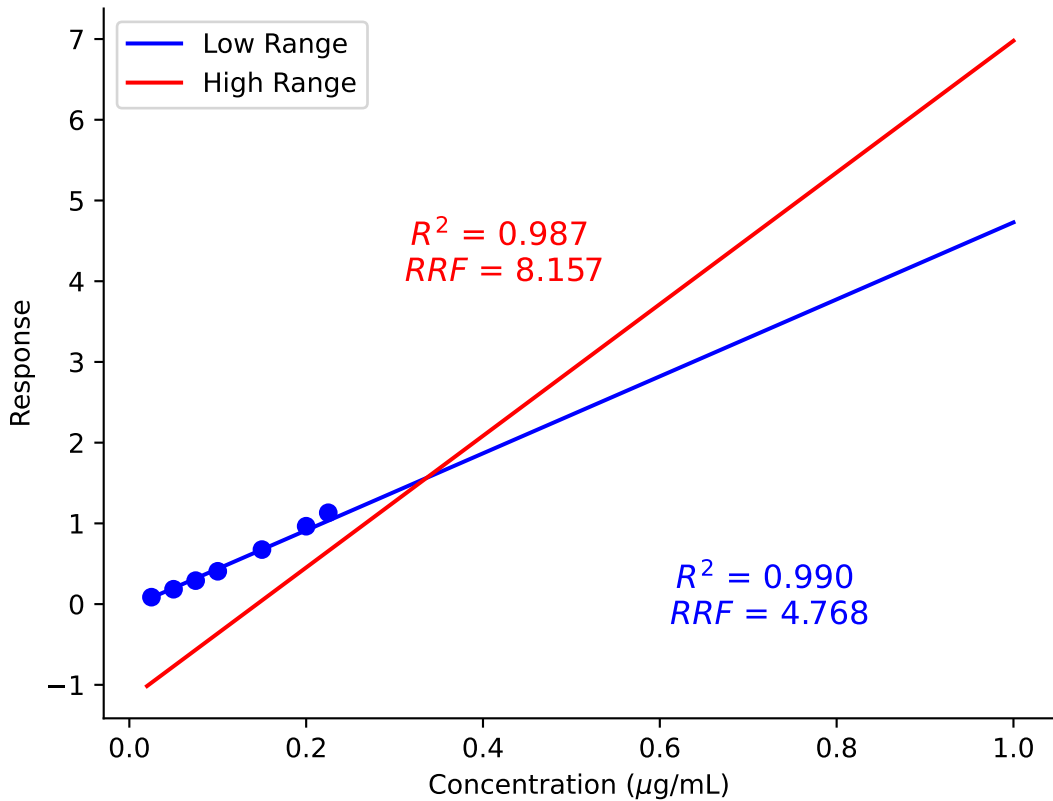

Supplement: Supplementary file 5 [file ac5c04247_si_005.zip › Chrysene-SVOC-LowView.pdf]

Change in RRF with Concentration (Decamethylcyclopentasiloxane D5 (<sup>13</sup>C-10))

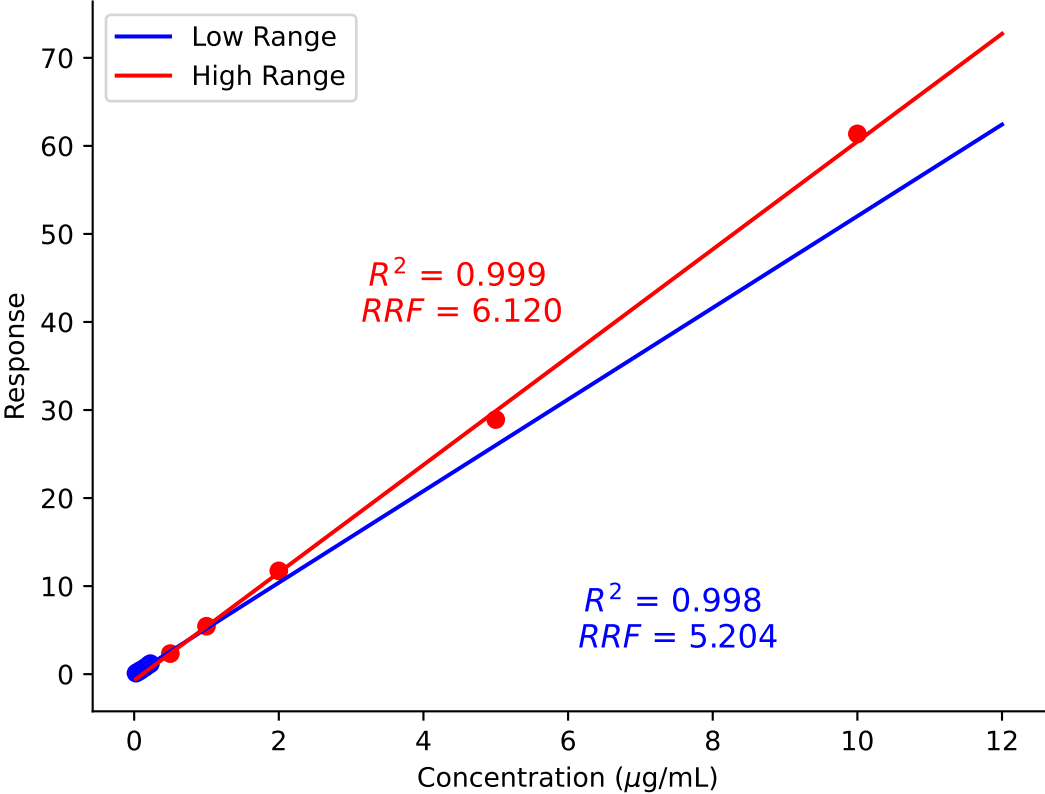

Supplement: Supplementary file 5 [file ac5c04247_si_005.zip › Decamethylcyclopentasiloxane D5 (13C-10)-SVOC-EWandHigh.pdf]

Change in RRF with Concentration (Decamethylcyclopentasiloxane D5 (<sup>13</sup>C-10))

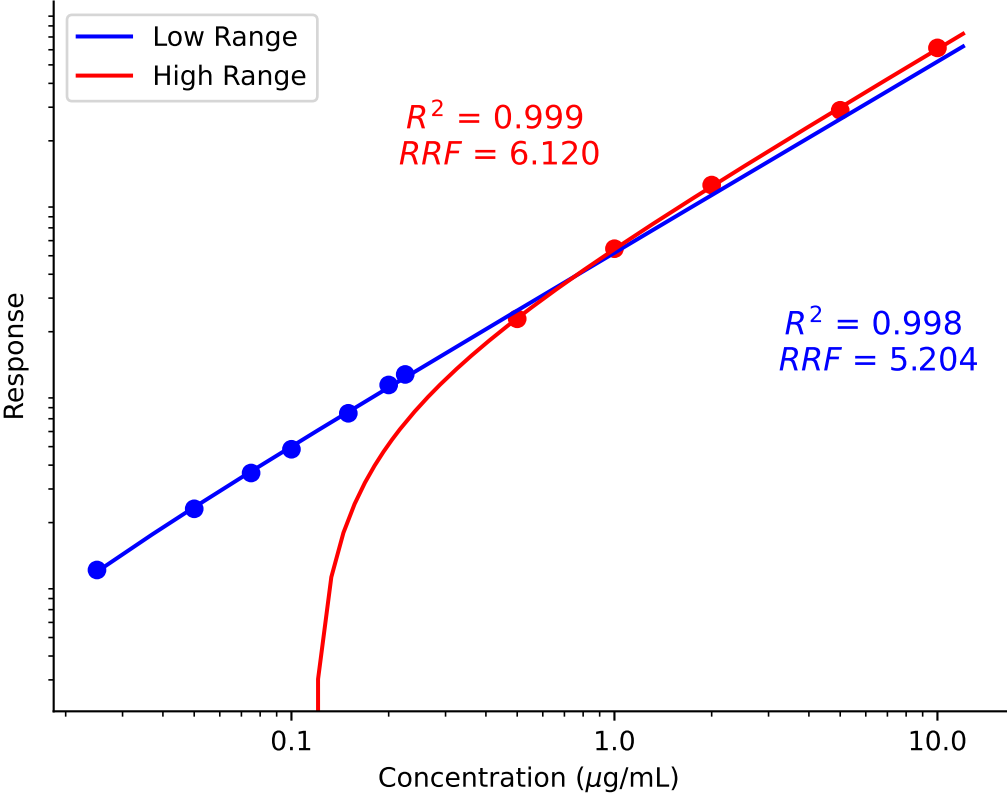

Supplement: Supplementary file 5 [file ac5c04247_si_005.zip › Decamethylcyclopentasiloxane D5 (13C-10)-SVOCloglog-EWandHigh.pdf]

Change in RRF with Concentration (Dibutyl phthalate)

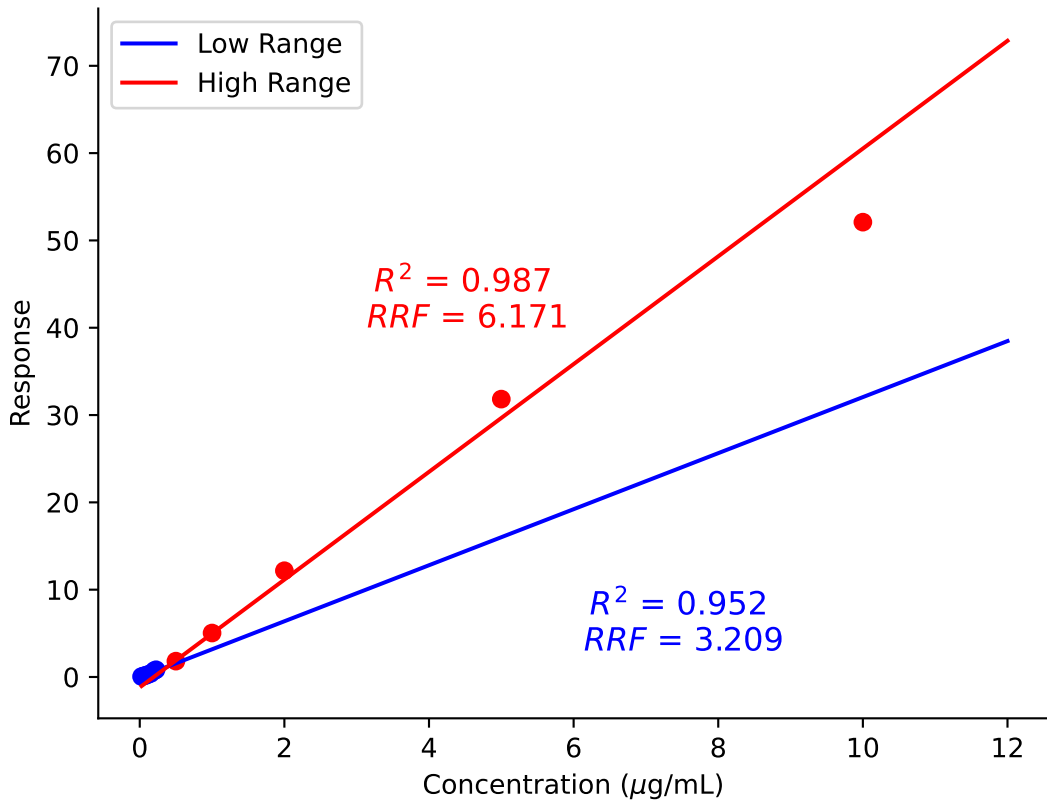

Supplement: Supplementary file 5 [file ac5c04247_si_005.zip › Dibutyl phthalate-SVOC-EWandHigh.pdf]

# Change in RRF with Concentration (Dibutyl phthalate)

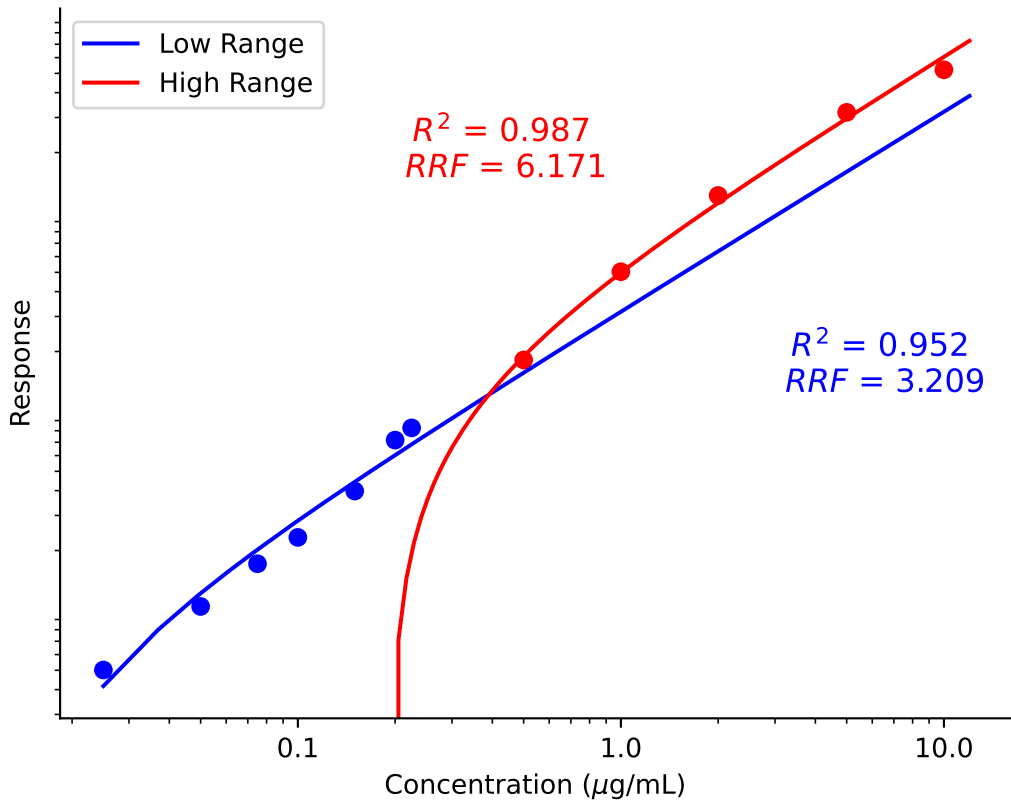

Supplement: Supplementary file 5 [file ac5c04247_si_005.zip › Dibutyl phthalate-SVOCloglog-EWandHigh.pdf]

Change in RRF with Concentration (Dibutyl phthalate)

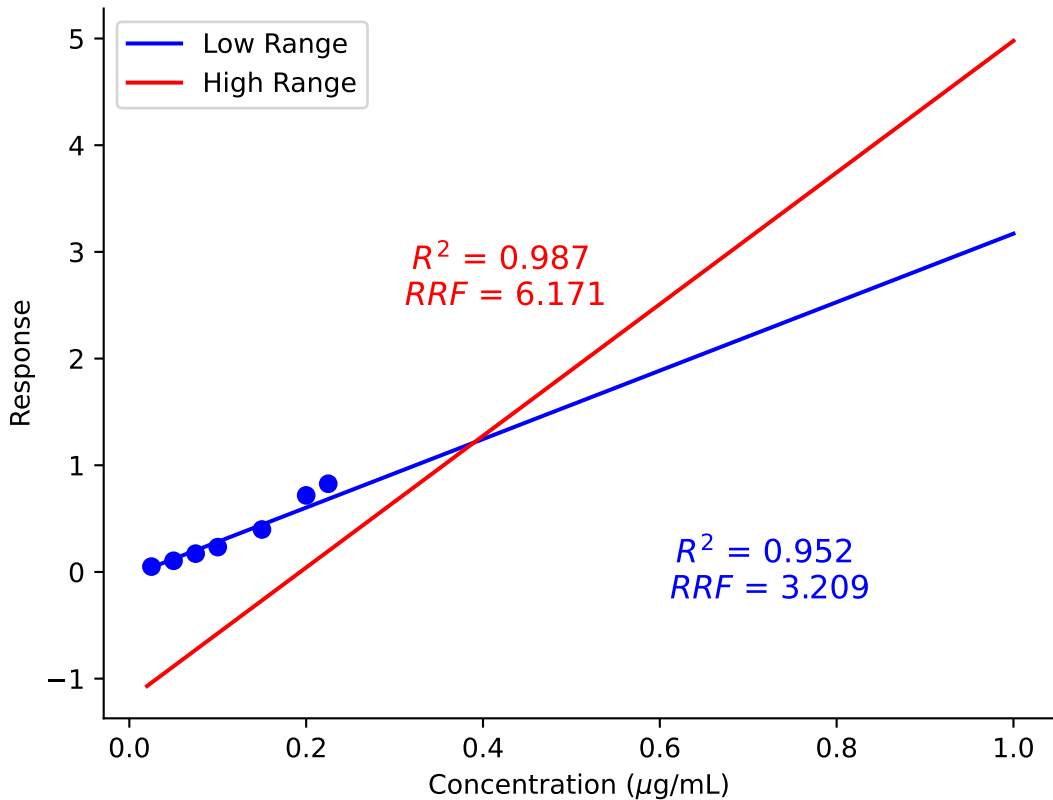

Supplement: Supplementary file 5 [file ac5c04247_si_005.zip › Dibutyl phthalate-SVOC-LowView.pdf]

Change in RRF with Concentration (Diethyl Phthalate)

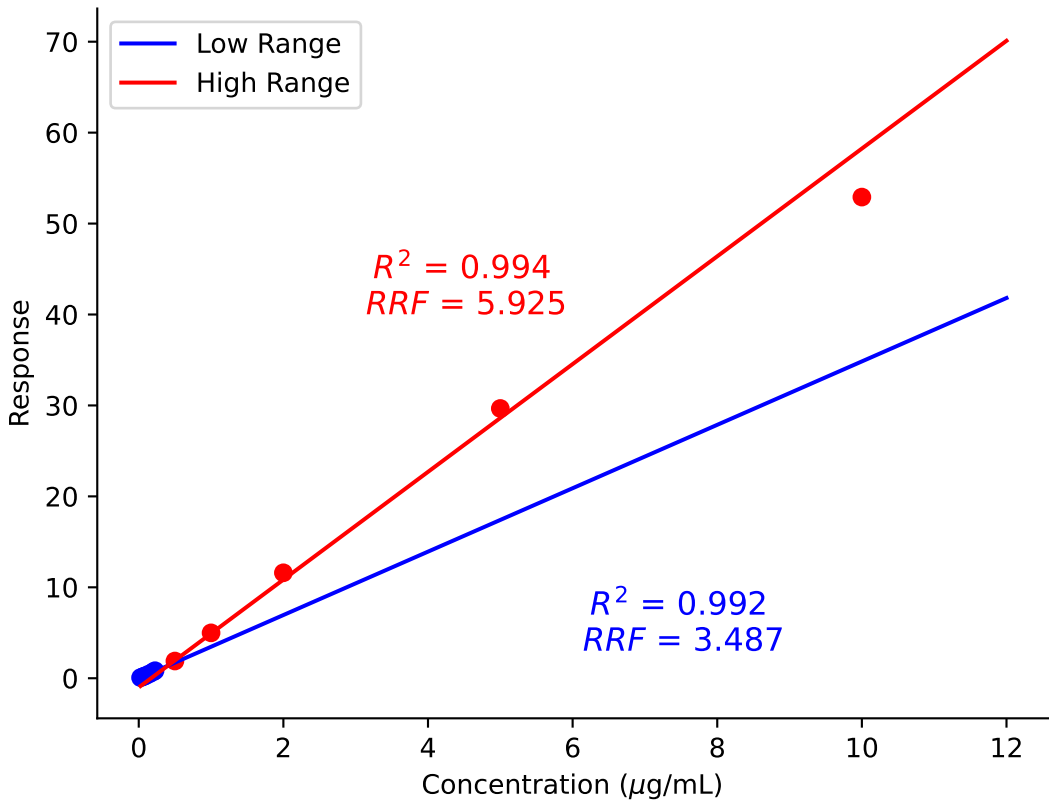

Supplement: Supplementary file 5 [file ac5c04247_si_005.zip › Diethyl Phthalate-SVOC-EWandHigh.pdf]

# Change in RRF with Concentration (Diethyl Phthalate)

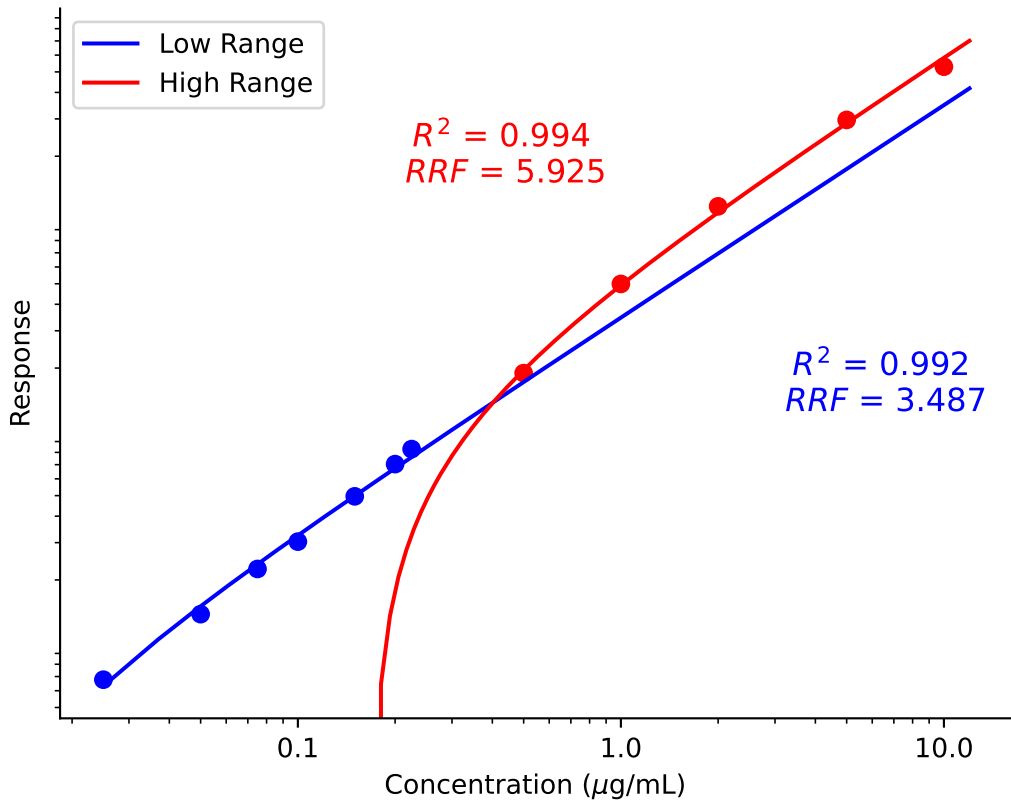

Supplement: Supplementary file 5 [file ac5c04247_si_005.zip › Diethyl Phthalate-SVOCloglog-EWandHigh.pdf]

# Change in RRF with Concentration (Diethyl Phthalate)

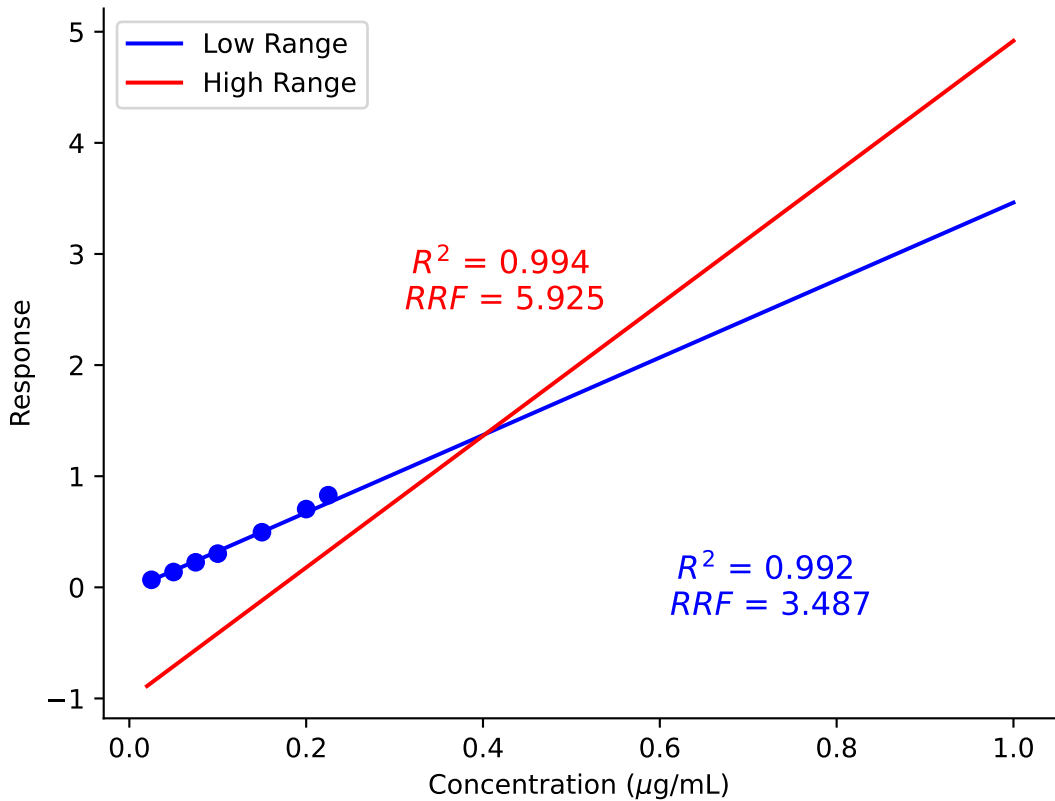

Supplement: Supplementary file 5 [file ac5c04247_si_005.zip › Diethyl Phthalate-SVOC-LowView.pdf]

# Change in RRF with Concentration (Dipentyl phthalate)

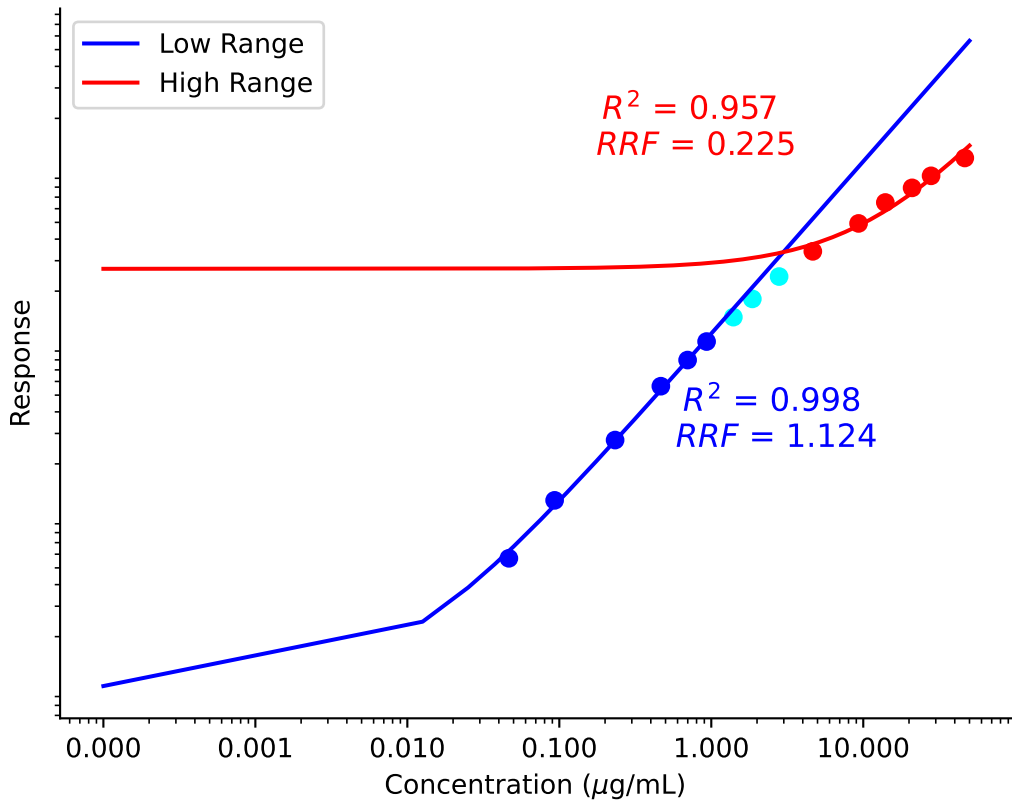

Supplement: Supplementary file 5 [file ac5c04247_si_005.zip › Dipentyl phthalate-NVOCloglog-EWandHigh.pdf]

Change in RRF with Concentration (Diphenylamine)

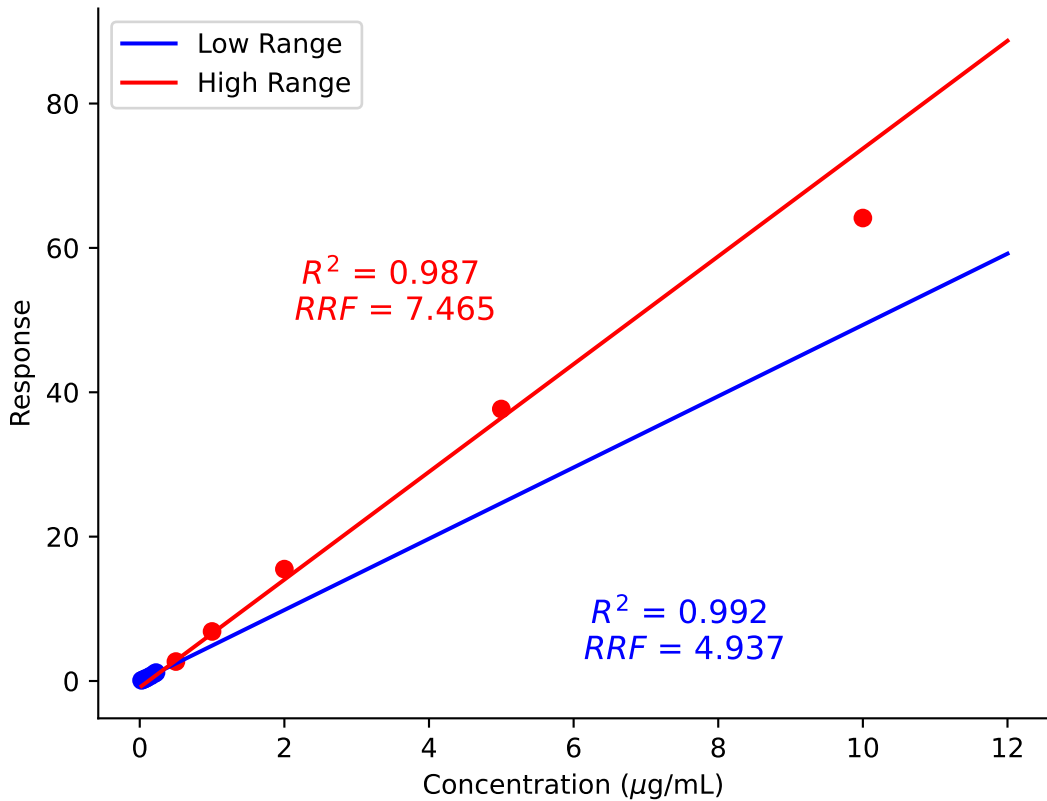

Supplement: Supplementary file 5 [file ac5c04247_si_005.zip › Diphenylamine-SVOC-EWandHigh.pdf]

# Change in RRF with Concentration (Diphenylamine)

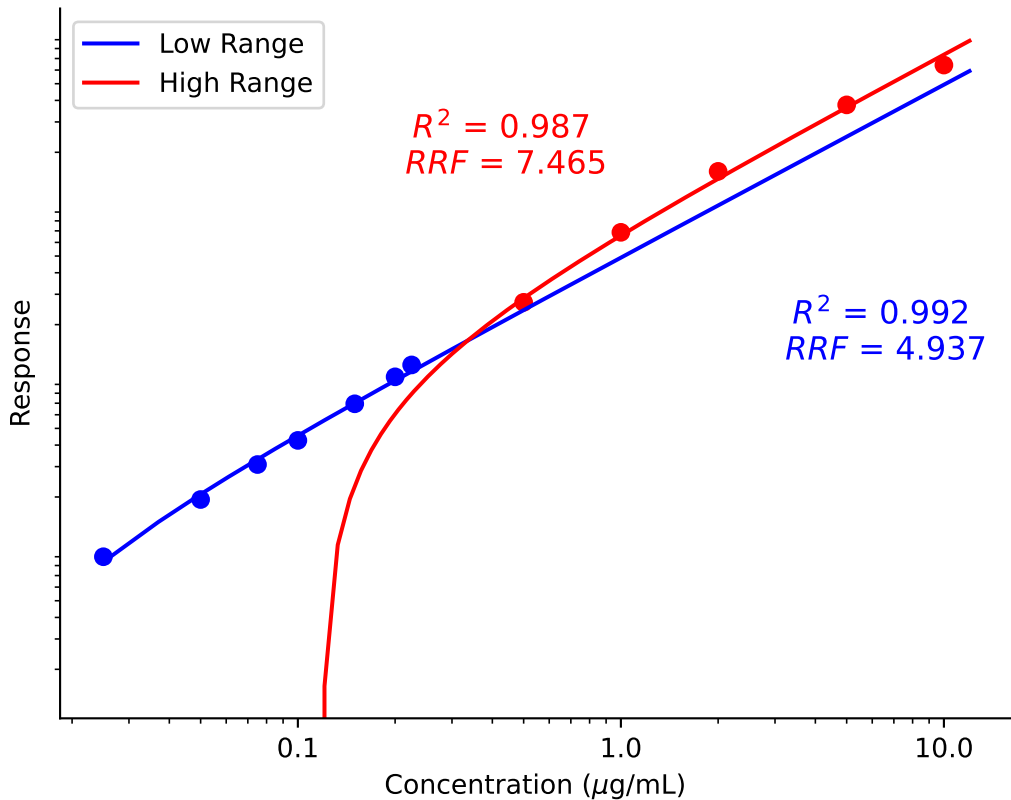

Supplement: Supplementary file 5 [file ac5c04247_si_005.zip › Diphenylamine-SVOCloglog-EWandHigh.pdf]

Change in RRF with Concentration (Diphenylamine)

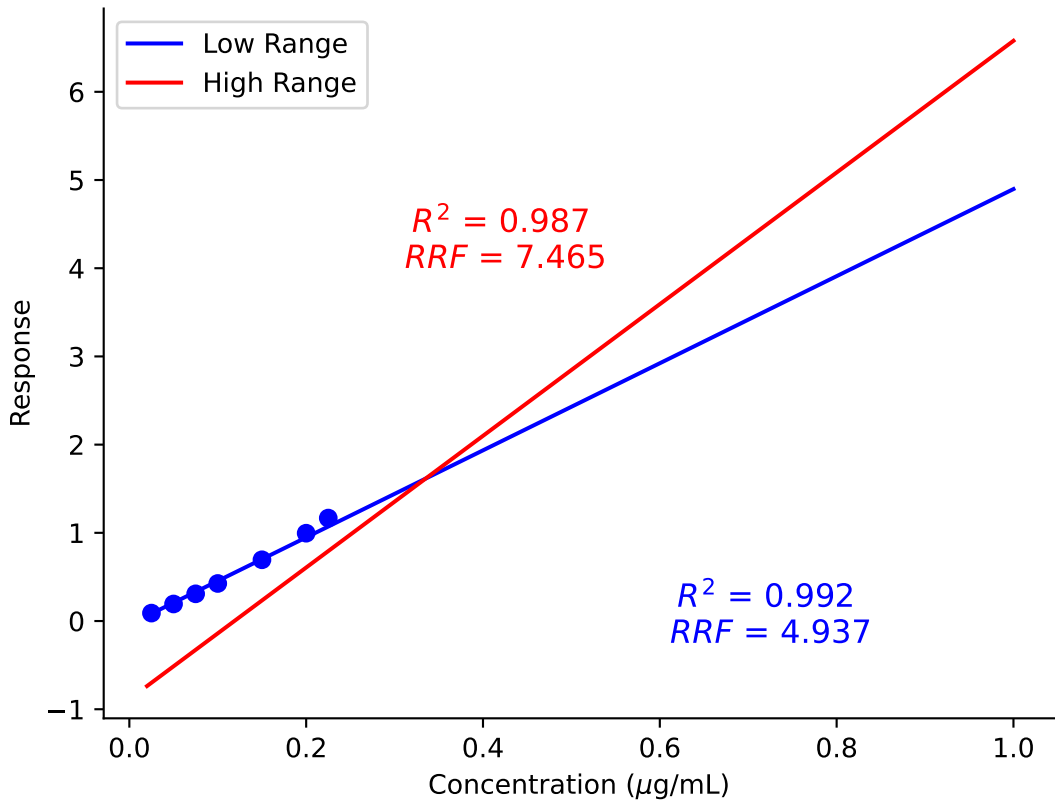

Supplement: Supplementary file 5 [file ac5c04247_si_005.zip › Diphenylamine-SVOC-LowView.pdf]

# Change in RRF with Concentration (Dodecanoic acid)

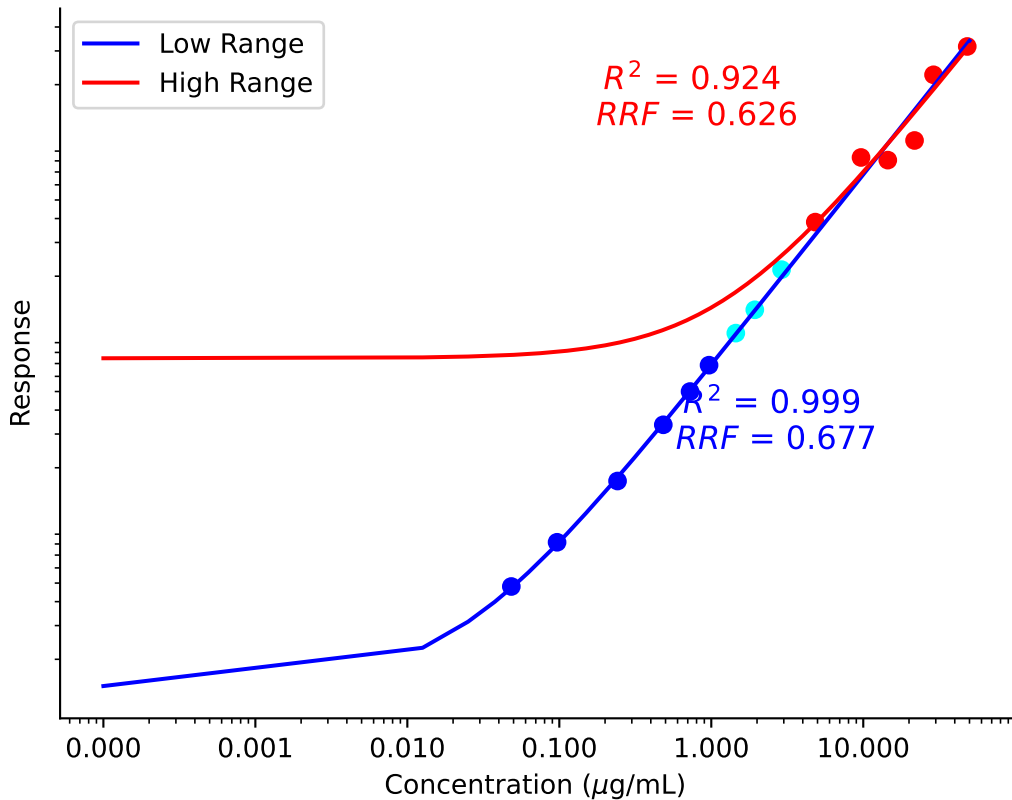

Supplement: Supplementary file 5 [file ac5c04247_si_005.zip › Dodecanoic acid-NVOCloglog-EWandHigh.pdf]

Change in RRF with Concentration (Eicosane)

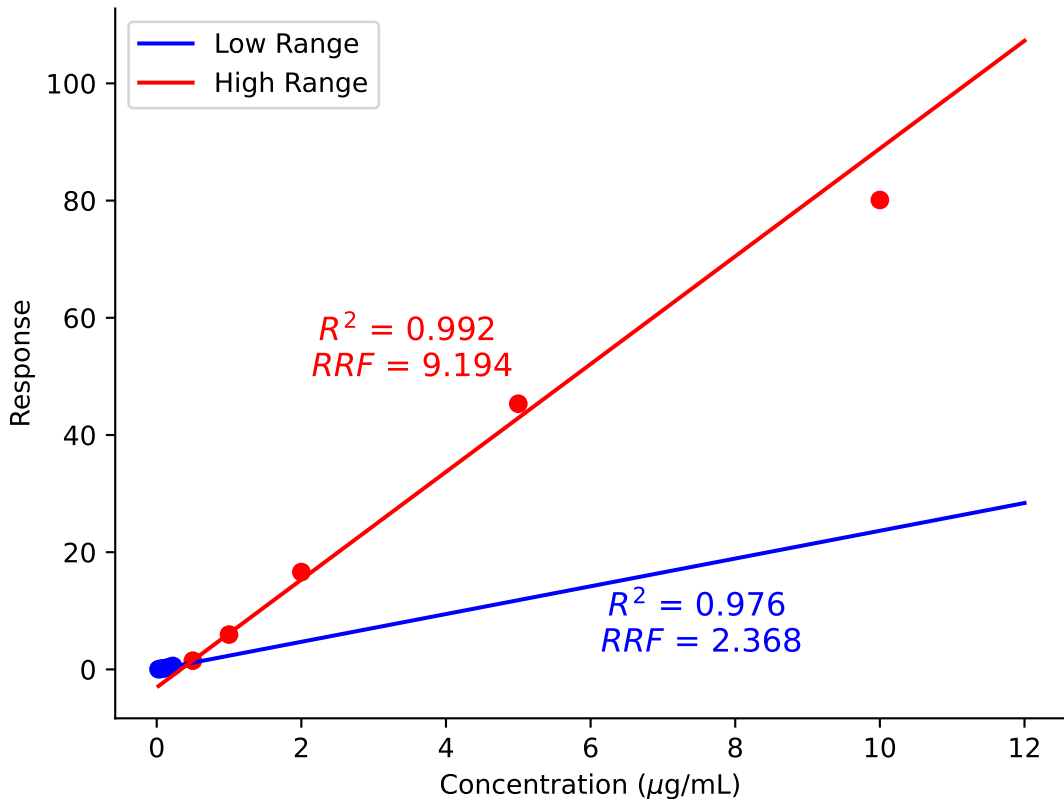

Supplement: Supplementary file 5 [file ac5c04247_si_005.zip › Eicosane-SVOC-EWandHigh.pdf]

# Change in RRF with Concentration (Eicosane)

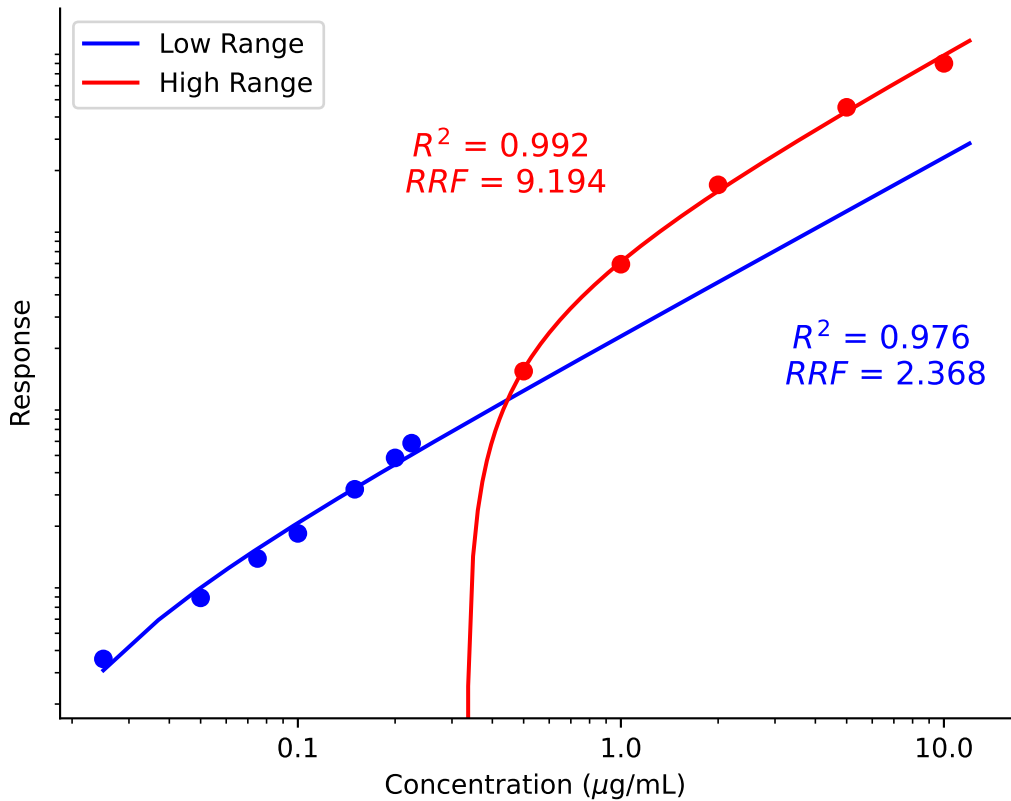

Supplement: Supplementary file 5 [file ac5c04247_si_005.zip › Eicosane-SVOCloglog-EWandHigh.pdf]

Change in RRF with Concentration (Eicosane)

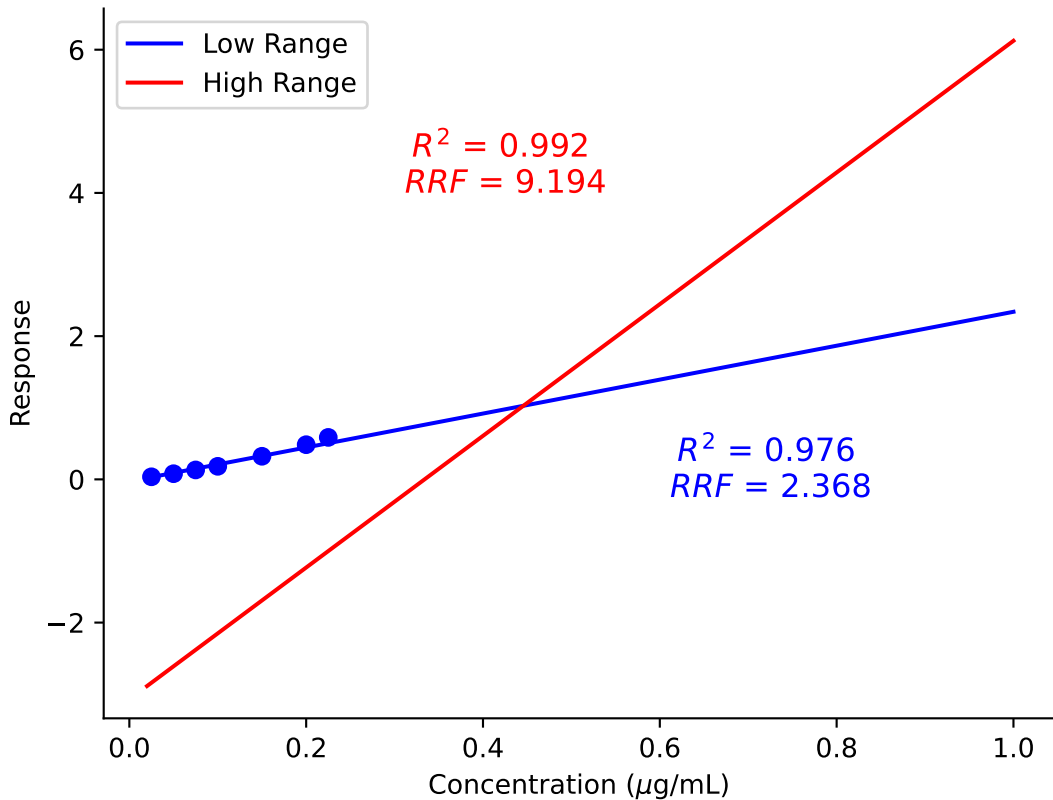

Supplement: Supplementary file 5 [file ac5c04247_si_005.zip › Eicosane-SVOC-LowView.pdf]

Change in RRF with Concentration (Hexadecamethylcyclooctasiloxane (D8))

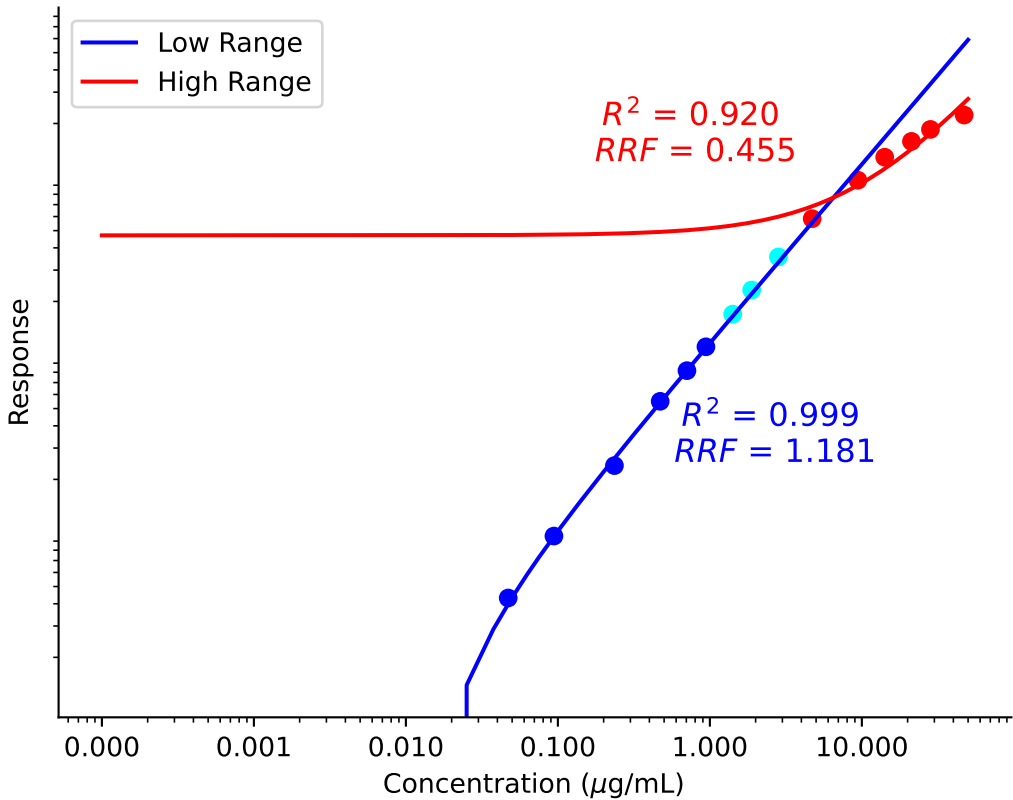

Supplement: Supplementary file 5 [file ac5c04247_si_005.zip › Hexadecamethylcyclooctasiloxane (D8)-NVOCloglog-EWandHigh.pdf]

# Change in RRF with Concentration (Hexadecanoic acid, methyl ester)

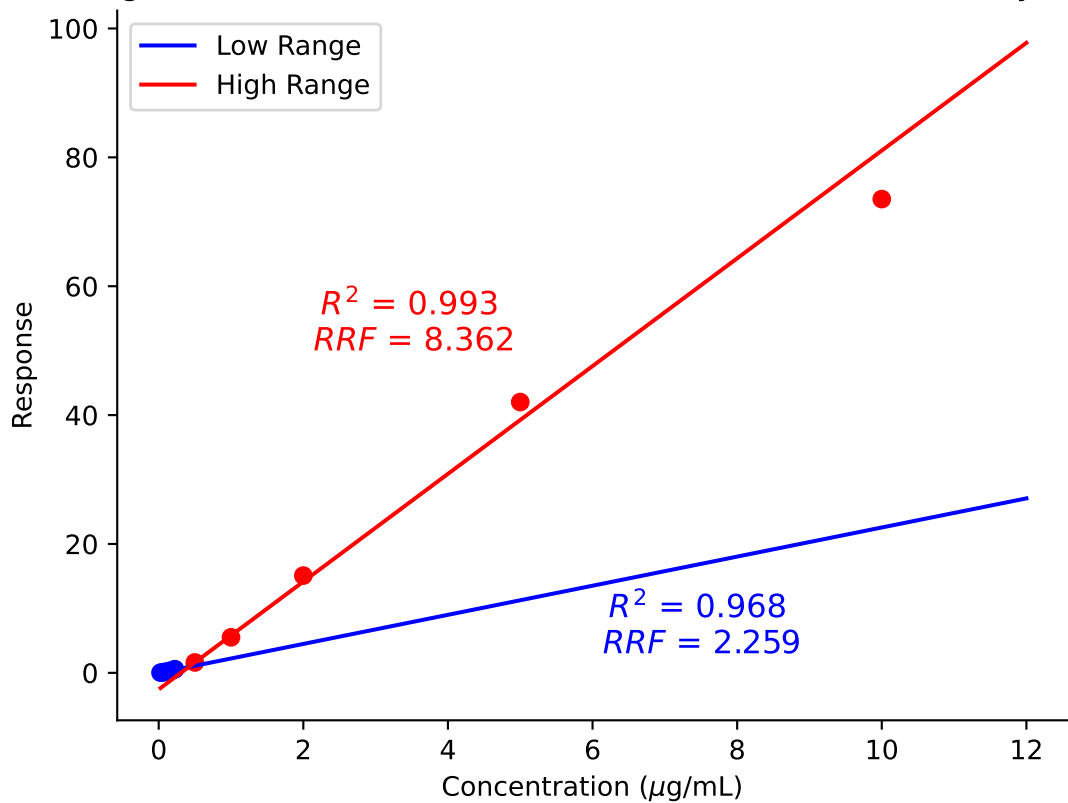

Supplement: Supplementary file 5 [file ac5c04247_si_005.zip › Hexadecanoic acid, methyl ester-SVOC-EWandHigh.pdf]

# Change in RRF with Concentration (Hexadecanoic acid, methyl ester)

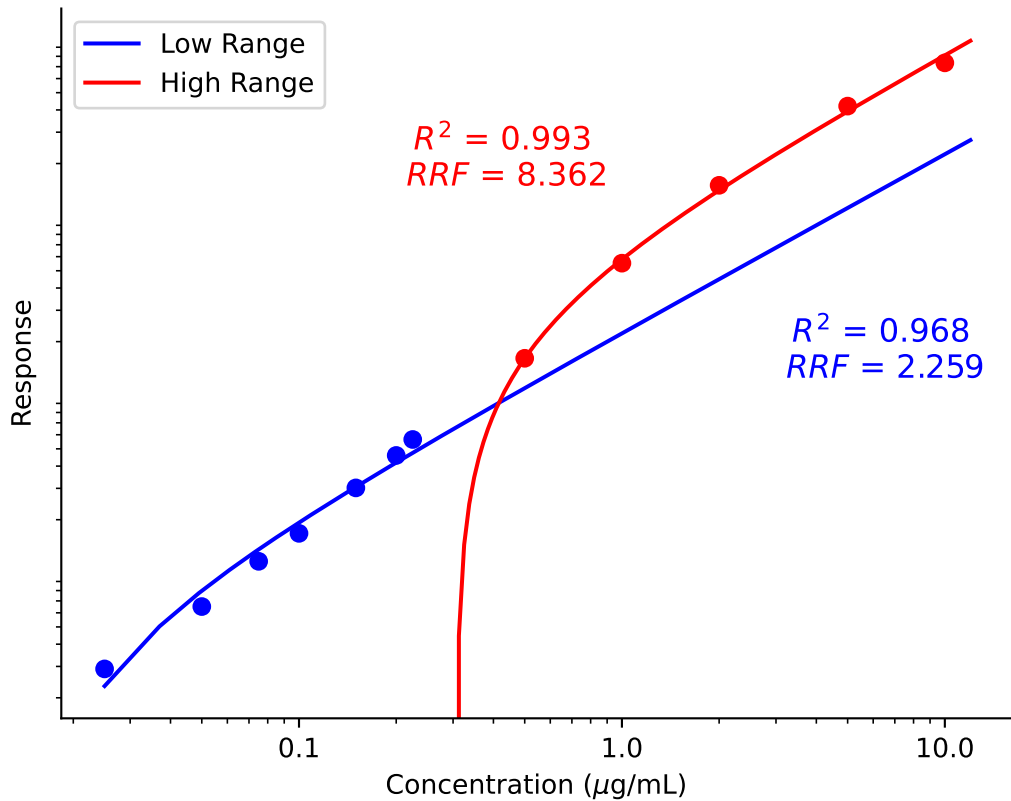

Supplement: Supplementary file 5 [file ac5c04247_si_005.zip › Hexadecanoic acid, methyl ester-SVOCloglog-EWandHigh.pdf]

Change in RRF with Concentration (Hexadecanoic acid, methyl ester)

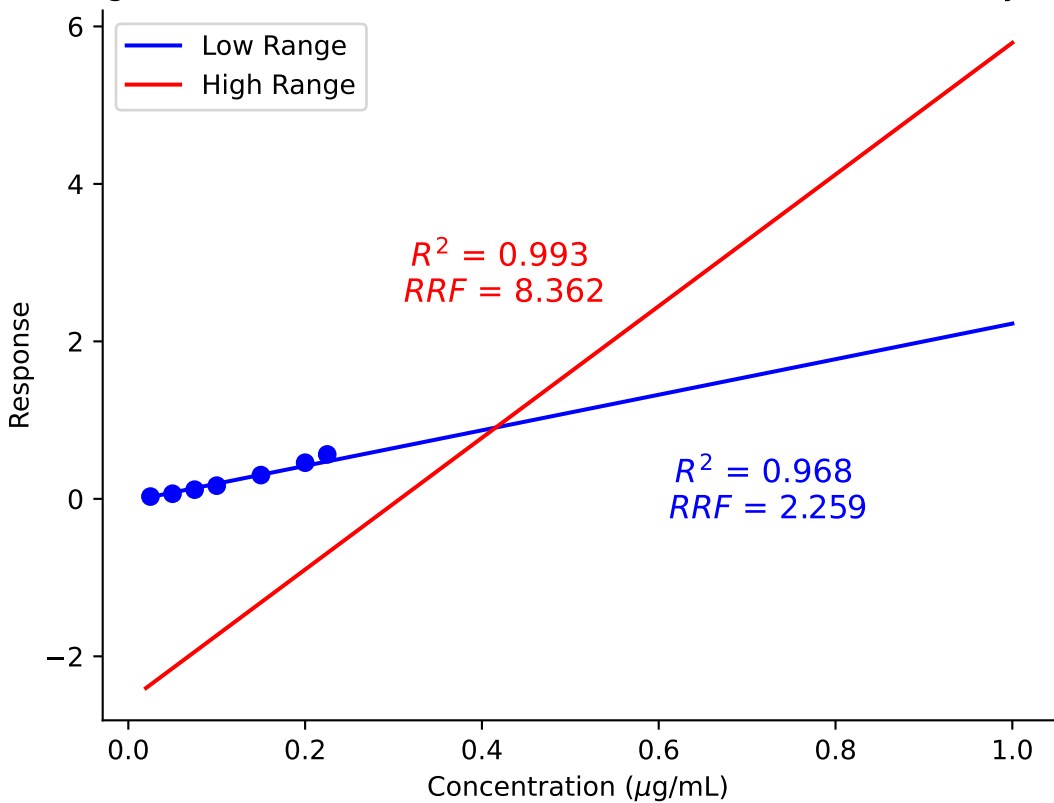

Supplement: Supplementary file 5 [file ac5c04247_si_005.zip › Hexadecanoic acid, methyl ester-SVOC-LowView.pdf]

Change in RRF with Concentration (Hexanedioic acid, bis(2-ethylhexyl) ester)

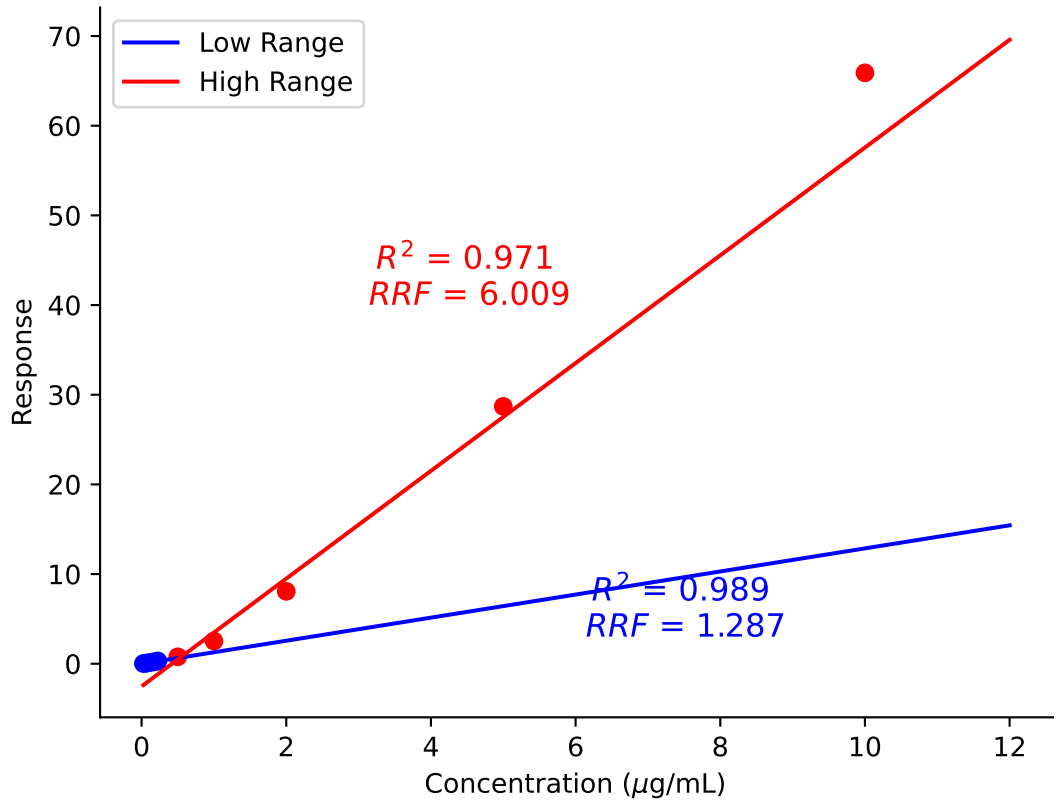

Supplement: Supplementary file 5 [file ac5c04247_si_005.zip › Hexanedioic acid, bis(2-ethylhexyl) ester-SVOC-EWandHigh.pdf]

Change in RRF with Concentration (Hexanedioic acid, bis(2-ethylhexyl) ester)

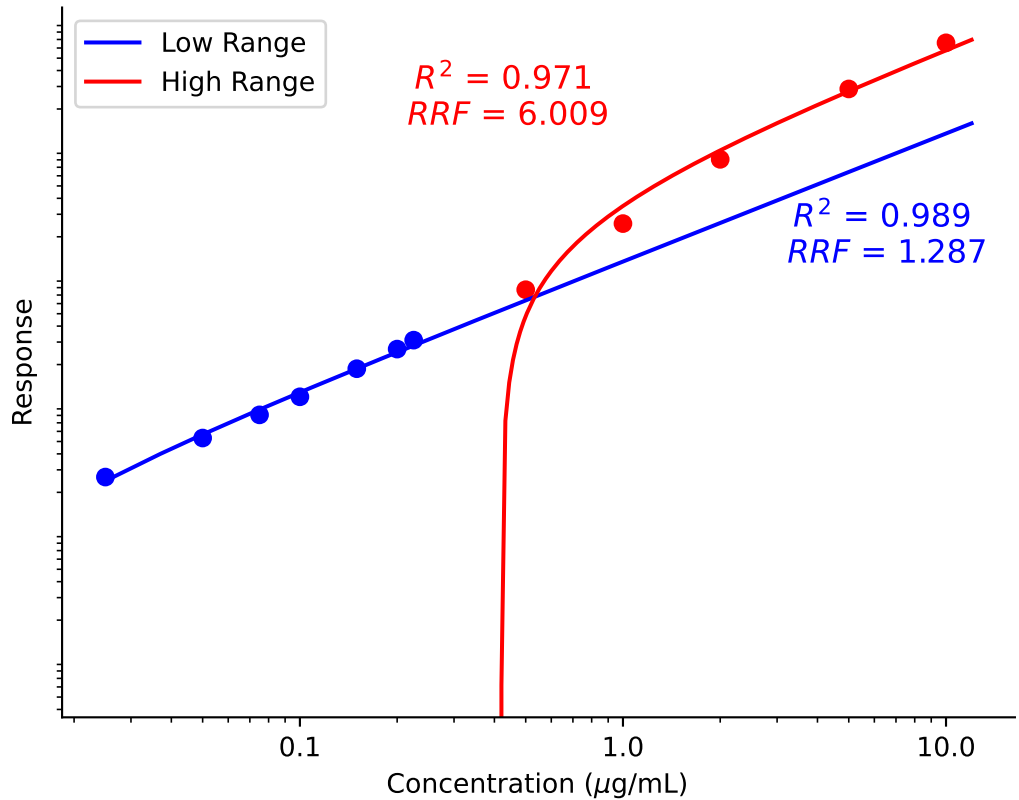

Supplement: Supplementary file 5 [file ac5c04247_si_005.zip › Hexanedioic acid, bis(2-ethylhexyl) ester-SVOCloglog-EWandHigh.pdf]

Change in RRF with Concentration (Hexanedioic acid, bis(2-ethylhexyl) ester)

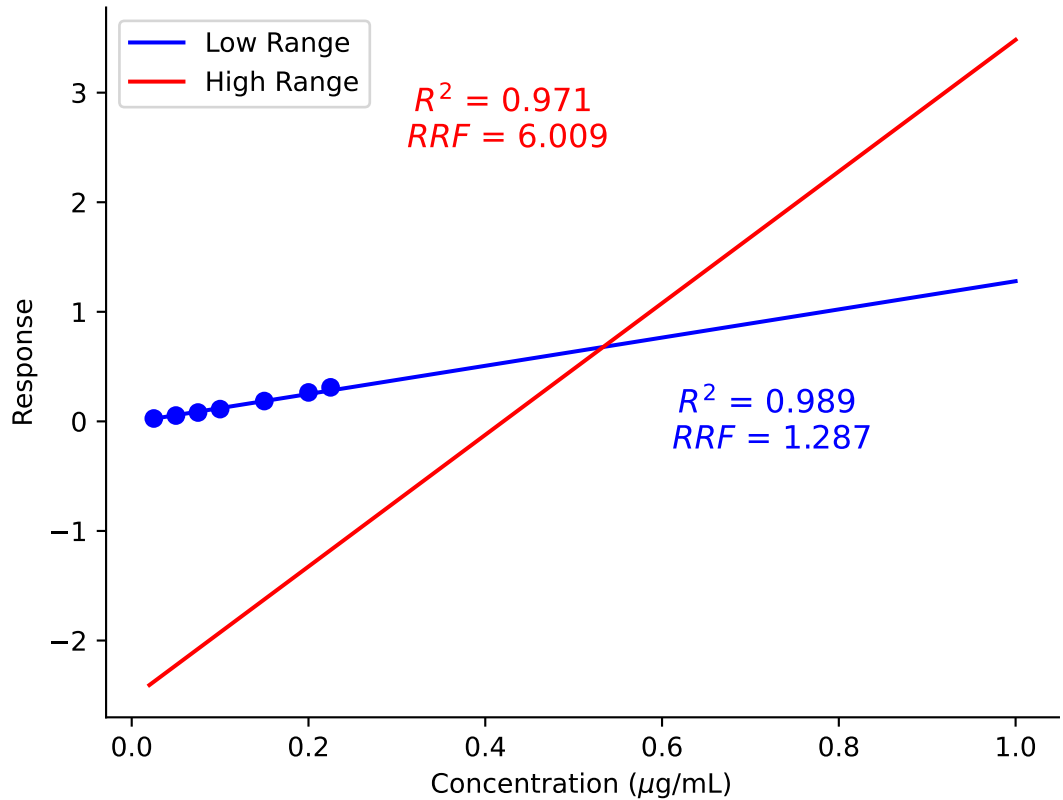

Supplement: Supplementary file 5 [file ac5c04247_si_005.zip › Hexanedioic acid, bis(2-ethylhexyl) ester-SVOC-LowView.pdf]

Change in RRF with Concentration (Indeno[1,2,3-cd]pyrene)

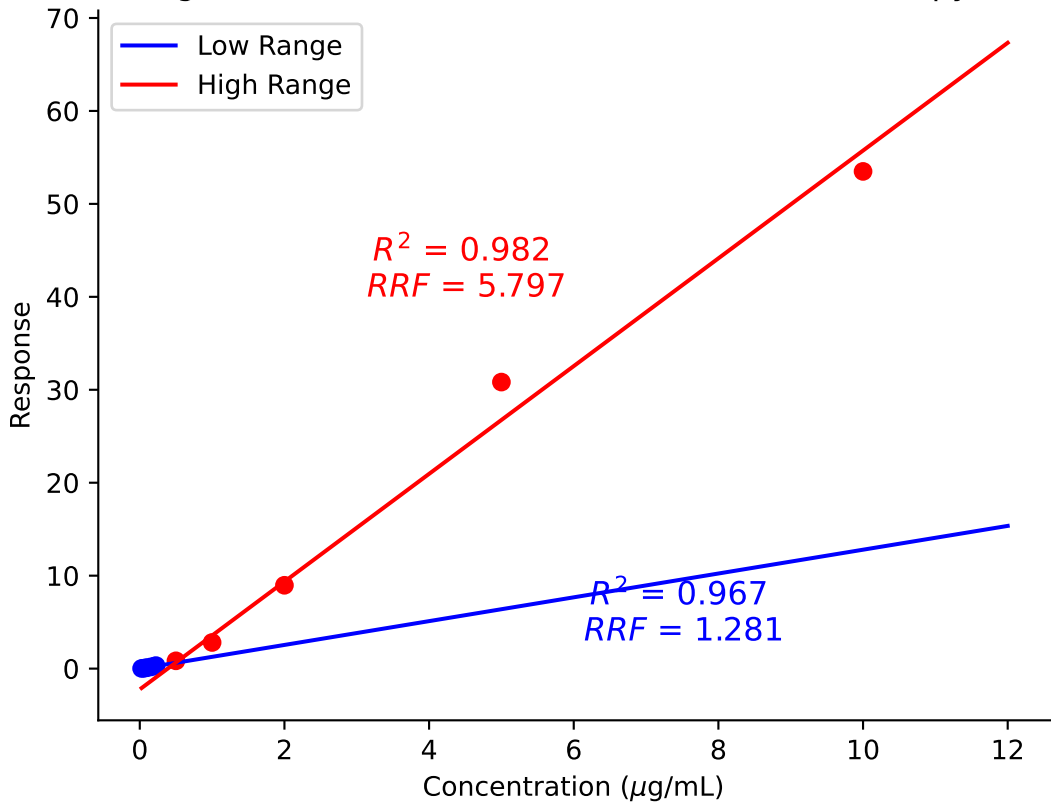

Supplement: Supplementary file 5 [file ac5c04247_si_005.zip › Indeno[1,2,3-cd]pyrene-SVOC-EWandHigh.pdf]

# Change in RRF with Concentration (Indeno[1,2,3-cd]pyrene)

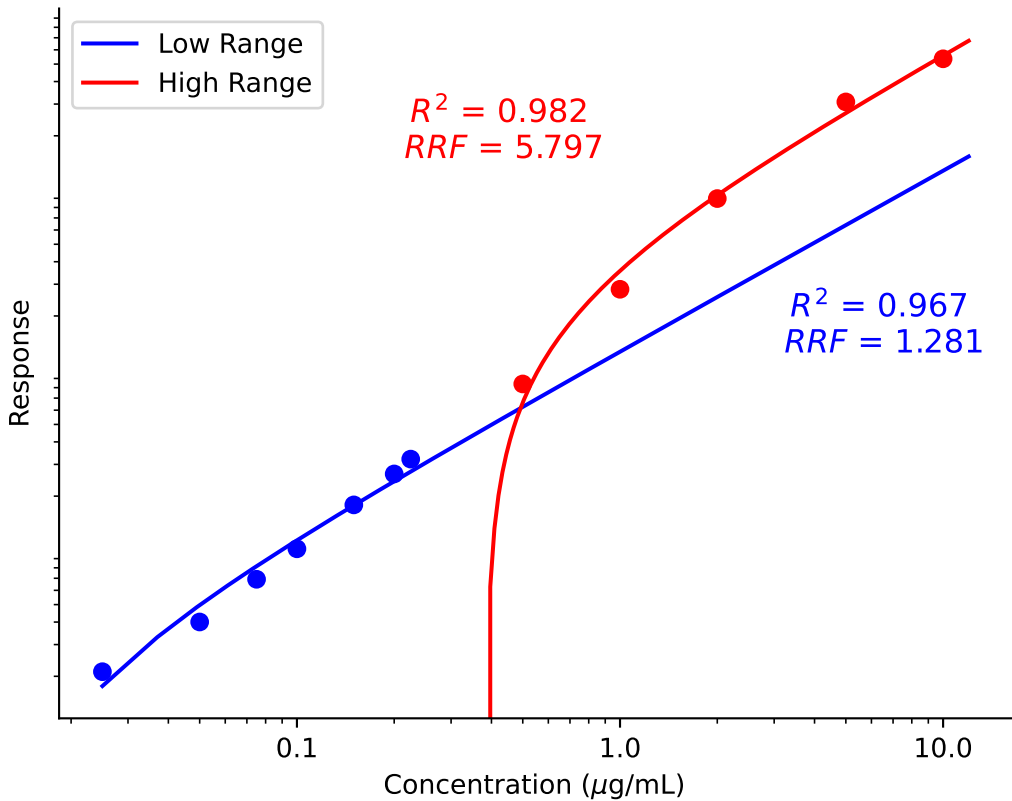

Supplement: Supplementary file 5 [file ac5c04247_si_005.zip › Indeno[1,2,3-cd]pyrene-SVOCloglog-EWandHigh.pdf]

Change in RRF with Concentration (Indeno[1,2,3-cd]pyrene)

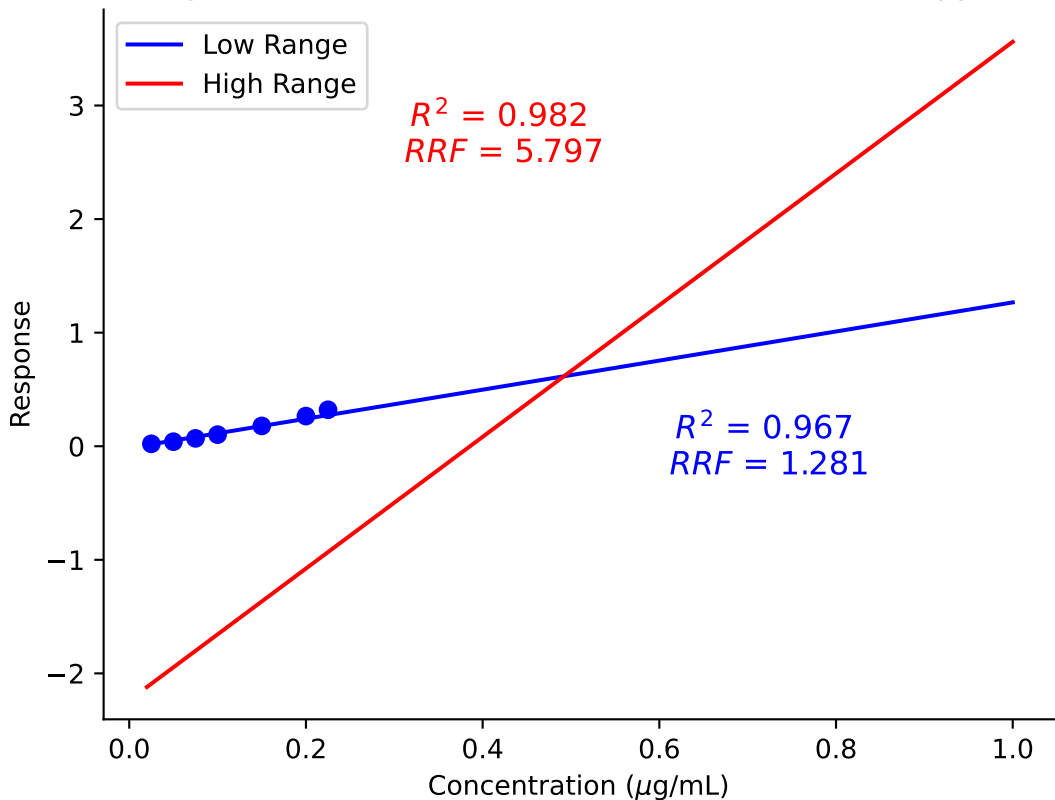

Supplement: Supplementary file 5 [file ac5c04247_si_005.zip › Indeno[1,2,3-cd]pyrene-SVOC-LowView.pdf]

# Change in RRF with Concentration (Irganox 1098)

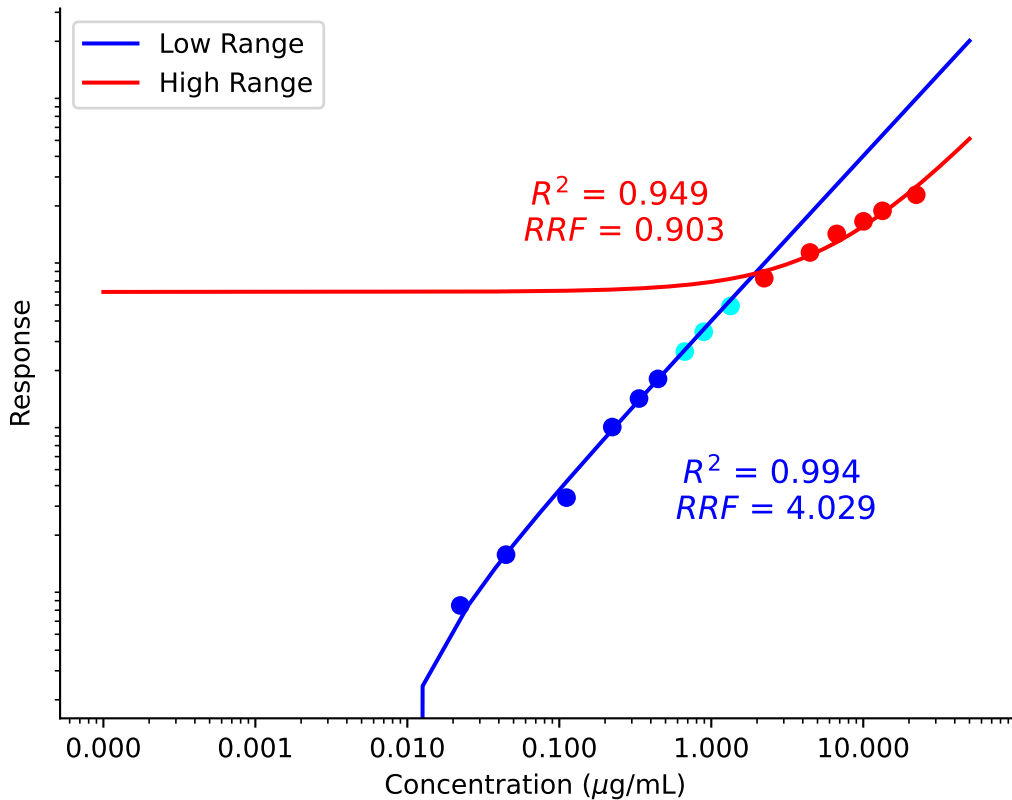

Supplement: Supplementary file 5 [file ac5c04247_si_005.zip › Irganox 1098-NVOCloglog-EWandHigh.pdf]

# Change in RRF with Concentration (Irganox 3114)

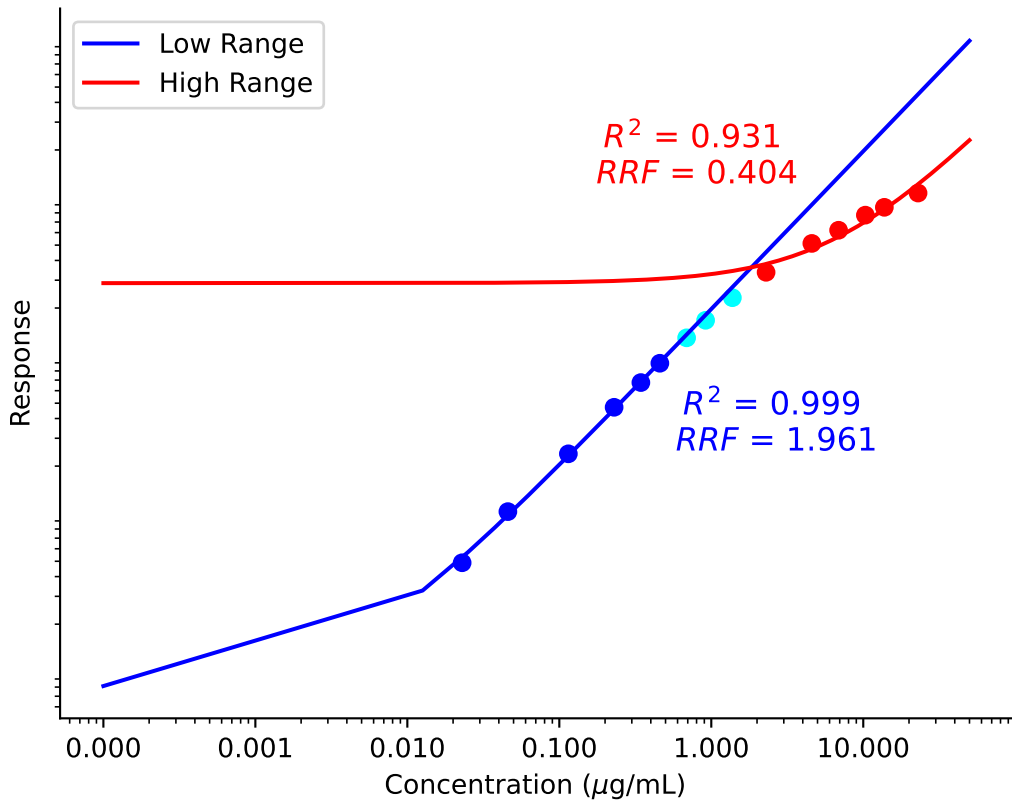

Supplement: Supplementary file 5 [file ac5c04247_si_005.zip › Irganox 3114-NVOCloglog-EWandHigh.pdf]

# Change in RRF with Concentration (Methylparaben)

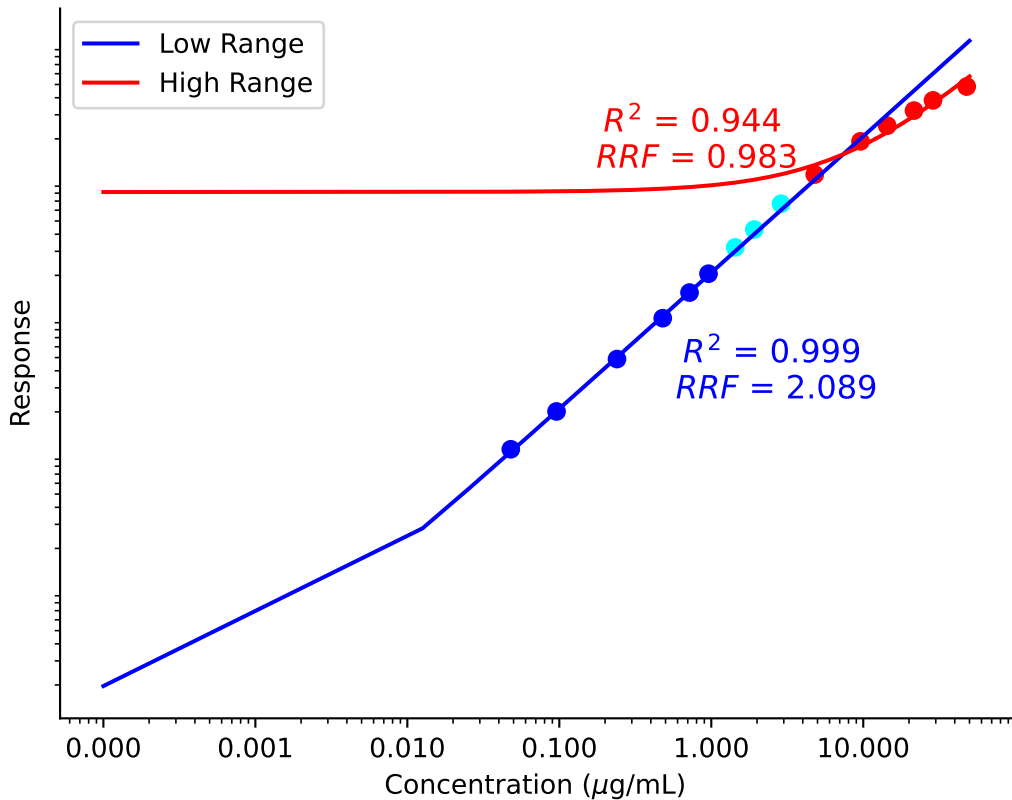

Supplement: Supplementary file 5 [file ac5c04247_si_005.zip › Methylparaben-NVOCloglog-EWandHigh.pdf]

# Change in RRF with Concentration (N-Lauryldiethanolamine)

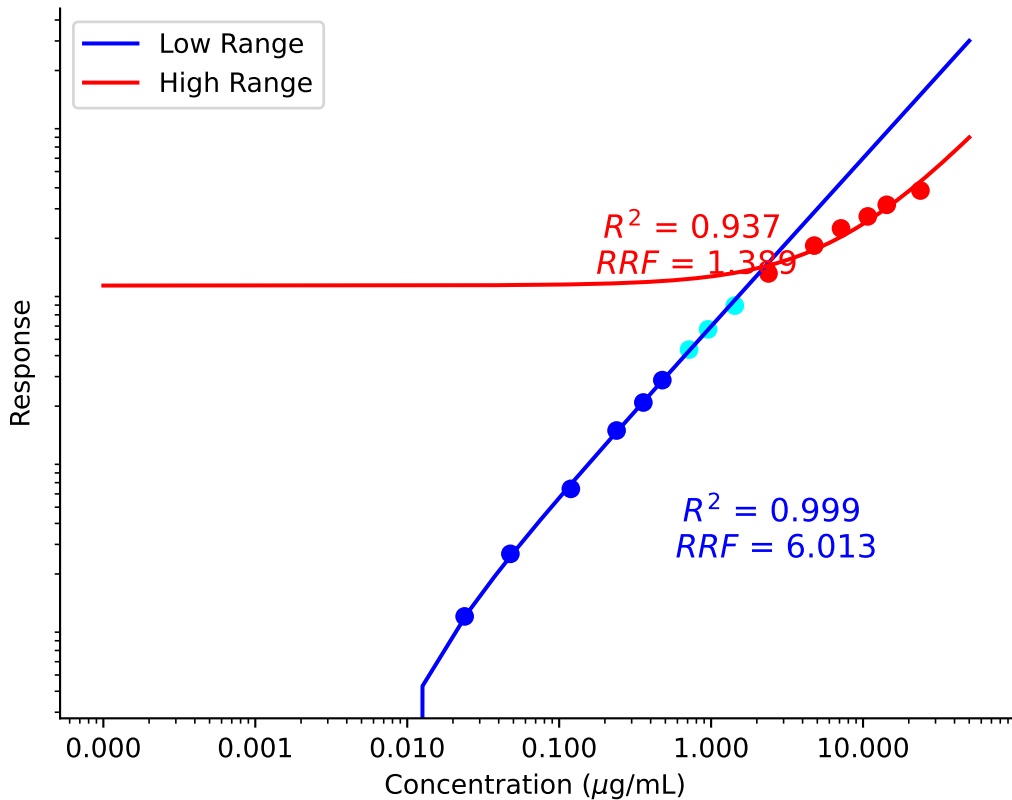

Supplement: Supplementary file 5 [file ac5c04247_si_005.zip › N-Lauryldiethanolamine-NVOCloglog-EWandHigh.pdf]

# Change in RRF with Concentration (Oleamide)

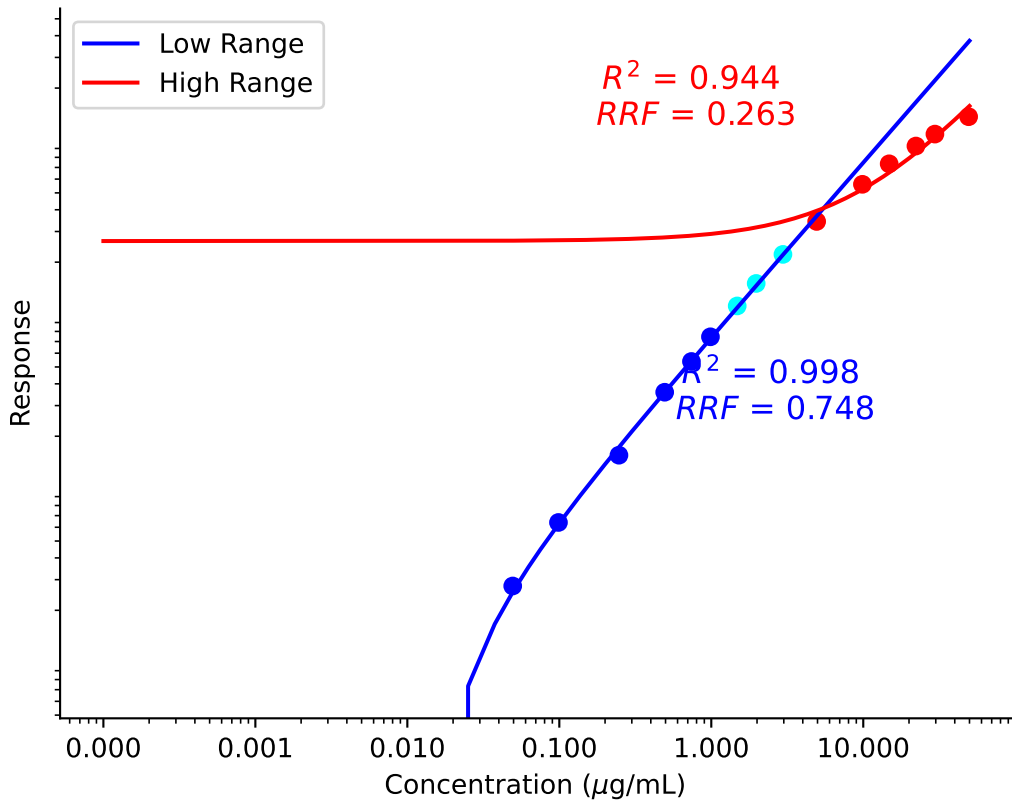

Supplement: Supplementary file 5 [file ac5c04247_si_005.zip › Oleamide-NVOCloglog-EWandHigh.pdf]

# Change in RRF with Concentration (Perfluorooctanoic acid)

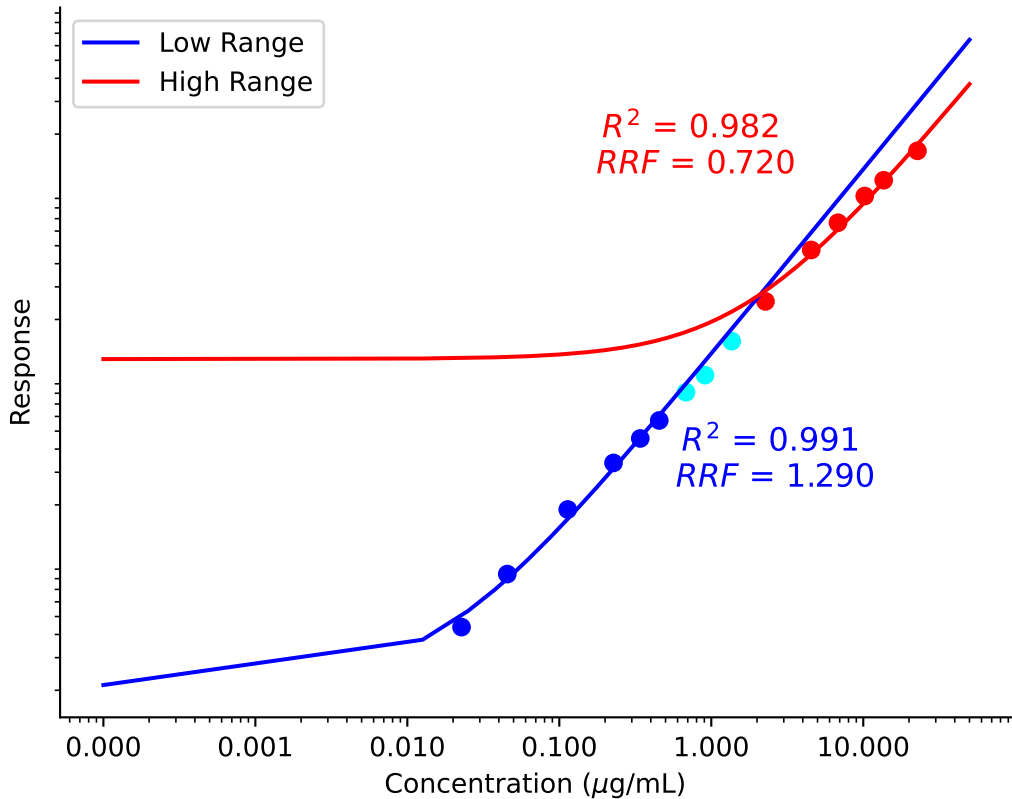

Supplement: Supplementary file 5 [file ac5c04247_si_005.zip › Perfluorooctanoic acid-NVOCloglog-EWandHigh.pdf]

Change in RRF with Concentration (Phenol)

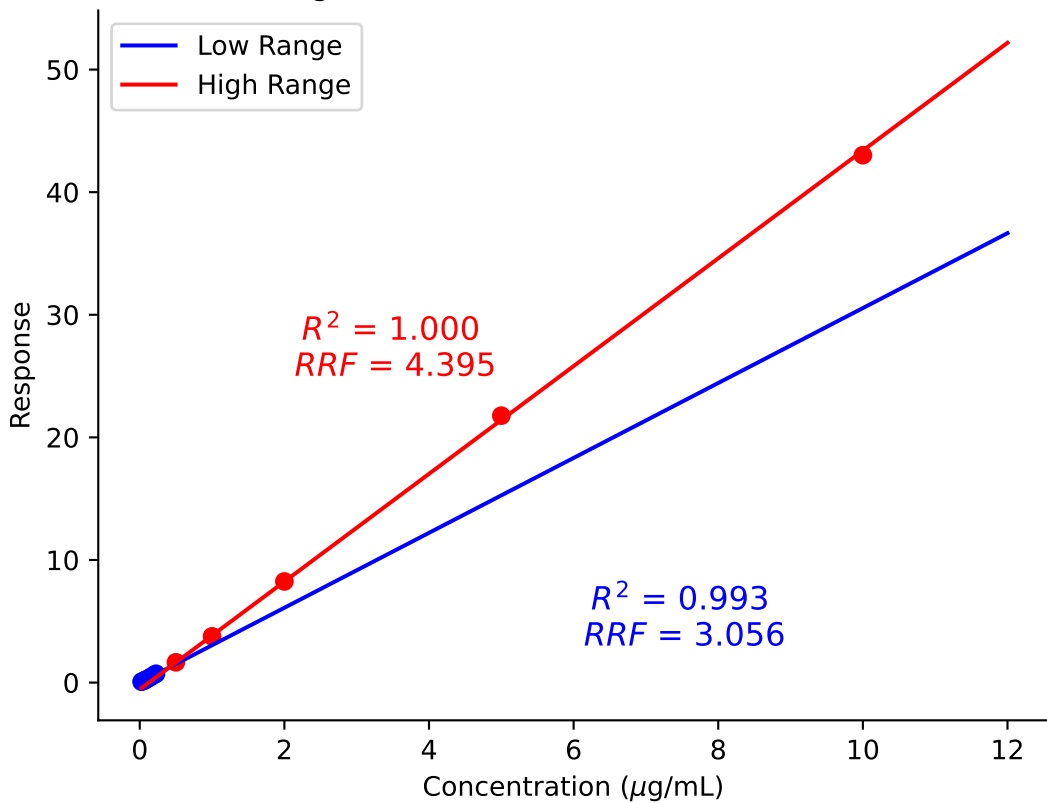

Supplement: Supplementary file 5 [file ac5c04247_si_005.zip › Phenol-SVOC-EWandHigh.pdf]

# Change in RRF with Concentration (Phenol)

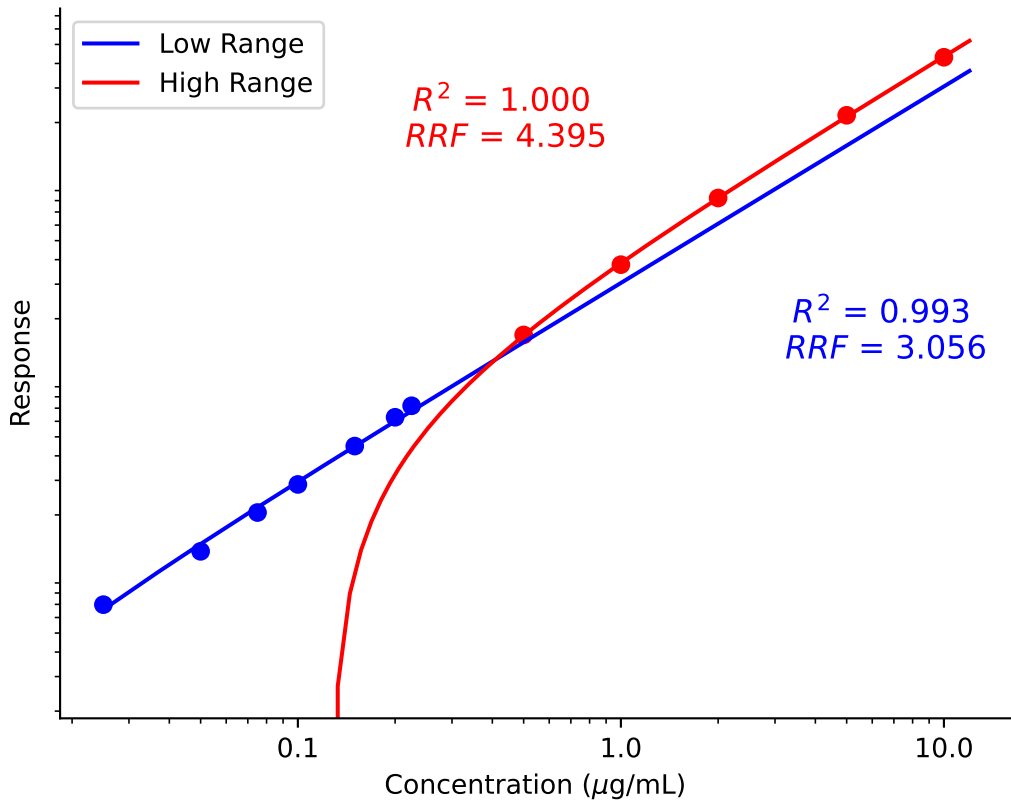

Supplement: Supplementary file 5 [file ac5c04247_si_005.zip › Phenol-SVOCloglog-EWandHigh.pdf]

Change in RRF with Concentration (Phenol)

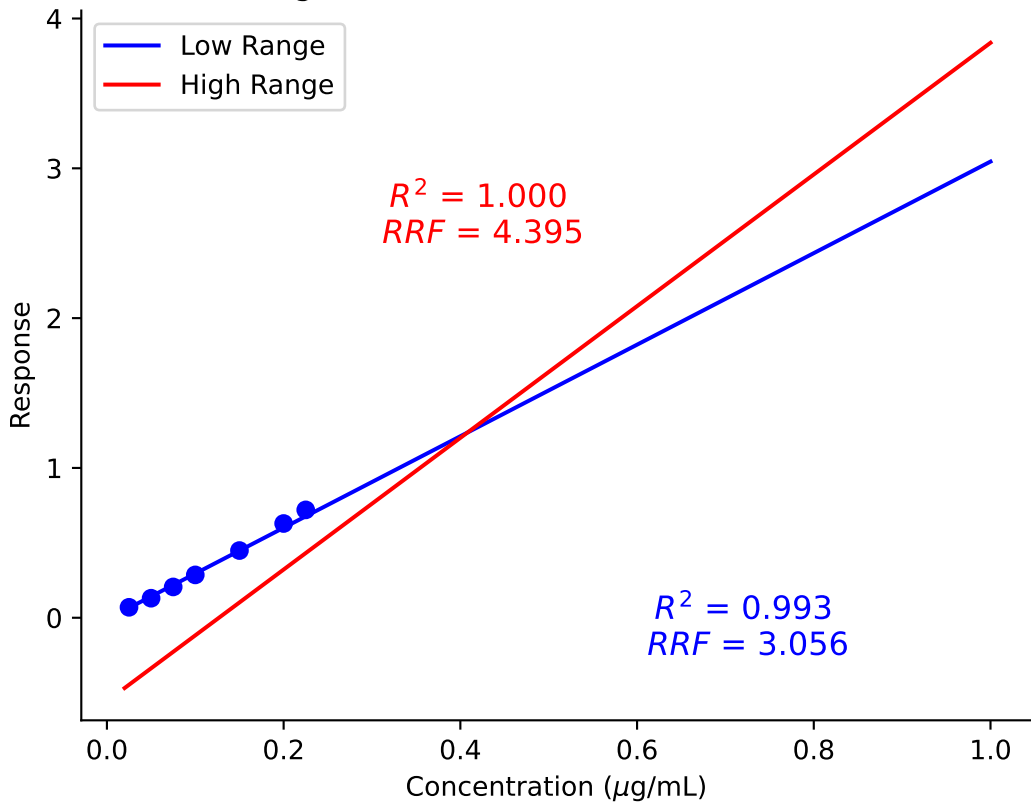

Supplement: Supplementary file 5 [file ac5c04247_si_005.zip › Phenol-SVOC-LowView.pdf]

Change in RRF with Concentration (Tetra(ethylene glycol) diacrylate )

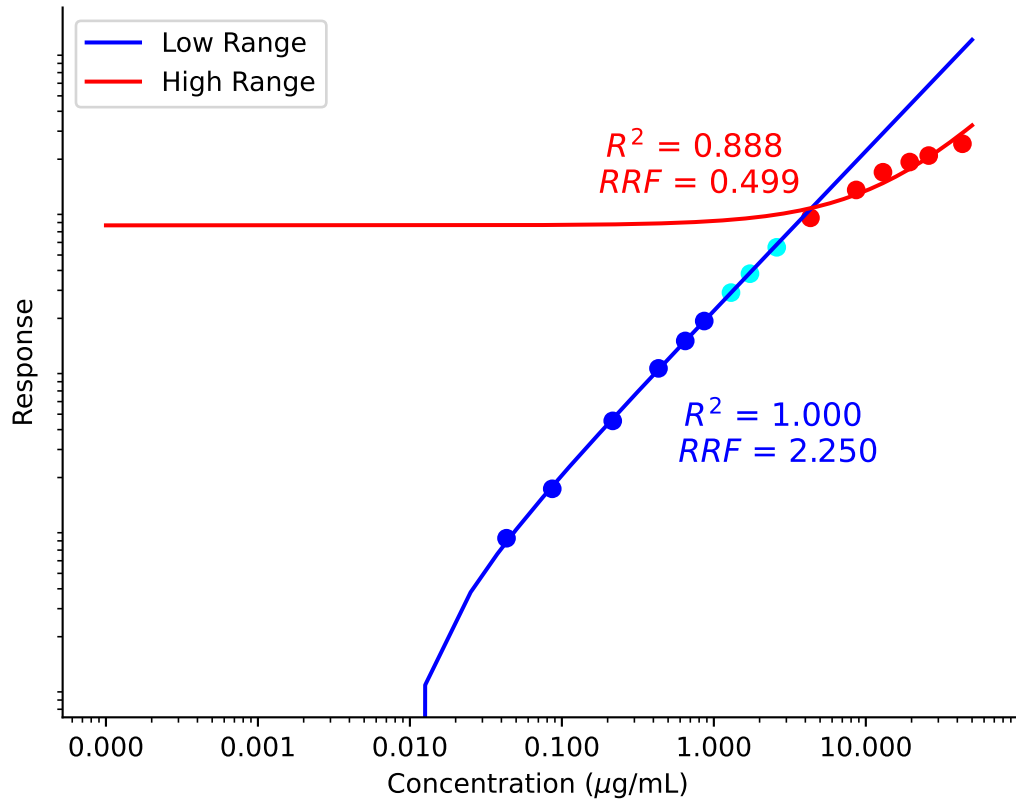

Supplement: Supplementary file 5 [file ac5c04247_si_005.zip › Tetra(ethylene glycol) diacrylate -NVOCloglog-EWandHigh.pdf]

Change in RRF with Concentration (Tetradecyl sulfate sodium salt)

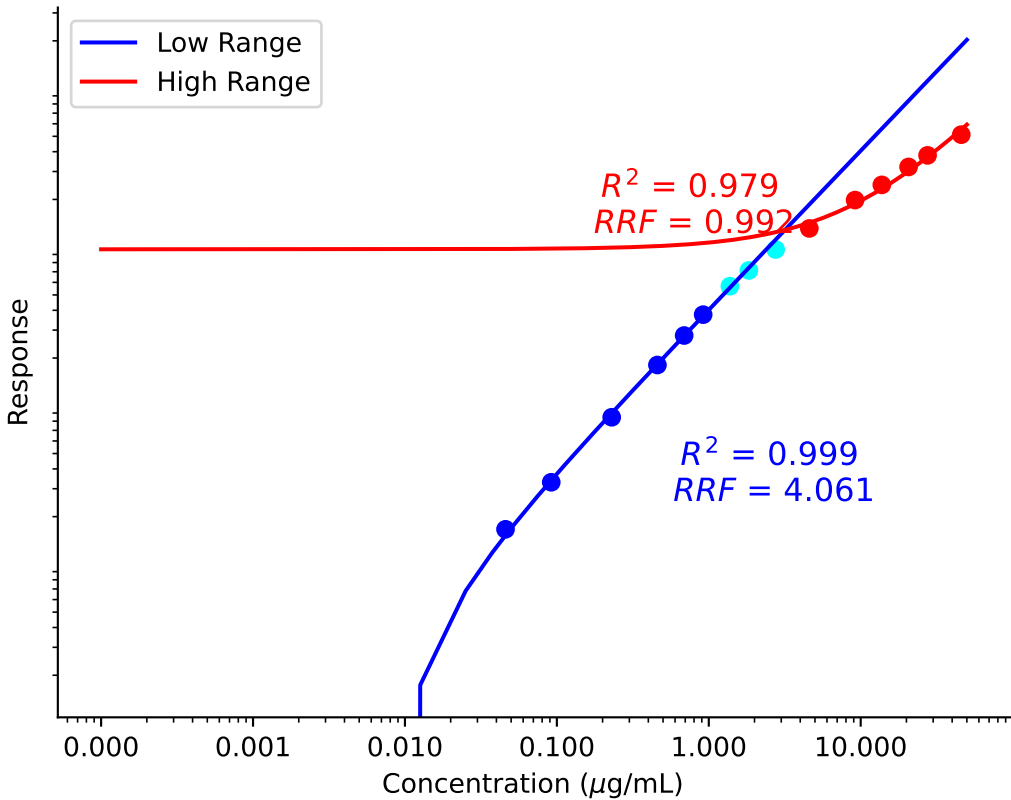

Supplement: Supplementary file 5 [file ac5c04247_si_005.zip › Tetradecyl sulfate sodium salt-NVOCloglog-EWandHigh.pdf]

# Change in RRF with Concentration (Tetraethylene glycol)

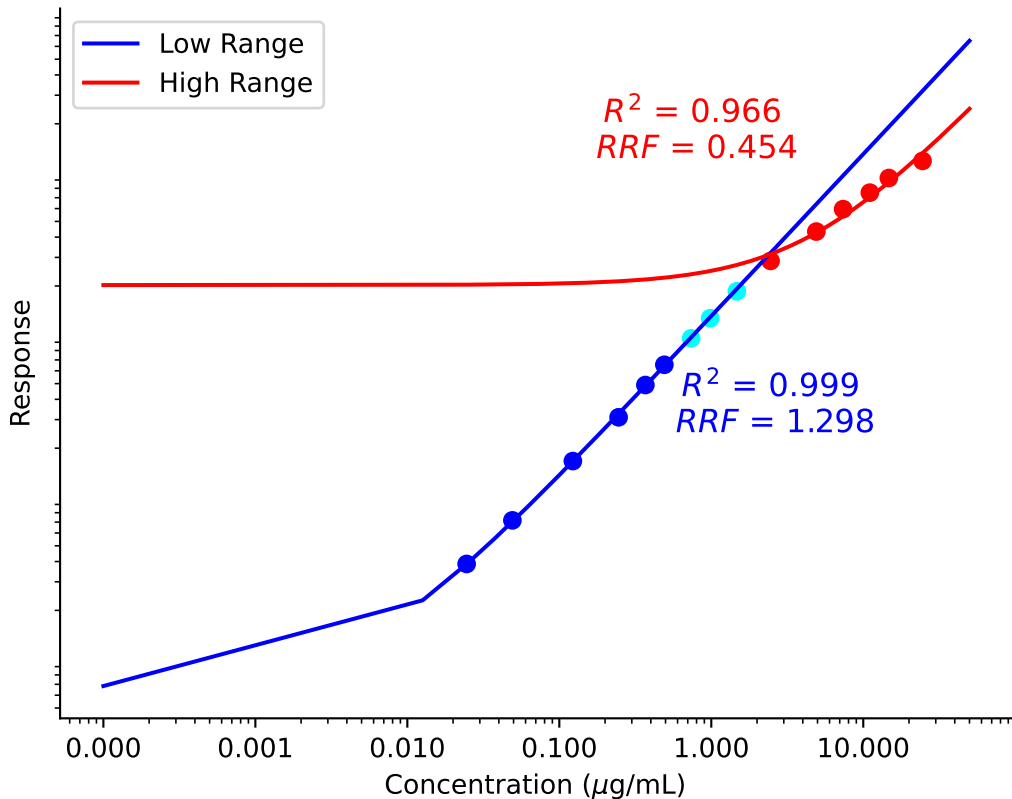

Supplement: Supplementary file 5 [file ac5c04247_si_005.zip › Tetraethylene glycol-NVOCloglog-EWandHigh.pdf]

# Change in RRF with Concentration (Triethyl citrate)

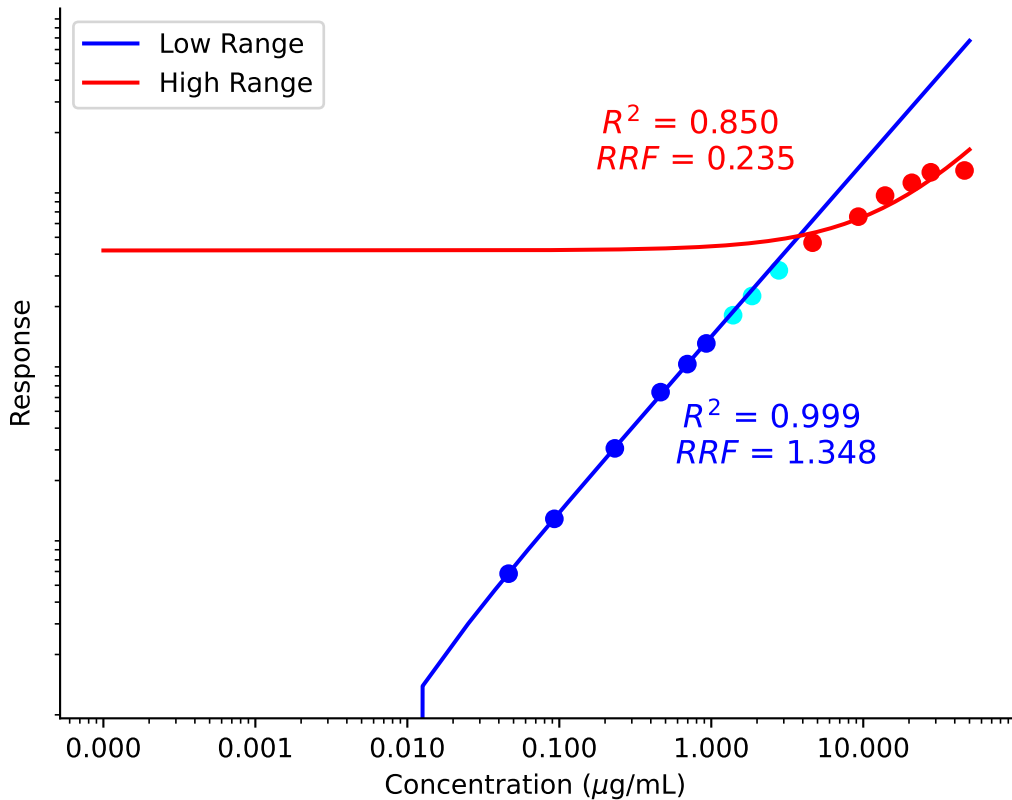

Supplement: Supplementary file 5 [file ac5c04247_si_005.zip › Triethyl citrate-NVOCloglog-EWandHigh.pdf]

# Change in RRF with Concentration (Triphenyl phosphate)

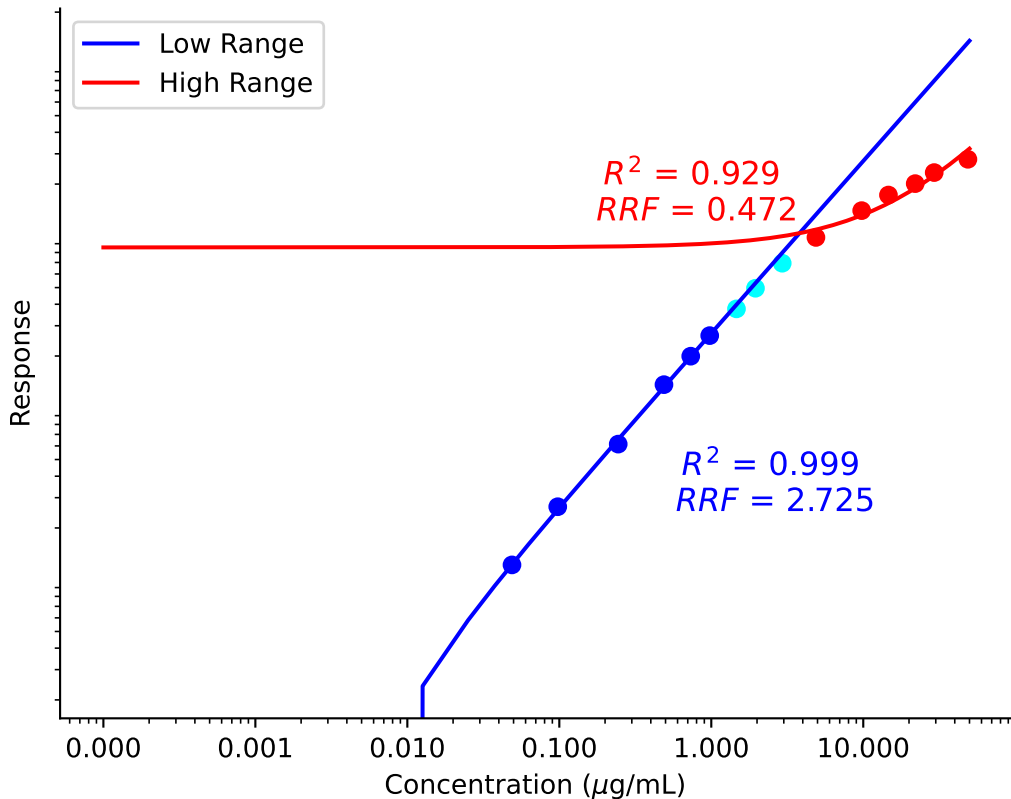

Supplement: Supplementary file 5 [file ac5c04247_si_005.zip › Triphenyl phosphate-NVOCloglog-EWandHigh.pdf]
